# Author Correction: Endogenous IL-1 receptor antagonist restricts healthy and malignant myeloproliferation

**DOI:** 10.1038/s41467-023-39601-3

**Published:** 2023-06-30

**Authors:** Alicia Villatoro, Vincent Cuminetti, Aurora Bernal, Carlos Torroja, Itziar Cossío, Alberto Benguría, Marc Ferré, Joanna Konieczny, Enrique Vázquez, Andrea Rubio, Peter Utnes, Almudena Tello, Xiaona You, Christopher G. Fenton, Ruth H. Paulssen, Jing Zhang, Fátima Sánchez-Cabo, Ana Dopazo, Anders Vik, Endre Anderssen, Andrés Hidalgo, Lorena Arranz

**Affiliations:** 1 Stem Cells, Ageing and Cancer Research Group, Department of Medical Biology, Faculty of Health Sciences, UiT – The Arctic University of Norway, MH2 building level 10, 9019 Tromsø, Norway; 2 Bioinformatics Unit, Fundación Centro Nacional de Investigaciones Cardiovasculares (CNIC), 28029 Madrid, Spain; 3 Area of Cell and Developmental Biology, CNIC, 28029 Madrid, Spain; 4 Genomics Unit, CNIC, 28029 Madrid, Spain; 5 McArdle Laboratory for Cancer Research, University of Wisconsin-Madison, Madison, WI 53705 USA; 6 Genomics Support Center Tromsø, Faculty of Health Sciences, UiT – The Arctic University of Norway, MH building level 9, 9019 Tromsø, Norway; 7 Clinical Bioinformatics Research Group, Department of Clinical Medicine, Faculty of Health Sciences, UiT – The Arctic University of Norway, MH building level 9, 9019 Tromsø, Norway; 8 Department of Hematology, University Hospital of North Norway, 9019 Tromsø, Norway; 9 Associate Investigator, Norwegian Center for Molecular Medicine (NCMM), University of Oslo, 0349 Oslo, Norway

**Keywords:** Leukaemia, Haematopoietic stem cells

Correction to: *Nature Communications* 10.1038/s41467-022-35700-9, published online 03 January 2023

The original version of this Article omitted from the author list the 12^th^ author, Almudena Tello, who is from Stem Cells, Ageing and Cancer Research Group, Department of Medical Biology, Faculty of Health Sciences, UiT – The Arctic University of Norway, MH2 building level 10, 9019 Tromsø, Norway. The corrected version of the Acknowledgements will remove the initials of A. Tello from the original version. Consequently, the initials of A.T were added to the Author Contributions as having ‘performed experiments’.

The original version of this Article also contained an error in the labelling of the t-SNE plots from Figure 6a, which was inverted.

This has now been corrected in both the PDF and HTML versions of the Article.

