# Supplementary material for: Endogenous IL-1 receptor antagonist restricts healthy and malignant myeloproliferation
Source: Nat Commun. 2023 Jan 3;14:12. doi: 10.1038/s41467-022-35700-9 (PMC9810723; doi:10.1038/s41467-022-35700-9)
Supplement: Supplementary file 1 — Supplementary Information [file 41467_2022_35700_MOESM1_ESM.pdf]

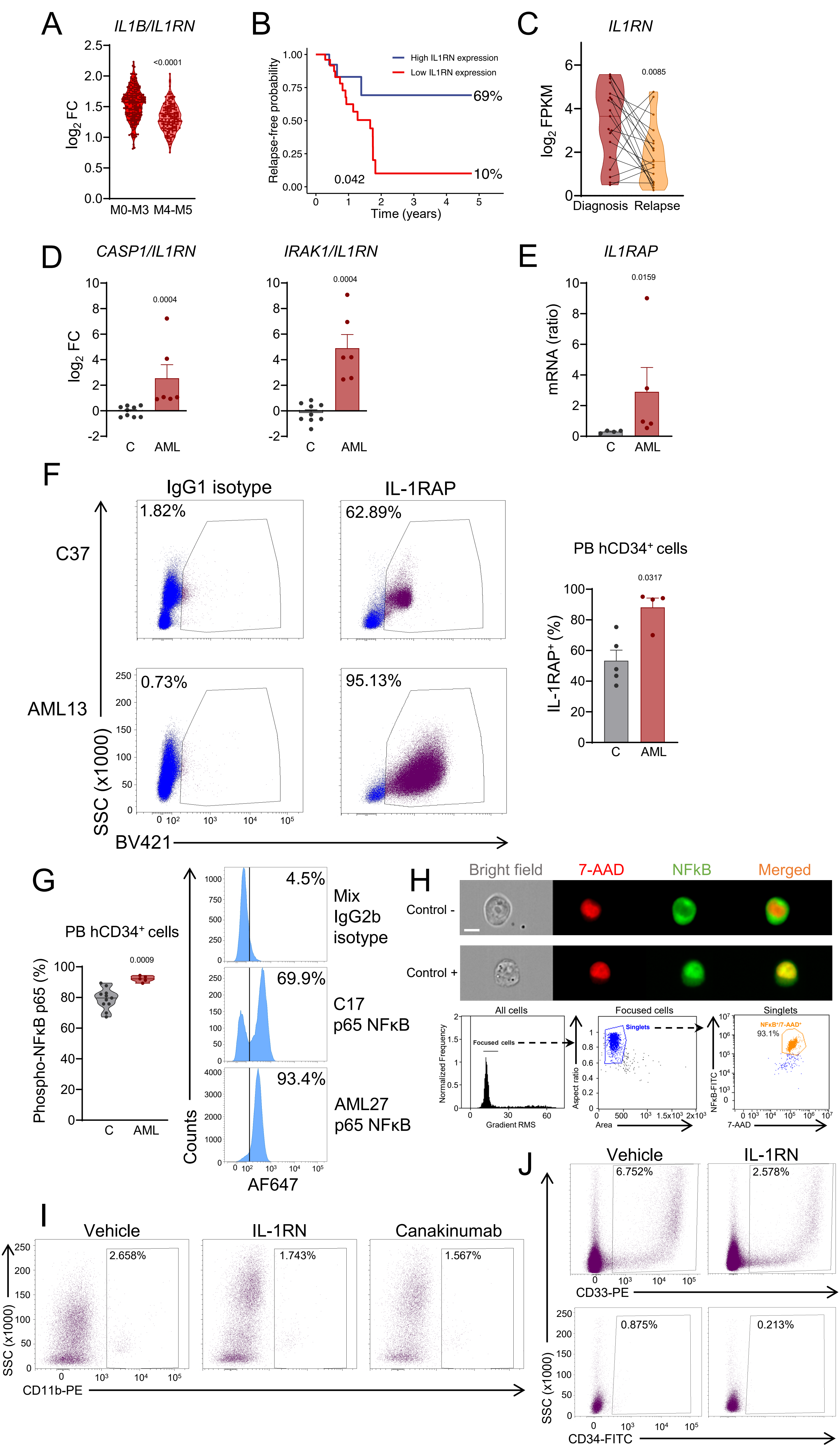
A
IL1B/IL1RN
log2 FC
<0.0001
M0-M3
M4-M5
B
Relapse-free probability
High IL1RN expression
Low IL1RN expression
69%
10%
0.042
Time (years)
C
IL1RN
log2 FPKM
0.0085
Diagnosis
Relapse
D
CASP1/IL1RN
IRAK1/IL1RN
0.0004
C
AML
E
IL1RAP
mRNA (ratio)
0.0159
F
IgG1 isotype
IL-1RAP
C37
AML13
1.82%
62.89%
0.73%
95.13%
SSC (x1000)
BV421
PB hCD34+ cells
IL-1RAP+ (%)
0.0317
G
PB hCD34+ cells
Phospho-NFκB p65 (%)
0.0009
Counts
AF647
4.5%
Mix IgG2b isotype
69.9%
C17 p65 NFκB
93.4%
AML27 p65 NFκB
H
Bright field
7-AAD
NFkB
Merged
Control -
Control +
All cells
Focused cells
Singlets
Normalized Frequency
Gradient RMS
Aspect ratio
Area
NFκB+/7-AAD+
93.1%
NFκB-FITC
7-AAD
I
Vehicle
IL-1RN
Canakinumab
2.658%
1.743%
1.567%
SSC (x1000)
CD11b-PE
J
Vehicle
IL-1RN
6.752%
2.578%
CD33-PE
0.875%
0.213%
SSC (x1000)
CD34-FITC

Supplementary Figure S1 (Related to Figure 1). Low IL-1RN predicts AML progression in patients. (A) Analyses of publicly available gene expression profiling of AML blasts from AML patients (GSE14468). mRNA expression of *IL1B* versus *IL1RN*, comparing AML subtypes M0-M3 (n=224) versus M4-M5 (n=171). FC, fold change. (B-C) Analyses of publicly available RNA sequencing of AML blasts obtained from 19 matched-pair AML patients (dbGaP accession phs001027). (B) AML patient relapse-free probability analyzed as a function of *IL1RN* expression. p value indicates log-rank test. (C) Expression of *IL1RN* in paired diagnosis-relapse AML samples. FPKM, fragments per kilobase per million mapped fragments. (D) qRT-PCR mRNA expression of *CASP1* and *IRAK1* versus *IL1RN* in $CD34^+$ progenitors from volunteers (C, n=9) and AML patients (n=6). (E) qRT-PCR mRNA expression of *IL1RAP* in $CD34^+$ progenitors from volunteers (C, n=4) and AML patients (n=5). (F) Representative fluorescence-activated cell sorting (FACS) analysis (cells in $CD34^+$ cells, %) and frequencies of $CD34^+$ progenitors expressing IL-1 receptor accessory protein (IL-1RAP) in volunteers (C, n=5) and AML patients (n=4). PB, peripheral blood; h, human. (G) Frequencies of $CD34^+$ progenitors positive for phosphorylated NFκB in volunteers (C, n=11) and AML patients (n=5) and representative FACS histograms (cells in $CD34^+$ cells, %). (F-G) Isotype staining was used as negative control. (H) Representative images of bright field, 7-AAD, NFκB, and 7-AAD/NFκB merged channels for THP-1 cells unstimulated (negative control) or stimulated (positive control) with lipopolysaccharide, over 3 independent experiments; scale bar, 10μm, and representative flow cytometry analysis of previous gating (unstimulated cells in culture, %). RMS, root mean square. (I-J) AML xenografts. (I) Representative FACS analysis of human $CD11b^+$ cells in PB from mice treated with vehicle, IL-1RN or canakinumab, over 3 independent experiments (cells in PB, %). (J) Representative FACS analysis of human $CD33^+$ and $CD34^+$ cells in total bone marrow (TBM) of mice treated with vehicle or IL-1RN (cells in TBM, %). Data are biologically independent samples or animals, and means ± S.E.M. for bar plots or medians for violin plots. Statistical analyses were performed with two-tailed Student's *t*-test (A), Kaplan-Meier survival analysis (B), paired two-tailed Student's *t*-test (C) or two-tailed Mann-Whitney U test (D-G). p values ≤ 0.05 are reported. Source data are provided as a Source Data file.

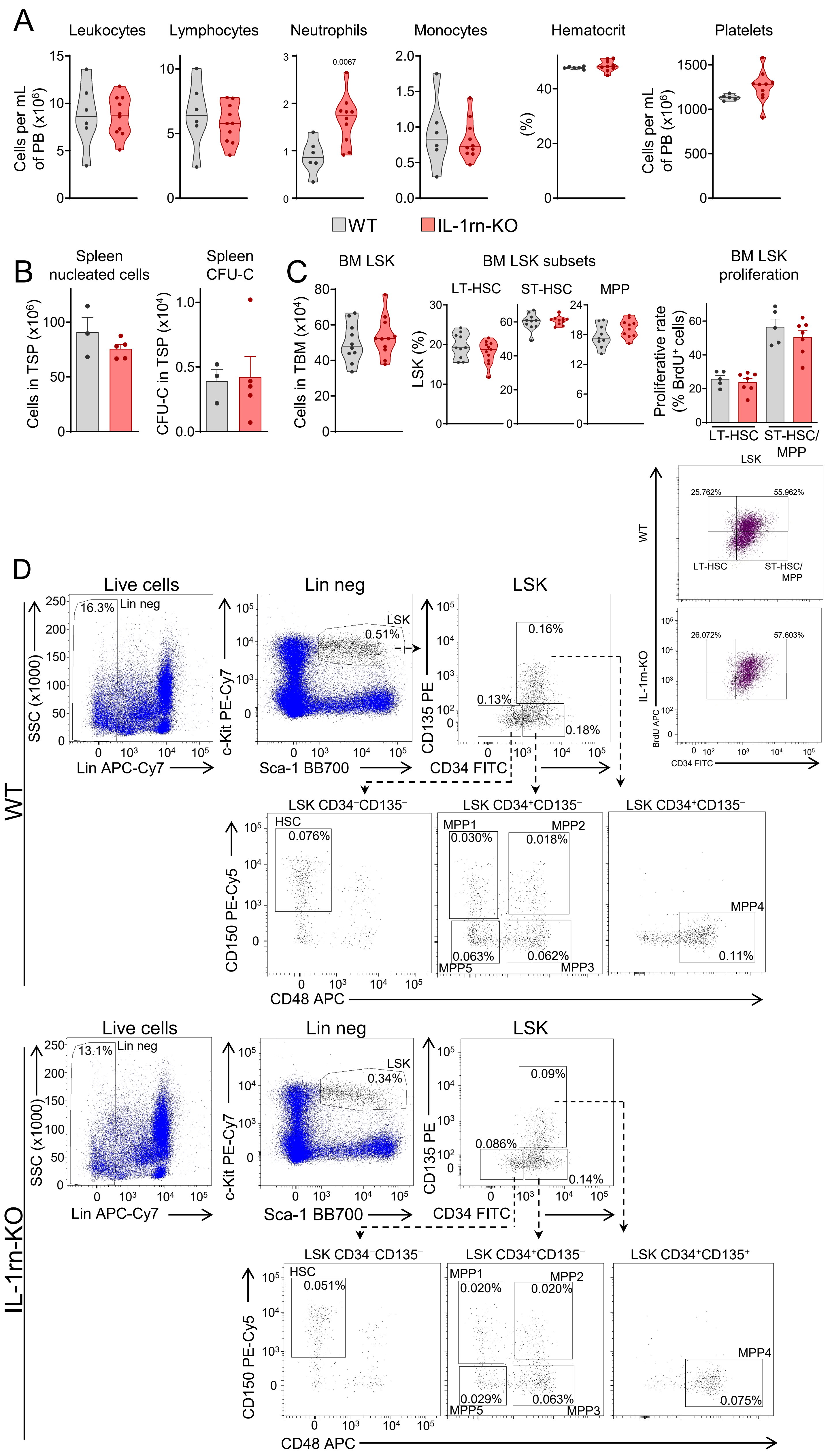

A
Leukocytes
Lymphocytes
Neutrophils
Monocytes
Hematocrit
Platelets
Cells per mL of PB (x10^6)
0.0067
(%)
WT
IL-1rn-KO
B
Spleen nucleated cells
Spleen CFU-C
Cells in TSP (x10^6)
CFU-C in TSP (x10^4)
C
BM LSK
BM LSK subsets
LT-HSC
ST-HSC
MPP
BM LSK proliferation
Cells in TBM (x10^4)
LSK (%)
Proliferative rate (% BrdU+ cells)
LT-HSC
ST-HSC/MPP
D
Live cells
Lin neg
LSK
16.3%
0.51%
0.16%
0.13%
0.18%
SSC (x1000)
Lin APC-Cy7
c-Kit PE-Cy7
Sca-1 BB700
CD135 PE
CD34 FITC
LSK CD34−CD135−
LSK CD34+CD135−
LSK CD34+CD135−
HSC
0.076%
MPP1
0.030%
MPP2
0.018%
MPP4
0.063%
0.062%
0.11%
MPP5
MPP3
CD150 PE-Cy5
CD48 APC
13.1%
0.34%
0.09%
0.086%
0.14%
LSK CD34−CD135−
LSK CD34+CD135−
LSK CD34+CD135+
0.051%
0.020%
0.020%
0.029%
0.063%
0.075%
25.762%
55.962%
LT-HSC
ST-HSC/MPP
26.072%
57.603%
BrdU APC
CD34 FITC

Supplementary Figure S2 (Related to Figure 2). Deletion of IL-1rn triggers IL-1β-induced myelopoiesis in the absence of immunogenic stimulus. (A) Peripheral blood (PB) counts from C57BL/6J wild-type (WT, n=6; platelets n=5) and IL-1rn knockout (IL-1rn-KO, n=10; hematocrit n=9) mice. (B) Total number of spleen nucleated cells (TSP) and SP colony-forming unit cells (CFU-C) from WT (n=3) and IL-1rn-KO (n=5) mice. (C) Total bone marrow (TBM) number of $Lin^{-}c\text{-}Kit^{+}Sca\text{-}1^{+}$ (LSK) cells (WT, n=10; IL-1rn-KO, n=11), frequencies of BM LSK subsets: LSK $CD34^{-}Flt3^{-}$, long-term hematopoietic stem cells (LT-HSC) (WT, n=10; IL-1rn-KO, n=11); LSK $CD34^{+}Flt3^{-}$, short-term HSC (ST-HSC) (WT, n=10; IL-1rn-KO, n=10); LSK $CD34^{+}Flt3^{+}$, multipotent progenitors (MPP) (WT, n=9; IL-1rn-KO, n=10), and (up) proliferative rate in BM LSK subsets $CD34^{-}$ (LT-HSC) and $CD34^{+}$ (ST-HSC/MPP) (WT, n=5; IL-1rn-KO, n=7) and (down) representative fluorescence-activated cell sorting (FACS) analysis (cells in LT-HSC or ST-HSC/ MPP, %). (D) Representative FACS analysis (cells in TBM, %) of BM hematopoietic stem and progenitor cell subsets corresponding to HSC (LSK $CD34^{-}Flt3^{-}CD48^{-}CD150^{+}$), MPP1 (LSK $CD34^{+}Flt3^{-}CD48^{-}CD150^{+}$), MPP2 (LSK $CD34^{+}Flt3^{-}CD48^{+}CD150^{+}$), MPP3 (LSK $CD34^{+}Flt3^{-}CD48^{+}CD150^{-}$), MPP4 (LSK $CD34^{+}Flt3^{+}CD48^{+}$ $CD150^{-}$) and MPP5 (LSK $CD34^{+}Flt3^{-}CD48^{-}CD150^{-}$) from WT and IL-1rn-KO mice. Data are biologically independent animals, and means ± S.E.M for bar plots or medians for violin plots. Statistical analyses were performed with two-tailed Student's *t*-test. p values ≤ 0.05 are reported. Source data are provided as a Source Data file.

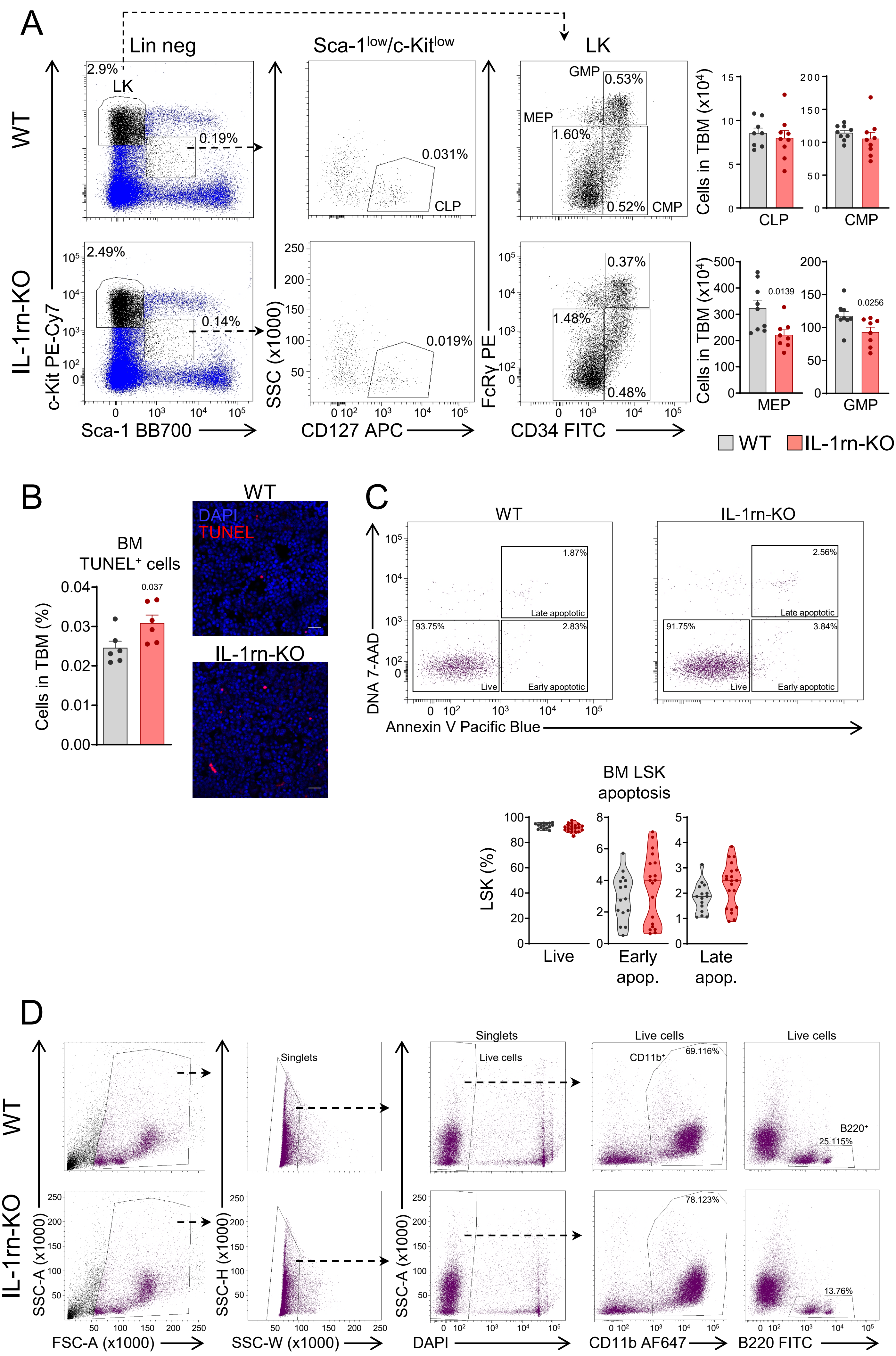
A
Lin neg
Sca-1low/c-Kitlow
LK
WT
IL-1rn-KO
2.9%
LK
0.19%
0.031%
CLP
GMP
0.53%
MEP
1.60%
0.52%
CMP
2.49%
0.14%
0.019%
0.37%
1.48%
0.48%
c-Kit PE-Cy7
Sca-1 BB700
SSC (x1000)
CD127 APC
FcRγ PE
CD34 FITC
Cells in TBM (x10^4)
CLP
CMP
MEP
GMP
0.0139
0.0256
WT
IL-1rn-KO
B
BM
TUNEL+ cells
0.037
Cells in TBM (%)
WT
DAPI
TUNEL
IL-1rn-KO
C
WT
IL-1rn-KO
1.87%
Late apoptotic
93.75%
2.83%
Live
Early apoptotic
2.56%
91.75%
3.84%
DNA 7-AAD
Annexin V Pacific Blue
BM LSK
apoptosis
LSK (%)
Live
Early apop.
Late apop.
D
WT
IL-1rn-KO
Singlets
Live cells
CD11b+
69.116%
B220+
25.115%
78.123%
13.76%
SSC-A (x1000)
FSC-A (x1000)
SSC-H (x1000)
SSC-W (x1000)
DAPI
CD11b AF647
B220 FITC

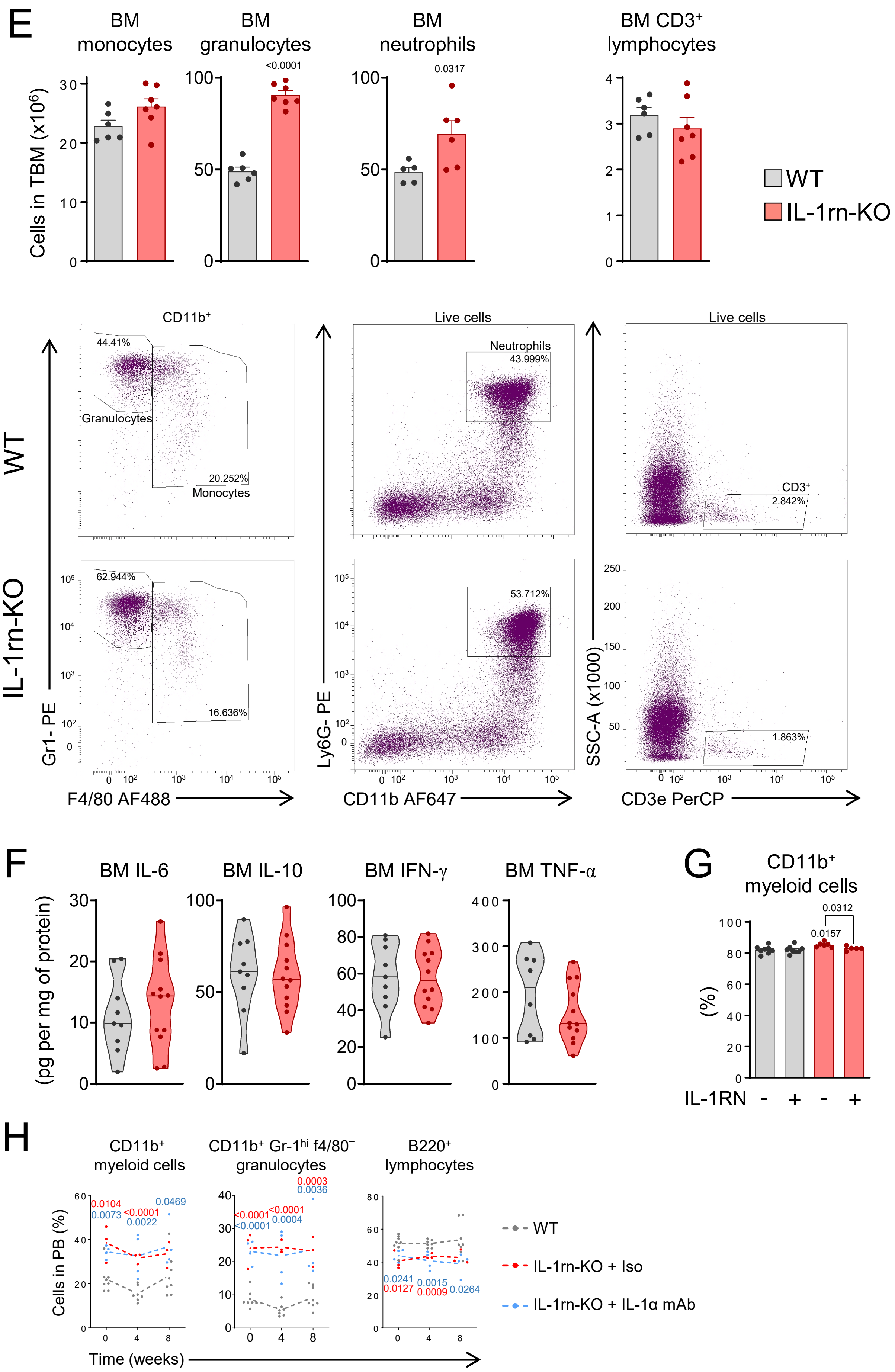
E
BM monocytes
BM granulocytes
BM neutrophils
BM CD3+ lymphocytes
Cells in TBM (x10^6)
<0.0001
0.0317
WT
IL-1rn-KO
CD11b+
Live cells
44.41%
Granulocytes
20.252%
Monocytes
Neutrophils
43.999%
CD3+
2.842%
62.944%
16.636%
53.712%
1.863%
Gr1- PE
F4/80 AF488
Ly6G- PE
CD11b AF647
SSC-A (x1000)
CD3e PerCP
F
BM IL-6
BM IL-10
BM IFN-γ
BM TNF-α
(pg per mg of protein)
G
CD11b+ myeloid cells
(%)
0.0312
0.0157
IL-1RN - + - +
H
CD11b+ myeloid cells
CD11b+ Gr-1hi f4/80− granulocytes
B220+ lymphocytes
Cells in PB (%)
Time (weeks)
0.0104
0.0073
<0.0001
0.0022
0.0469
0.0003
0.0036
<0.0001
<0.0001
<0.0001
0.0004
0.0241
0.0127
0.0015
0.0009
0.0264
WT
IL-1rn-KO + Iso
IL-1rn-KO + IL-1α mAb

Supplementary Figure S3 (Related to Figure 2). Deletion of IL-1rn triggers IL-1β-induced myelopoiesis in the absence of immunogenic stimulus. (A) Representative fluorescence-activated cell sorting (FACS) analysis (cells in total bone marrow; TBM, %) and total bone marrow (TBM) number of common lymphoid progenitors (CLP, Lin^{-}c-Kit^{low}Sca-$1^{low}CD127^{+}$), common myeloid progenitors (CMP, Lin^{-}c-Kit^{+}Sca-1^{-} (LK) $CD34^{+}FcR\gamma^{-}$), megakaryocyte erythroid progenitors (MEP, LK $CD34^{-}FcR\gamma^{-}$) and granulocyte-monocyte progenitors (GMP, LK $CD34^{+}FcR\gamma^{+}$) from C57BL/6J wild-type (WT) and IL-1rn knockout (IL-1rn-KO) mice. (B) Apoptotic rate in TBM (n=6 per group), and representative TUNEL (terminal deoxynucleotidyl transferase dUTP nick end labeling staining, red) of BM sections; nuclei were counterstained with DAPI (blue). Scale bar, 20 µm. (C) Representative FACS analysis to identify the fraction of live, early apoptotic and late apoptotic cells within the Lin^{-}c-Kit^{+}Sca-1^{+} (LSK) compartment in the BM from WT and IL-1rn-KO mice (cells in LSK cells, %), and apoptotic rate within the BM LSK compartment (Live: WT n=15, IL-1rn-KO n=20; Early apoptotic: WT n=15, IL-1rn-KO n=19; Late apoptotic: WT n=15, IL-1rn-KO n=20). (D) Representative FACS analysis of $CD11b^{+}$ myeloid cells and $B220^{+}$ lymphocytes in the BM of WT and IL-1rn-KO mice (cells in TBM, %). (E) TBM number of $CD11b^{+}$Gr-1^{+}f4/80^{+} monocytes, $CD11b^{+}$Gr-1^{hi}f4/80^{-} granulocytes, $CD11b^{+}Ly6G^{+}$ neutrophils and $CD3^{+}$ lymphocytes from WT (n=6; neutrophils n=5) and IL-1rn-KO (n=7, neutrophils n=6) mice, and representative FACS analysis (cells in TBM, %). (F) Cytokine levels in BM extracellular fluid from WT (n=9, TNF-α n=8) and IL-1rn-KO mice (n=12). (G) Frequency of $CD11b^{+}$ myeloid cells differentiated *ex vivo* from lineage-negative progenitors from the BM of WT (n=8) and IL-1rn-KO (vehicle (-), n=6; IL-1RN (+), n=5) mice. (H) Frequency evolution of $CD11b^{+}$ myeloid cells, $CD11b^{+}$Gr-1^{hi}f4/80^{-} granulocytes and $B220^{+}$ lymphocytes in peripheral blood (PB) of WT (n=8) and IL-1rn-KO mice treated with IgG isotype (Iso; n=3) or IL-1α monoclonal antibody (IL-1α mAb; n=4). Data are biologically independent animals, and means ± S.E.M for bar plots or medians for violin plots. Statistical analyses were performed with two-tailed Student's *t*-test. p values ≤ 0.05 are reported. Source data are provided as a Source Data file.

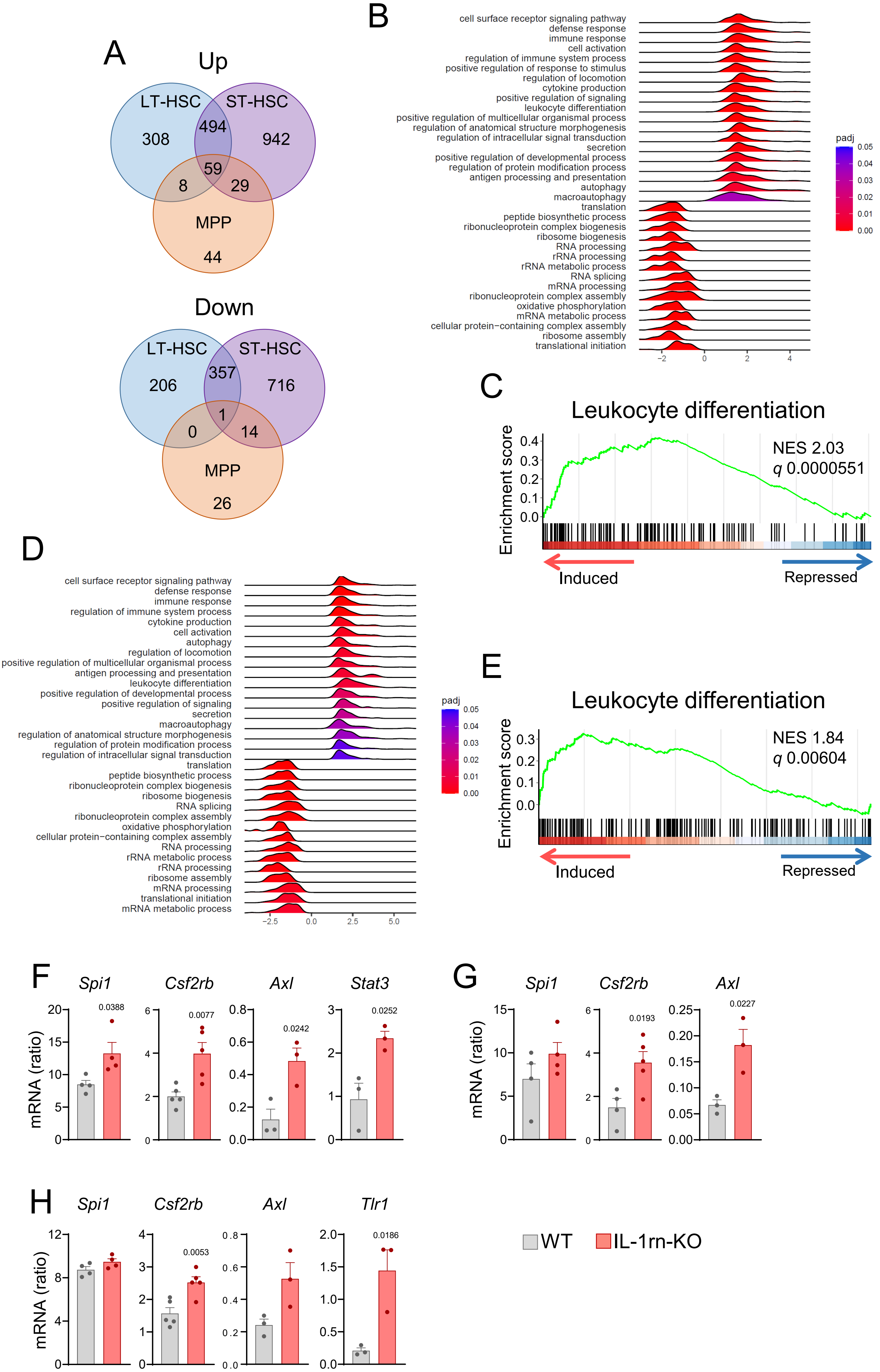
A
Up
LT-HSC
ST-HSC
308
494
942
59
8
29
MPP
44
Down
LT-HSC
ST-HSC
206
357
716
1
0
14
MPP
26
B
cell surface receptor signaling pathway
defense response
immune response
cell activation
regulation of immune system process
positive regulation of response to stimulus
regulation of locomotion
cytokine production
positive regulation of signaling
leukocyte differentiation
positive regulation of multicellular organismal process
regulation of anatomical structure morphogenesis
regulation of intracellular signal transduction
secretion
positive regulation of developmental process
regulation of protein modification process
antigen processing and presentation
autophagy
macroautophagy
translation
peptide biosynthetic process
ribonucleoprotein complex biogenesis
ribosome biogenesis
RNA processing
rRNA processing
rRNA metabolic process
RNA splicing
mRNA processing
ribonucleoprotein complex assembly
oxidative phosphorylation
mRNA metabolic process
cellular protein−containing complex assembly
ribosome assembly
translational initiation
padj
C
Leukocyte differentiation
Enrichment score
NES 2.03
q 0.0000551
Induced
Repressed
D
cell surface receptor signaling pathway
defense response
immune response
regulation of immune system process
cytokine production
cell activation
autophagy
regulation of locomotion
positive regulation of multicellular organismal process
antigen processing and presentation
leukocyte differentiation
positive regulation of developmental process
positive regulation of signaling
secretion
macroautophagy
regulation of anatomical structure morphogenesis
regulation of protein modification process
regulation of intracellular signal transduction
translation
peptide biosynthetic process
ribonucleoprotein complex biogenesis
ribosome biogenesis
RNA splicing
ribonucleoprotein complex assembly
oxidative phosphorylation
cellular protein−containing complex assembly
RNA processing
rRNA metabolic process
rRNA processing
ribosome assembly
mRNA processing
translational initiation
mRNA metabolic process
padj
E
Leukocyte differentiation
Enrichment score
NES 1.84
q 0.00604
Induced
Repressed
F
Spi1
Csf2rb
Axl
Stat3
mRNA (ratio)
0.0388
0.0077
0.0242
0.0252
G
Spi1
Csf2rb
Axl
mRNA (ratio)
0.0193
0.0227
H
Spi1
Csf2rb
Axl
Tlr1
mRNA (ratio)
0.0053
0.0186
WT
IL-1rn-KO

I

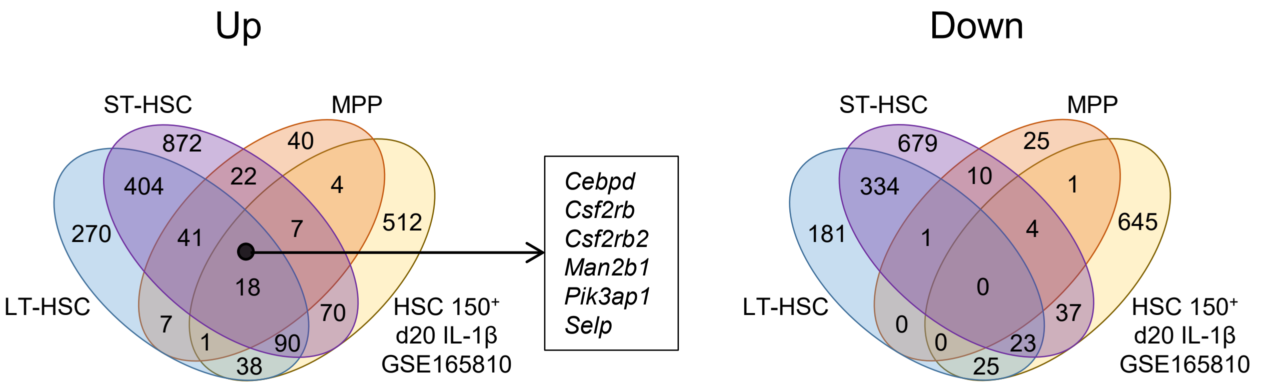
Up
Down
ST-HSC
MPP
LT-HSC
HSC 150+
d20 IL-1β
GSE165810
872
40
404
22
4
270
41
7
512
18
7
1
90
70
38
Cebpd
Csf2rb
Csf2rb2
Man2b1
Pik3ap1
Selp
679
25
334
10
1
181
1
4
645
0
0
0
23
37
25

J

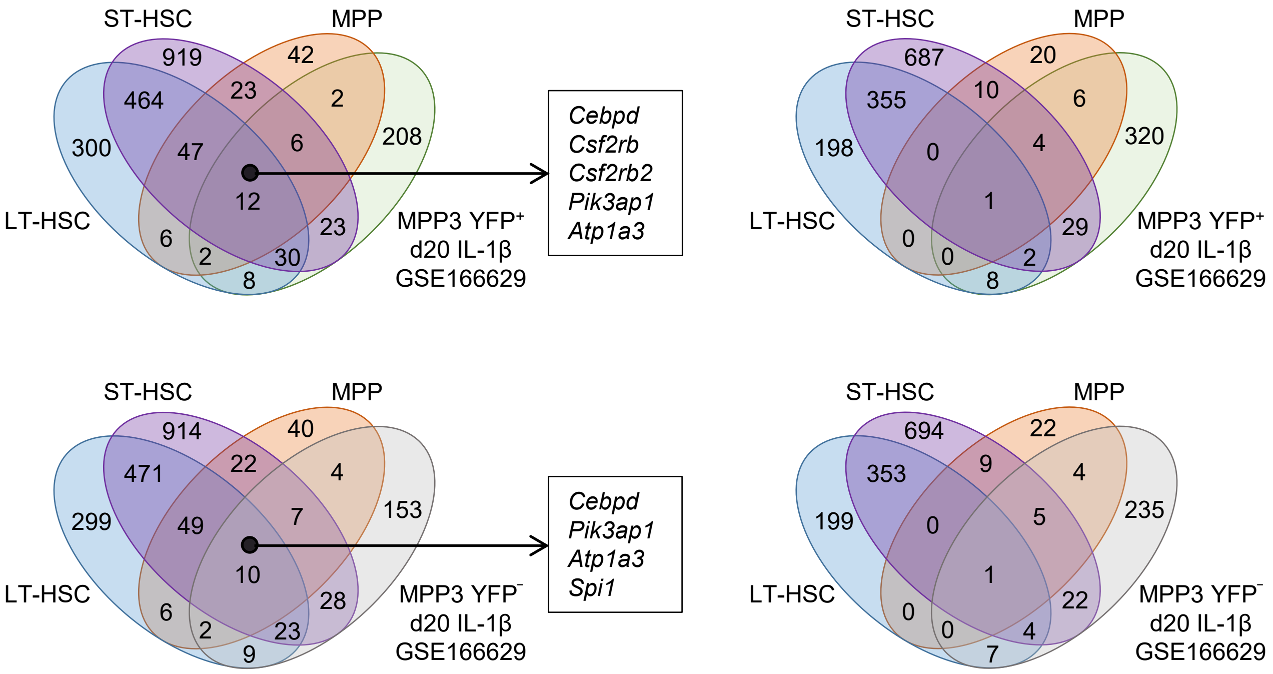
ST-HSC
MPP
LT-HSC
MPP3 YFP+
d20 IL-1β
GSE166629
919
42
464
23
2
300
47
6
208
12
6
2
30
23
8
Cebpd
Csf2rb
Csf2rb2
Pik3ap1
Atp1a3
687
20
355
10
6
198
0
4
320
1
0
0
2
29
8
MPP3 YFP-
914
40
471
22
4
299
49
7
153
10
6
2
23
28
9
Cebpd
Pik3ap1
Atp1a3
Spi1
694
22
353
9
4
199
0
5
235
1
0
0
4
22
7

Supplementary Figure S4 (Related to Figure 3). IL-1β-induced myelopoiesis after IL-1rn deletion is mediated by NFκB activation. (A-E) RNA sequencing (RNA-Seq) in bone marrow $Lin^{-}c\text{-}Kit^{+}Sca\text{-}1^{+}$ (LSK) subsets; LSK $CD34^{-}Flt3^{-}$, long-term hematopoietic stem cells (LT-HSC); LSK $CD34^{+}Flt3^{-}$, short-term HSC (ST-HSC); LSK $CD34^{+}Flt3^{+}$, multipotent progenitors (MPP), from C57BL/6J wild type (WT) and IL-1rn knockout (IL-1rn-KO) mice (n=3 per group). (A) Venn diagrams show number of up- and down- regulated genes in LT-HSC, ST-HSC and MPP from IL-1rn-KO mice versus WT, and overlap of genes. (B-E) Gene Ontology biological processes and kernel density plots showing differences in core enriched gene sets in (B) LT-HSC and (D) ST-HSC, selected within gene set enrichment analysis (GSEA) enriched categories in common. Enrichment plot of coordinated changes of genes induced or repressed in leukocyte differentiation in (C) LT-HSC and (E) ST-HSC. NES, normalized enrichment score. (F-H) qRT-PCR validation of selected RNA-Seq hits in independent biological samples, in (F) LT-HSC, (G) ST-HSC and (H) MPP (*Spi1*: n=4 per group; *Csf2rb*: WT, n=5; ST-HSC n=4; IL-1rn-KO, n=5; *Axl*, *Stat3*, *Tlr1*: n=3 per group). (I) Venn diagrams show number of up- and down-regulated genes in LT-HSC, ST-HSC and MPP from IL-1rn-KO mice versus C57BL/6J WT and in LSK $Flt3^{-}CD48^{-}CD150^{+}$ cells from C57BL/6 WT mice exposed to chronic IL-1β versus vehicle in GSE165810 (n=4 per group). Overlap of genes, and up-regulated genes shared by all LSK subsets identified as NFκB targets from Synapse ID syn4956655, https://bioinfo.lifl.fr/NF-KB and https://www.bu.edu/nf-kb/gene-resources/target-genes (Supplementary Data 1). (J) Venn diagrams show number of up- and down-regulated genes in LT-HSC, ST-HSC and MPP from IL-1rn-KO mice versus C57BL/6 WT and in LSK $Flt3^{-}CD48^{+}CD150^{-}$ (MPP3) from CD45.1 recipient mice transplanted with LSK (YFP^{+}) and competitor whole bone marrow cells (YFP^{-}) and exposed to chronic IL-1β versus vehicle in GSE166629 (n=3 per group). Overlap of genes, and up-regulated genes shared by all LSK subsets identified as NFκB targets. Data in (F-H) are biologically independent animals, and means ± S.E.M. in bar plots. Statistical analyses were performed with GSEA with Benjamini-Hochberg correction for multiple comparisons (B, D), or two-tailed Student's *t*-test (F-H). Adjusted $p < 0.05$ (B, D) or p values ≤ 0.05 (F-H) are reported. Source data are provided as a Source Data file.

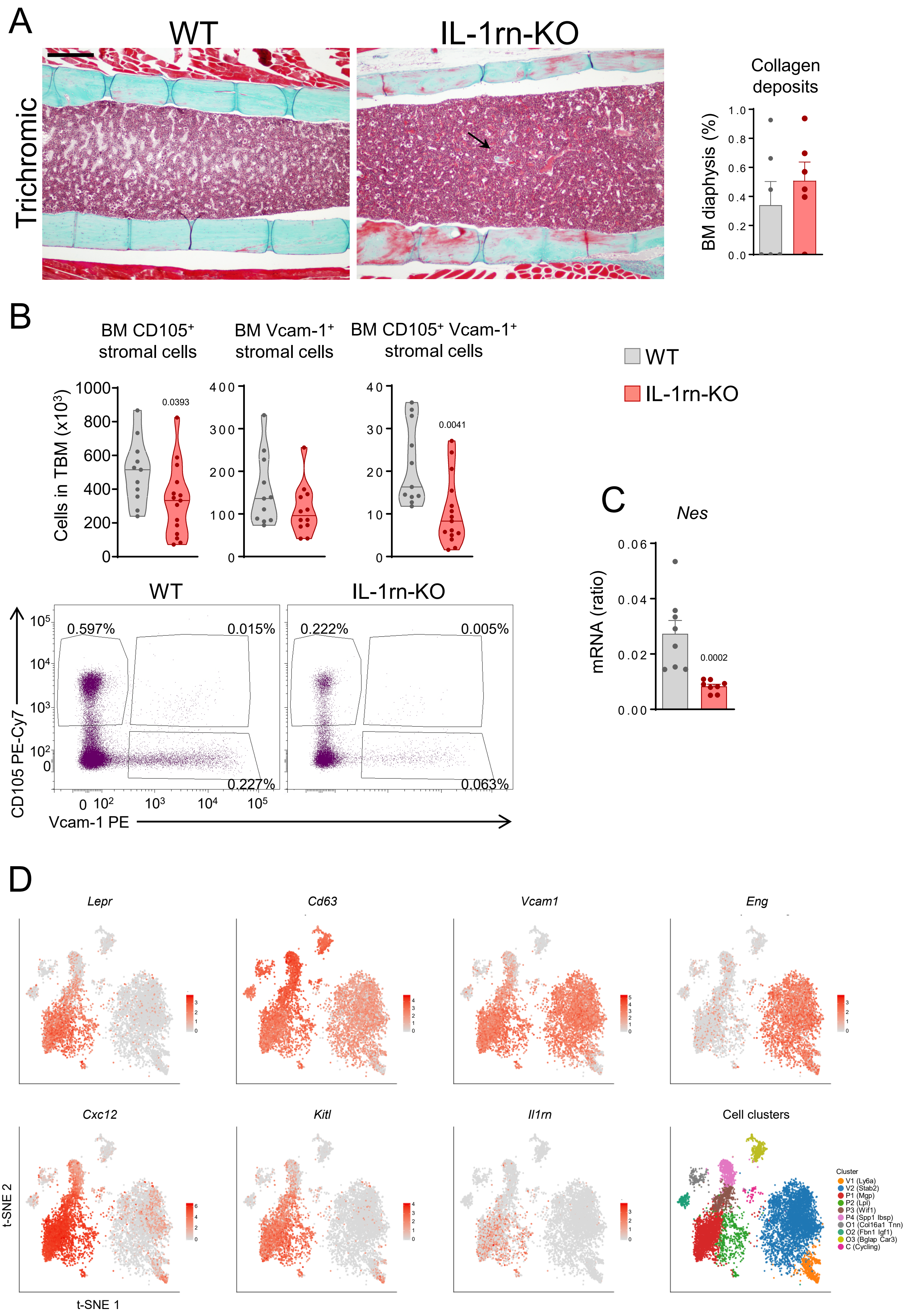
A
WT
IL-1rn-KO
Trichromic
Collagen deposits
BM diaphysis (%)
B
BM CD105+ stromal cells
BM Vcam-1+ stromal cells
BM CD105+ Vcam-1+ stromal cells
Cells in TBM (x10³)
0.0393
0.0041
WT
IL-1rn-KO
0.597%
0.015%
0.227%
0.222%
0.005%
0.063%
CD105 PE-Cy7
Vcam-1 PE
C
Nes
mRNA (ratio)
0.0002
D
Lepr
Cd63
Vcam1
Eng
Cxc12
Kitl
Il1rn
Cell clusters
t-SNE 2
t-SNE 1
Cluster
V1 (Ly6a)
V2 (Stab2)
P1 (Mgp)
P2 (Lpl)
P3 (Wif1)
P4 (Spp1 Ibsp)
O1 (Col16a1 Tnn)
O2 (Fbn1 Igf1)
O3 (Bglap Car3)
C (Cycling)

Supplementary Figure S5 (Related to Figure 4). Deletion of IL-1rn causes IL-1β-induced damage to the bone marrow stroma in the absence of immunogenic stimulus. (A) Representative Masson's trichrome staining of femoral bone marrow (BM) sections; scale bar, 500 µm, and quantification of collagen deposits (arrow) in BM diaphysis from C57BL/6J wild-type (WT) and IL-1rn knockout (IL-1rn-KO) mice (n=6 per group). (B) Total BM (TBM) number and representative fluorescence-activated cell sorting analysis (cells in TBM, %) of $CD45^{-}CD31^{-}Ter119^{-}CD105^{+}$ (WT, n=11; IL-1rn-KO, n=15), $CD45^{-}CD31^{-}Ter119^{-}Vcam\text{-}1^{+}$ (WT, n=11; IL-1rn-KO, n=12) and $CD45^{-}CD31^{-}Ter119^{-}CD105^{+}Vcam\text{-}1^{+}$ (WT, n=11; IL-1rn-KO, n=15) stromal cells. (C) qRT-PCR mRNA expression of *Nes* in BM nucleated cells (n=8 per group). (D) t-SNE plots visualizing expression of selected genes from publicly available single-cell RNA sequencing analysis to characterize mouse BM mesenchymal stromal cells (GSE108892). Vascular cells (VE-Cad^{+}, V1-2; perivascular LEPR^{+} cells (LEPR^{+}, P1-4); osteoblasts (COL2.3^{+}, O1-3). Color bar, log1p(TP10K). Data are biologically independent animals, and means ± S.E.M. for bar plots or medians for violin plots, except (D). Statistical analyses were performed with two-tailed Student's *t*-test (B) or two-tailed Mann-Whitney U test (C). p values ≤ 0.05 are reported. Source data are provided as a Source Data file.

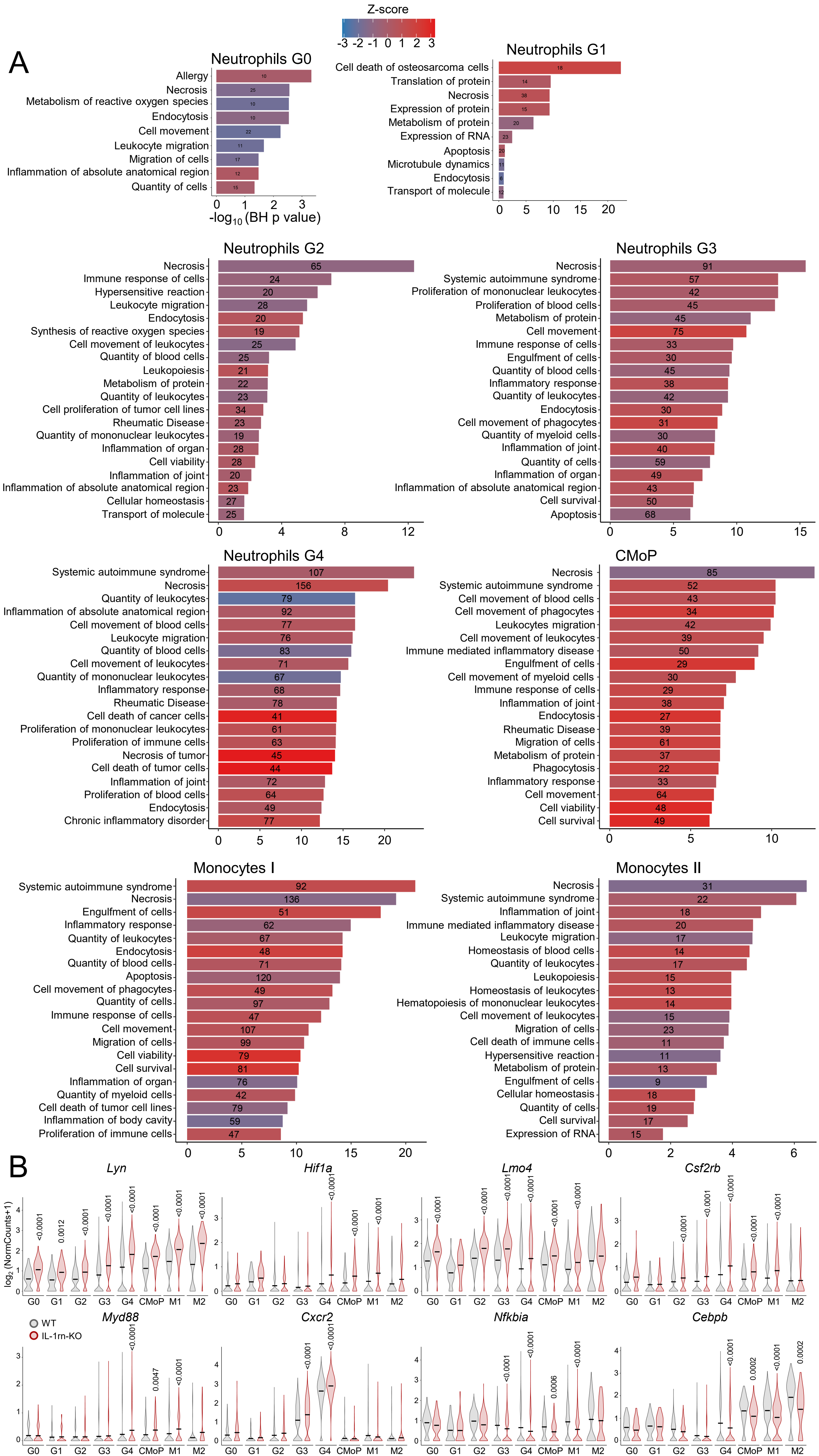
Z-score
-3 -2 -1 0 1 2 3
A
Neutrophils G0
Allergy
Necrosis
Metabolism of reactive oxygen species
Endocytosis
Cell movement
Leukocyte migration
Migration of cells
Inflammation of absolute anatomical region
Quantity of cells
-log10 (BH p value)
Neutrophils G1
Cell death of osteosarcoma cells
Translation of protein
Necrosis
Expression of protein
Metabolism of protein
Expression of RNA
Apoptosis
Microtubule dynamics
Endocytosis
Transport of molecule
Neutrophils G2
Necrosis 65
Immune response of cells 24
Hypersensitive reaction 20
Leukocyte migration 28
Endocytosis 20
Synthesis of reactive oxygen species 19
Cell movement of leukocytes 25
Quantity of blood cells 25
Leukopoiesis 21
Metabolism of protein 22
Quantity of leukocytes 23
Cell proliferation of tumor cell lines 34
Rheumatic Disease 23
Quantity of mononuclear leukocytes 19
Inflammation of organ 28
Cell viability 28
Inflammation of joint 20
Inflammation of absolute anatomical region 23
Cellular homeostasis 27
Transport of molecule 25
Neutrophils G3
Necrosis 91
Systemic autoimmune syndrome 57
Proliferation of mononuclear leukocytes 42
Proliferation of blood cells 45
Metabolism of protein 45
Cell movement 75
Immune response of cells 33
Engulfment of cells 30
Quantity of blood cells 45
Inflammatory response 38
Quantity of leukocytes 42
Endocytosis 30
Cell movement of phagocytes 31
Quantity of myeloid cells 30
Inflammation of joint 40
Quantity of cells 59
Inflammation of organ 49
Inflammation of absolute anatomical region 43
Cell survival 50
Apoptosis 68
Neutrophils G4
Systemic autoimmune syndrome 107
Necrosis 156
Quantity of leukocytes 79
Inflammation of absolute anatomical region 92
Cell movement of blood cells 77
Leukocyte migration 76
Quantity of blood cells 83
Cell movement of leukocytes 71
Quantity of mononuclear leukocytes 67
Inflammatory response 68
Rheumatic Disease 78
Cell death of cancer cells 41
Proliferation of mononuclear leukocytes 61
Proliferation of immune cells 63
Necrosis of tumor 45
Cell death of tumor cells 44
Inflammation of joint 72
Proliferation of blood cells 64
Endocytosis 49
Chronic inflammatory disorder 77
CMoP
Necrosis 85
Systemic autoimmune syndrome 52
Cell movement of blood cells 43
Cell movement of phagocytes 34
Leukocytes migration 42
Cell movement of leukocytes 39
Immune mediated inflammatory disease 50
Engulfment of cells 29
Cell movement of myeloid cells 30
Immune response of cells 29
Inflammation of joint 38
Endocytosis 27
Rheumatic Disease 39
Migration of cells 61
Metabolism of protein 37
Phagocytosis 22
Inflammatory response 33
Cell movement 64
Cell viability 48
Cell survival 49
Monocytes I
Systemic autoimmune syndrome 92
Necrosis 136
Engulfment of cells 51
Inflammatory response 62
Quantity of leukocytes 67
Endocytosis 48
Quantity of blood cells 71
Apoptosis 120
Cell movement of phagocytes 49
Quantity of cells 97
Immune response of cells 47
Cell movement 107
Migration of cells 99
Cell viability 79
Cell survival 81
Inflammation of organ 76
Quantity of myeloid cells 42
Cell death of tumor cell lines 79
Inflammation of body cavity 59
Proliferation of immune cells 47
Monocytes II
Necrosis 31
Systemic autoimmune syndrome 22
Inflammation of joint 18
Immune mediated inflammatory disease 20
Leukocyte migration 17
Homeostasis of blood cells 14
Quantity of leukocytes 17
Leukopoiesis 15
Homeostasis of leukocytes 13
Hematopoiesis of mononuclear leukocytes 14
Cell movement of leukocytes 15
Migration of cells 23
Cell death of immune cells 11
Hypersensitive reaction 11
Metabolism of protein 13
Engulfment of cells 9
Cellular homeostasis 18
Quantity of cells 19
Cell survival 17
Expression of RNA 15
B
log2 (NormCounts+1)
Lyn
Hif1a
Lmo4
Csf2rb
Myd88
Cxcr2
Nfkbia
Cebpb
G0 G1 G2 G3 G4 CMoP M1 M2
WT
IL-1rn-KO

Supplementary Figure S6 (related to Figure 5). Single-cell RNA sequencing from CD11b^{+} myeloid cells in bone marrow of C57BL/6J wild-type and IL-1rn knock-out mice. Single-cell RNA sequencing from bone marrow CD11b^{+} myeloid cells from C57BL/6J wild-type (WT, n=1 sample from 2 biologically independent animals) and IL-1rn knockout (IL-1rn-KO, n=1 sample from 2 biologically independent animals) mice. (A) Ingenuity pathway analysis (IPA) of "Diseases and Functions" of gene sets enriched in the clusters G0 to G4 neutrophils, common monocyte progenitors (CMoP I), and type I and II monocytes from IL-1rn-KO versus WT mice. Selected significantly enriched functions are shown with their $-\log_{10}$ (Benjamini-Hochberg (BH) adjusted p value) in x axis, number of genes stated in the bar plots and Z-score as color scale. (B) Expression level ($\log_2$ (NormCounts + 1)) of selected genes for the same clusters. Data are means for violin plots. Statistical analyses were performed with right-tailed Fisher's exact test (A) or two-tailed Wilcoxon Rank Sum test (B), with Benjamini-Hochberg correction for multiple comparisons. Adjusted p values < 0.05 are reported. Complete lists of detected genes are provided as Supplementary Data 2. Source data are provided as a Source Data file.

A

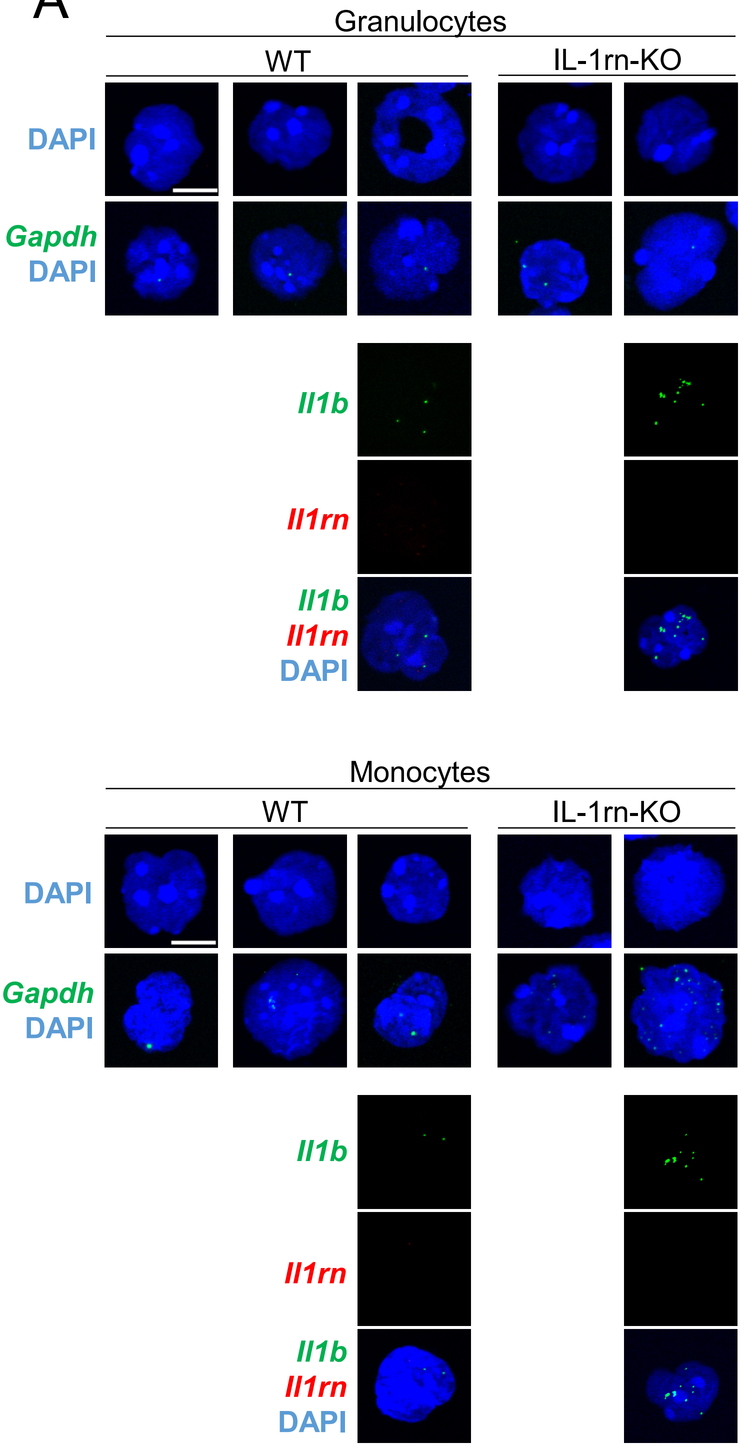
Granulocytes
WT
IL-1rn-KO
DAPI
Gapdh
DAPI
Il1b
Il1rn
Il1b
Il1rn
DAPI
Monocytes
WT
IL-1rn-KO
DAPI
Gapdh
DAPI
Il1b
Il1rn
Il1b
Il1rn
DAPI

Supplementary Figure S7 (related to Figure 5). Identification of the sources of IL-1β and IL-1rn in the bone marrow CD11b$^+$ myeloid compartment at the single cell level by RNA-FISH. (A) RNA fluorescent in situ hybridization (RNA-FISH) in bone marrow CD11b$^+$Gr-1^{hi}f4/80$^-$ granulocytes and CD11b$^+$Gr-1$^+$f4/80$^+$ monocytes from C57BL/6J wild-type (WT) and IL-1rn knockout (IL-1rn-KO) mice (n=4 biologically independent animals per group). Representative images of cells expressing *Il1b* (green) and/or *Il1rn* (red). Nuclei were counterstained with DAPI, no probes were used in negative controls and expression of *Gapdh* (green) was used as positive control. Scale bar, 10μm.

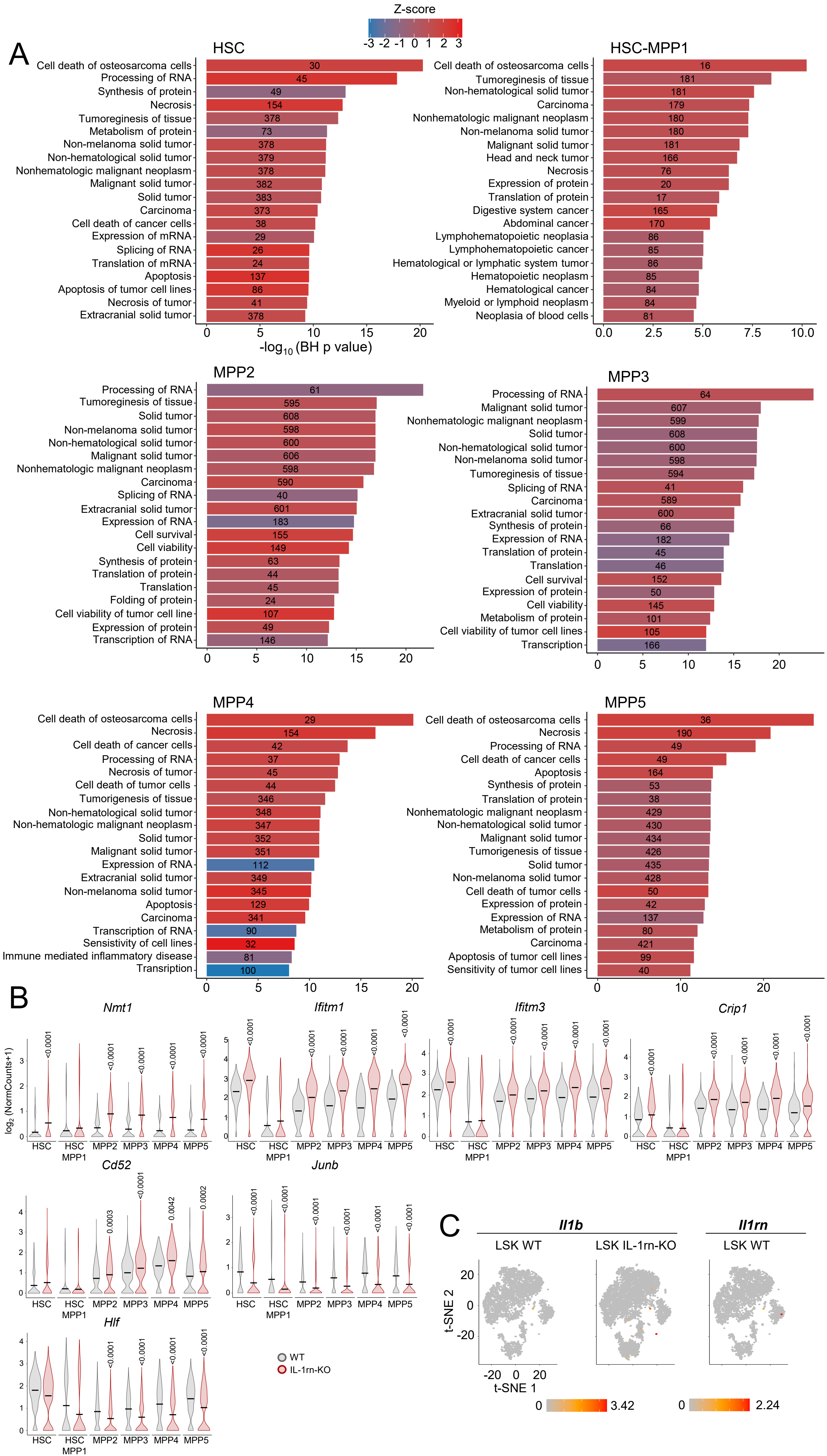
A
B
C
Z-score
HSC
HSC-MPP1
MPP2
MPP3
MPP4
MPP5
-log10 (BH p value)
Cell death of osteosarcoma cells
Processing of RNA
Synthesis of protein
Necrosis
Tumoreginesis of tissue
Metabolism of protein
Non-melanoma solid tumor
Non-hematological solid tumor
Nonhematologic malignant neoplasm
Malignant solid tumor
Solid tumor
Carcinoma
Cell death of cancer cells
Expression of mRNA
Splicing of RNA
Translation of mRNA
Apoptosis
Apoptosis of tumor cell lines
Necrosis of tumor
Extracranial solid tumor
Head and neck tumor
Expression of protein
Translation of protein
Digestive system cancer
Abdominal cancer
Lymphohematopoietic neoplasia
Lymphohematopoietic cancer
Hematological or lymphatic system tumor
Hematopoietic neoplasm
Hematological cancer
Myeloid or lymphoid neoplasm
Neoplasia of blood cells
Expression of RNA
Cell survival
Cell viability
Translation
Folding of protein
Cell viability of tumor cell line
Transcription of RNA
Cell viability of tumor cell lines
Transcription
Cell death of tumor cells
Tumorigenesis of tissue
Non-hematologic malignant neoplasm
Sensistivity of cell lines
Immune mediated inflammatory disease
Transription
Sensitivity of tumor cell lines
Nmt1
Ifitm1
Ifitm3
Crip1
Cd52
Junb
Hlf
log2 (NormCounts+1)
WT
IL-1rn-KO
Il1b
Il1rn
LSK WT
LSK IL-1rn-KO
t-SNE 1
t-SNE 2

Supplementary Figure S8 (related to Figure 6). Single-cell RNA sequencing from hematopoietic stem and progenitor cells in bone marrow of C57BL/6J wild-type and IL-1rn knock-out mice. Single-cell RNA sequencing from bone marrow $Lin^{-}Sca\text{-}1^{+}c\text{-}Kit^{+}$ (LSK) cells from C57BL/6J wild-type (WT, n=1 sample from 2 biologically independent animals) and IL-1rn knockout (IL-1rn-KO, n=1 sample from 2 biologically independent animals) mice. (A) IPA of "Diseases and Functions" of gene sets enriched in the clusters hematopoietic stem cells (HSC), multipotent progenitors (MPP) 1, MPP2, MPP3, MPP4, MPP5 from IL-1rn-KO versus WT mice. Selected significantly enriched functions are shown with their $-\log_{10}$ (BH adjusted p value) in x axis, number of genes stated in the bar plots and Z-score as color scale. (B) Expression level ($\log_2$ (NormCounts + 1)) of selected genes for each cluster of BM LSK from WT and IL-1rn-KO mice. (C) t-SNE plots visualizing expression levels of *Il1b* and *Il1rn*. Color bar, log1p(TP10K). Data are means for violin plots. Statistical analyses were performed with right-tailed Fisher's exact test (A) or two-tailed Wilcoxon Rank Sum test (B), with Benjamini-Hochberg correction for multiple comparisons. Adjusted p values < 0.05 are reported. Complete lists of detected genes are provided as Supplementary Data 3. Source data are provided as a Source Data file.

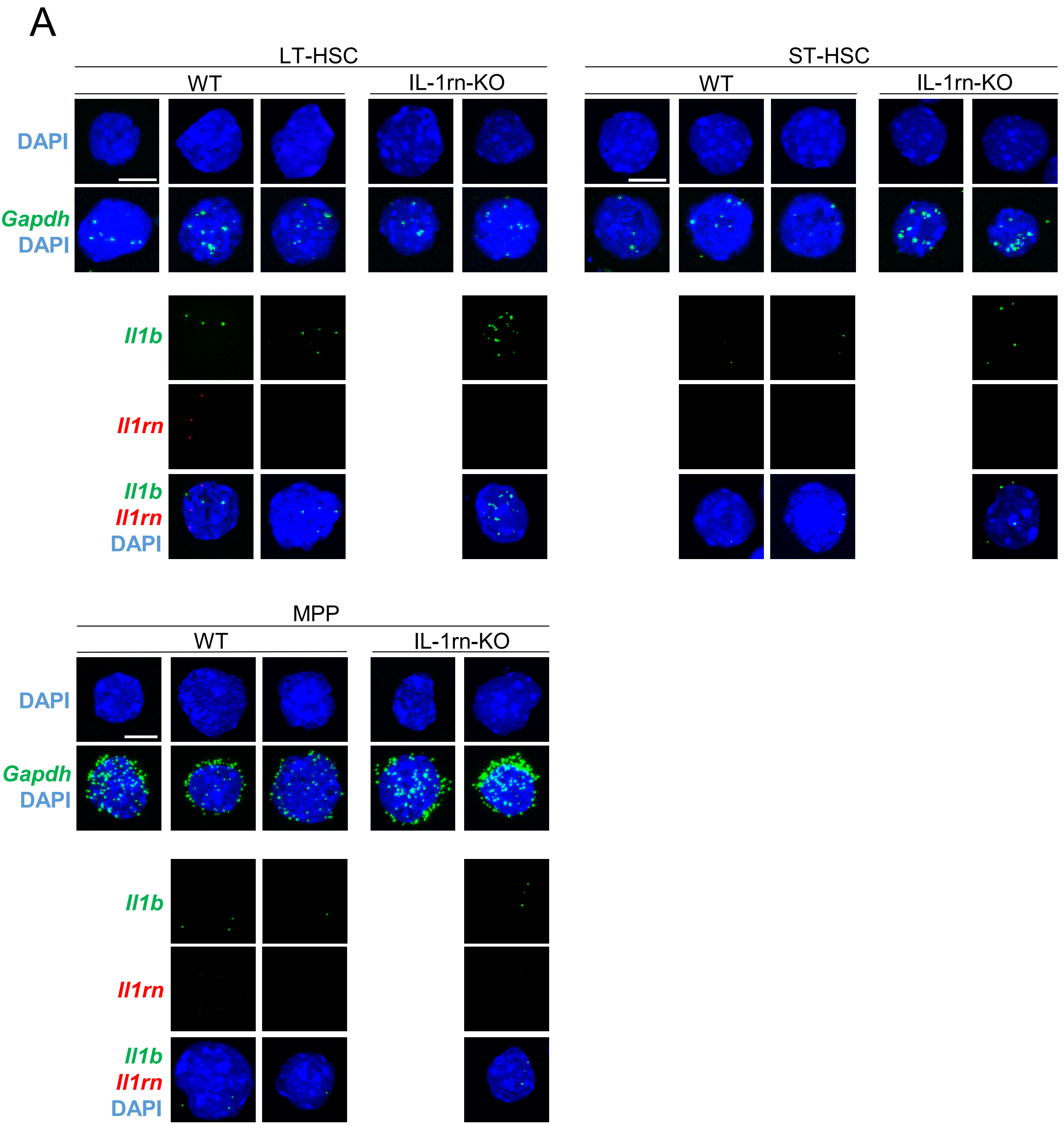
A
LT-HSC
ST-HSC
MPP
WT
IL-1rn-KO
DAPI
Gapdh
DAPI
Il1b
Il1rn
Il1b
Il1rn
DAPI

Supplementary Figure S9 (related to Figure 6). Identification of the sources of IL-1β and IL-1rn in the bone marrow hematopoietic stem and progenitor cell compartment at the single cell level by RNA-FISH. (A) RNA fluorescent in situ hybridization (FISH) in bone marrow $Lin^{-}Sca\text{-}1^{+}c\text{-}Kit^{+}$ (LSK) $CD34^{-}Flt3^{-}$, long-term HSC (LT-HSC); LSK $CD34^{+}Flt3^{-}$, short-term HSC (ST-HSC) and LSK $CD34^{+}Flt3^{+}$, multipotent progenitors (MPP), from C57BL/6J wild-type (WT) and IL-1rn knockout (IL-1rn-KO) mice (n=3 biologically independent animals per group). Representative images of cells expressing *Il1b* (green) and/or *Il1rn* (red). Nuclei were counterstained with DAPI, no probes were used in negative controls and expression of *Gapdh* (green) was used as positive control. Scale bar, 10µm.

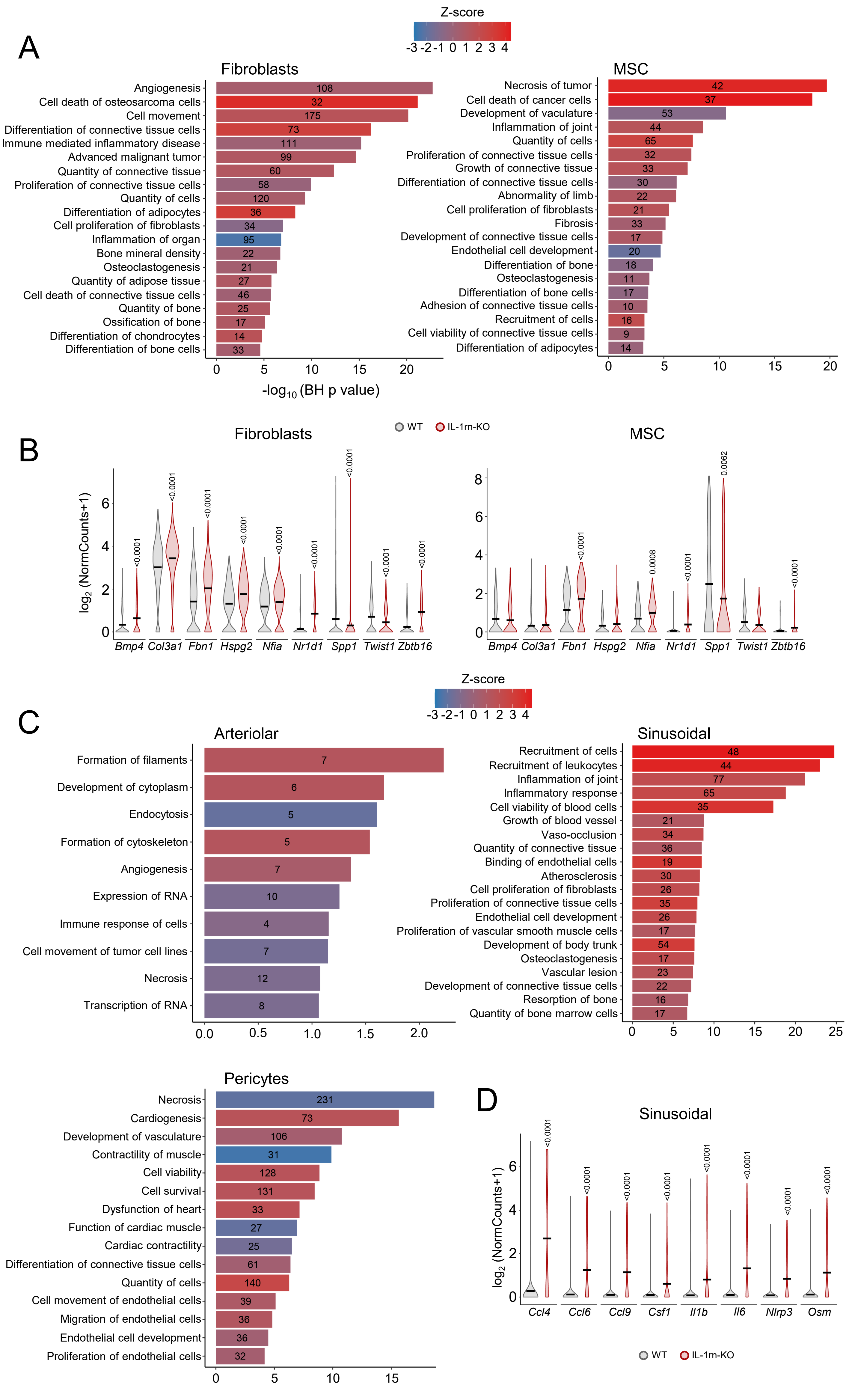

A
Z-score
-3 -2 -1 0 1 2 3 4
Fibroblasts
Angiogenesis 108
Cell death of osteosarcoma cells 32
Cell movement 175
Differentiation of connective tissue cells 73
Immune mediated inflammatory disease 111
Advanced malignant tumor 99
Quantity of connective tissue 60
Proliferation of connective tissue cells 58
Quantity of cells 120
Differentiation of adipocytes 36
Cell proliferation of fibroblasts 34
Inflammation of organ 95
Bone mineral density 22
Osteoclastogenesis 21
Quantity of adipose tissue 27
Cell death of connective tissue cells 46
Quantity of bone 25
Ossification of bone 17
Differentiation of chondrocytes 14
Differentiation of bone cells 33
0 5 10 15 20
-log10 (BH p value)
MSC
Necrosis of tumor 42
Cell death of cancer cells 37
Development of vaculature 53
Inflammation of joint 44
Quantity of cells 65
Proliferation of connective tissue cells 32
Growth of connective tissue 33
Differentiation of connective tissue cells 30
Abnormality of limb 22
Cell proliferation of fibroblasts 21
Fibrosis 33
Development of connective tissue cells 17
Endothelial cell development 20
Differentiation of bone 18
Osteoclastogenesis 11
Differentiation of bone cells 17
Adhesion of connective tissue cells 10
Recruitment of cells 16
Cell viability of connective tissue cells 9
Differentiation of adipocytes 14
0 5 10 15 20
B
WT
IL-1rn-KO
Fibroblasts
log2 (NormCounts+1)
0 2 4 6
<0.0001
Bmp4 Col3a1 Fbn1 Hspg2 Nfia Nr1d1 Spp1 Twist1 Zbtb16
MSC
0 2 4 6 8
<0.0001
0.0008
0.0062
Bmp4 Col3a1 Fbn1 Hspg2 Nfia Nr1d1 Spp1 Twist1 Zbtb16
C
Z-score
-3 -2 -1 0 1 2 3 4
Arteriolar
Formation of filaments 7
Development of cytoplasm 6
Endocytosis 5
Formation of cytoskeleton 5
Angiogenesis 7
Expression of RNA 10
Immune response of cells 4
Cell movement of tumor cell lines 7
Necrosis 12
Transcription of RNA 8
0.0 0.5 1.0 1.5 2.0
Sinusoidal
Recruitment of cells 48
Recruitment of leukocytes 44
Inflammation of joint 77
Inflammatory response 65
Cell viability of blood cells 35
Growth of blood vessel 21
Vaso-occlusion 34
Quantity of connective tissue 36
Binding of endothelial cells 19
Atherosclerosis 30
Cell proliferation of fibroblasts 26
Proliferation of connective tissue cells 35
Endothelial cell development 26
Proliferation of vascular smooth muscle cells 17
Development of body trunk 54
Osteoclastogenesis 17
Vascular lesion 23
Development of connective tissue cells 22
Resorption of bone 16
Quantity of bone marrow cells 17
0 5 10 15 20 25
Pericytes
Necrosis 231
Cardiogenesis 73
Development of vasculature 106
Contractility of muscle 31
Cell viability 128
Cell survival 131
Dysfunction of heart 33
Function of cardiac muscle 27
Cardiac contractility 25
Differentiation of connective tissue cells 61
Quantity of cells 140
Cell movement of endothelial cells 39
Migration of endothelial cells 36
Endothelial cell development 36
Proliferation of endothelial cells 32
0 5 10 15
D
Sinusoidal
log2 (NormCounts+1)
0 2 4 6
<0.0001
Ccl4 Ccl6 Ccl9 Csf1 Il1b Il6 Nlrp3 Osm
WT
IL-1rn-KO

Supplementary Figure S10 (related to Figure 7). Single-cell RNA sequencing from $CD63^+$ stromal cells in bone marrow of C57BL/6J wild-type and IL-1rn knock-out mice. Single-cell RNA sequencing from bone marrow $CD45^-CD31^-Ter119^-CD63^+$ stromal cells from C57BL/6J wild-type (WT, n=1 sample from 2 biologically independent animals) and IL-1rn knockout (IL-1rn-KO, n=1 sample from 2 biologically independent animals) mice. (A) IPA of "Diseases and Functions" of gene sets enriched in the clusters fibroblasts and mesenchymal stromal cells (MSC) from IL-1rn-KO versus WT mice. Selected significantly enriched functions are shown with their $-\log_{10}$ (BH adjusted p value) in x axis, number of genes stated in the bar plots and Z-score as color scale. (B) Expression level ($\log_2$ (NormCounts + 1)) of selected genes for fibroblast and MSC clusters within $CD45^-CD31^-Ter119^-CD63^+$ stromal cells from WT and IL-1rn-KO mice. (C) IPA of "Diseases and Functions" of gene sets enriched in the clusters arteriolar, sinusoidal and pericytes from IL-1rn-KO versus WT mice. Selected significantly enriched functions are shown with their $-\log_{10}$ (BH adjusted p value) in x axis, number of genes stated in the bar plots and Z-score as color scale. (D) Expression level ($\log_2$ (NormCounts + 1)) of selected genes for sinusoidal cluster within $CD45^-CD31^-Ter119^-CD63^+$ stromal cells from WT and IL-1rn-KO mice. Data are means for violin plots. Statistical analyses were performed with right-tailed Fisher's exact test (A, C) or two-tailed Wilcoxon Rank Sum test (B, D), with Benjamini-Hochberg correction for multiple comparisons. Adjusted p values < 0.05 are reported. Complete lists of detected genes are provided as Supplementary Data 4. Source data are provided as a Source Data file.

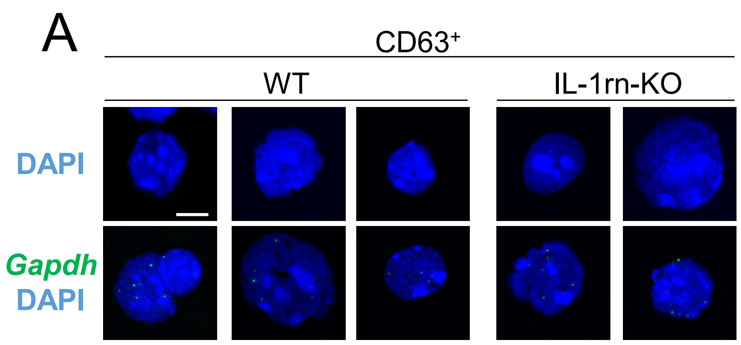
A
CD63+
WT
IL-1rn-KO
DAPI
Gapdh
DAPI

Supplementary Figure S11 (related to Figure 7). Identification of the sources of IL-1β and IL-1rn in the bone marrow CD63^{+} stromal cell compartment at the single cell level by RNA-FISH. (A) RNA fluorescent in situ hybridization (FISH) in bone marrow CD45^{-}CD31^{-}Ter119^{-}CD63^{+} stromal cells from C57BL/6J wild-type (WT) and IL-1rn knockout (IL-1rn-KO) mice (n=4 biologically independent animals per group). Nuclei were counterstained with DAPI, no probes were used in negative controls and expression of *Gapdh* (green) was used as positive control. Scale bar, 10µm.

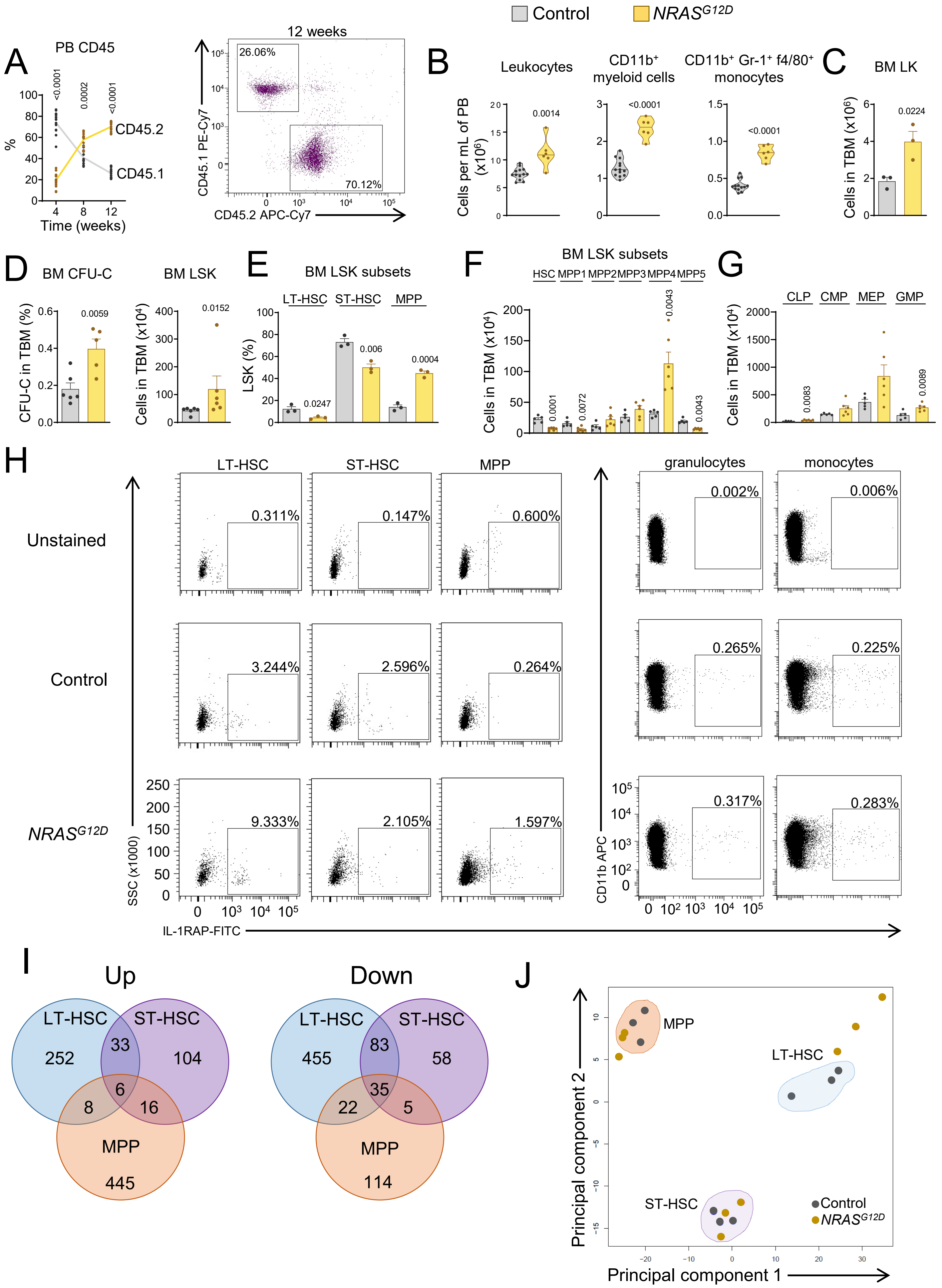

Control
NRASG12D
A
PB CD45
12 weeks
26.06%
70.12%
CD45.2
CD45.1
Time (weeks)
CD45.1 PE-Cy7
CD45.2 APC-Cy7
B
Leukocytes
CD11b+ myeloid cells
CD11b+ Gr-1+ f4/80+ monocytes
Cells per mL of PB (x10^6)
C
BM LK
Cells in TBM (x10^6)
D
BM CFU-C
CFU-C in TBM (%)
BM LSK
Cells in TBM (x10^4)
E
BM LSK subsets
LT-HSC
ST-HSC
MPP
LSK (%)
F
BM LSK subsets
HSC
MPP1
MPP2
MPP3
MPP4
MPP5
Cells in TBM (x10^4)
G
CLP
CMP
MEP
GMP
Cells in TBM (x10^4)
H
LT-HSC
ST-HSC
MPP
granulocytes
monocytes
Unstained
Control
NRASG12D
SSC (x1000)
IL-1RAP-FITC
CD11b APC
I
Up
Down
LT-HSC
ST-HSC
MPP
J
MPP
LT-HSC
ST-HSC
Control
NRASG12D
Principal component 1
Principal component 2

Supplementary Figure S12 (Related to Figure 8). Low IL-1rn is present in a mouse model of pre-leukemic myelopoiesis. (A) Competitive repopulation with $CD45.2^+$ NRAS-$G12D^+$ bone marrow (BM) cells and $CD45.1^+$ B6.SJL healthy cells (1:1) in B6.SJL mice (n=14; 4 weeks n=13). Peripheral blood (PB) chimerism evolution and representative fluorescence-activated cell sorting (FACS) analysis (cells in PB, %). (B-G) Analysis of hematopoiesis in NRAS-$G12D^+$ (*Mx1-Cre*$^+$ *NRAS*^{G12D}) and control (*Mx1-Cre*$^-$ *NRAS*^{G12D}) mice. (B) Number of leukocytes, $CD11b^+$ myeloid cells and $CD11b^+Gr\text{-}1^+f4/80^+$ monocytes in PB (control, n=13; NRAS-$G12D^+$, n=6). (C) Total BM (TBM) number of $Lin^-c\text{-}Kit^+Sca\text{-}1^-$ (LK) progenitors (n=3 per group). (D) BM frequency of colony-forming unit cells (CFU-C) (control, n=6; NRAS-$G12D^+$, n=5) and total number of $Lin^-Sca\text{-}1^+c\text{-}Kit^+$ (LSK) (n=6 per group). (E) Frequencies of LSK subsets within the BM; LSK $CD34^-Flt3^-$ long-term hematopoietic stem cells (LT-HSC), LSK $CD34^+Flt3^-$ short-term HSC (ST-HSC) and LSK $CD34^+Flt3^+$ multipotent progenitors (MPP) (n=3 per group). (F) TBM number of hematopoietic stem and progenitor cell subsets corresponding to HSC (LSK $CD34^-Flt3^-CD48^-CD150^+$), MPP1 (LSK $CD34^+Flt3^-CD48^-CD150^+$), MPP2 (LSK $CD34^+Flt3^-CD48^+CD150^+$), MPP3 (LSK $CD34^+Flt3^-CD48^+CD150^-$), MPP4 (LSK $CD34^+Flt3^+CD48^+CD150^-$) and MPP5 (LSK $CD34^+Flt3^-CD48^-CD150^-$) (control, n=5; NRAS-$G12D^+$, n=6). (G) TBM number of common lymphoid progenitors (CLP, $Lin^-c\text{-}Kit^{low}Sca\text{-}1^{low}CD127^+$), common myeloid progenitors (CMP, LK $CD34^+FcR\gamma^-$), megakaryocyte erythroid progenitors (MEP, LK $CD34^-FcR\gamma^-$) and granulocyte-monocyte progenitors (GMP, LK $CD34^+FcR\gamma^+$) (control, n=5; NRAS-$G12D^+$, n=6). (H) Representative FACS analysis of IL-1 receptor accessory protein (IL-1RAP) staining and unstained negative control in BM LT-HSC, ST-HSC, MPP, $CD11b^+Gr\text{-}1^{hi}f4/80^-$ granulocytes and $CD11b^+Gr\text{-}1^+f4/80^+$ monocytes (cells in each cell subset, %). (I-J) RNA sequencing in BM LT-HSC, ST-HSC and MPP (n=3 per group) from NRAS-$G12D^+$ versus control mice. (I) Venn diagrams show number of up- and down- regulated genes in BM LT-HSC, ST-HSC and MPP from NRAS-$G12D^+$ versus control mice, and overlap of genes. (J) Principal component analysis. Data are biologically independent animals, and means ± S.E.M for bar plots or medians for violin plots, except (I). Statistical analyses were performed with paired two-tailed Student's *t*-test (A), two-tailed Student's *t*-test (B except leukocytes, C, D BM CFU-C, E, F HSC, MPP1, G), or two-tailed Mann-Whitney U test (B leukocytes, D BM LSK, F MPP4, MPP5). p values ≤ 0.05 are reported. Source data are provided as a Source Data file.

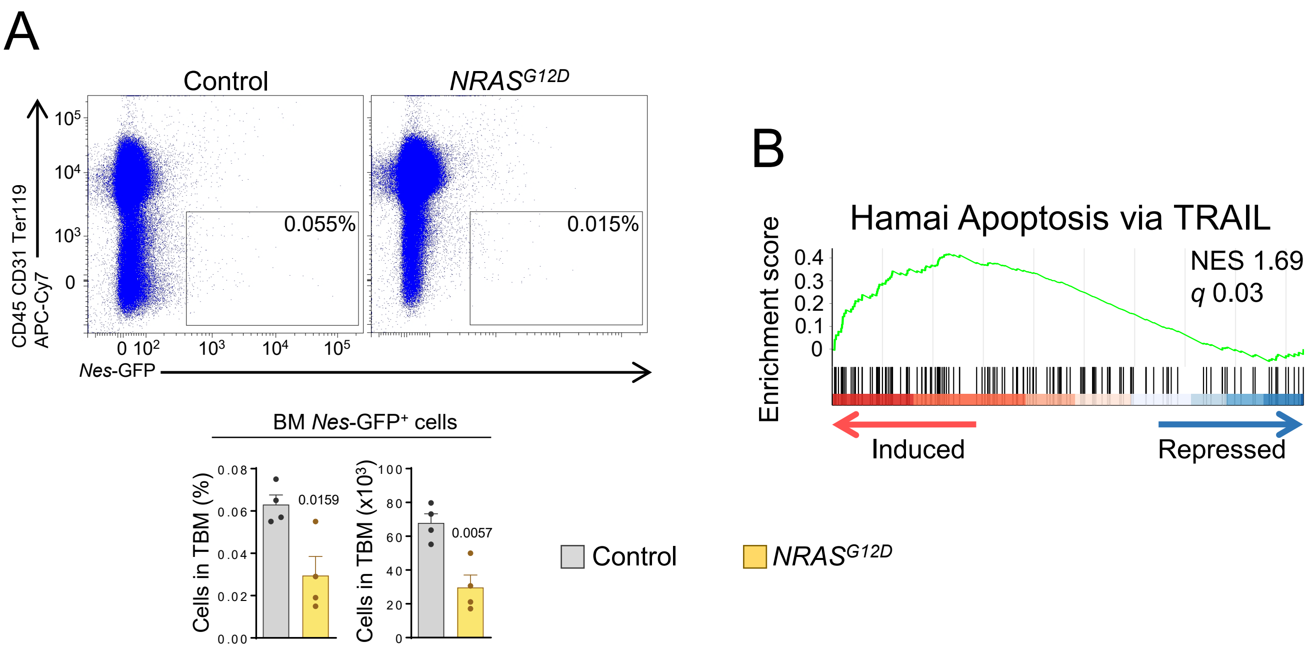
A
Control
NRAS^G12D
CD45 CD31 Ter119 APC-Cy7
Nes-GFP
0.055%
0.015%
BM Nes-GFP+ cells
Cells in TBM (%)
Cells in TBM (x10^3)
0.0159
0.0057
Control
NRAS^G12D
B
Hamai Apoptosis via TRAIL
Enrichment score
NES 1.69
q 0.03
Induced
Repressed

Supplementary Figure 13 (Related to Figure 9). Damage to the bone marrow stroma in a mouse model of pre-leukemic myelopoiesis. *Nes-gfp* mice were used as recipients of bone marrow (BM) nucleated cells from NRAS-G12D^{+} and control mice. (A) Representative fluorescence-activated cell sorting analysis (cells in total BM, TBM, %) and TBM number of *Nes*-GFP^{+} stromal cells (n=4 per group). (B) RNA sequencing in BM *Nes*-GFP^{+} stromal cells (n=2 per group). Enrichment plot of coordinated changes of genes previously reported to be induced or repressed in apoptosis (E-MEXP-247). TRAIL, tumor necrosis factor-related apoptosis inducing ligand. Data are biologically independent animals, and means ± S.E.M (A). Statistical analyses were performed with two-tailed Student's *t*-test (A). p values ≤ 0.05 are reported. Source data are provided as a Source Data file.

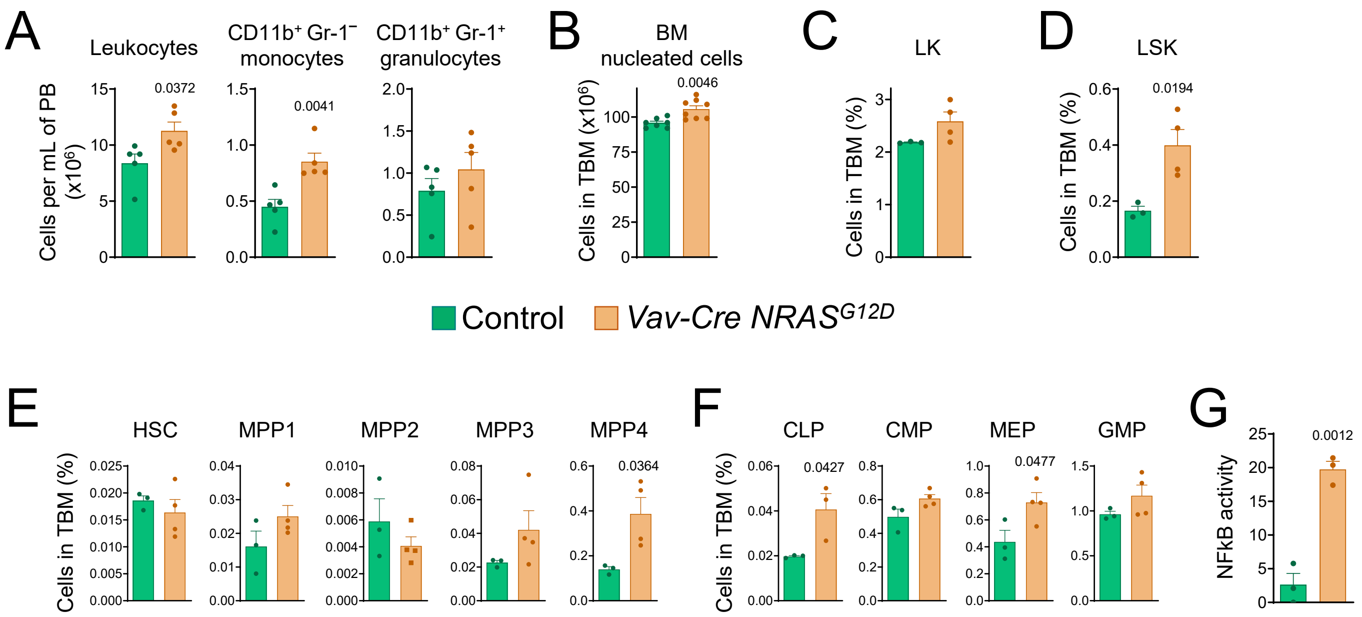
A
Leukocytes
CD11b⁺ Gr-1⁻ monocytes
CD11b⁺ Gr-1⁺ granulocytes
Cells per mL of PB (x10⁶)
0.0372
0.0041
B
BM nucleated cells
Cells in TBM (x10⁶)
0.0046
C
LK
Cells in TBM (%)
D
LSK
Cells in TBM (%)
0.0194
Control
Vav-Cre NRAS G12D
E
HSC
MPP1
MPP2
MPP3
MPP4
Cells in TBM (%)
0.0364
F
CLP
CMP
MEP
GMP
Cells in TBM (%)
0.0427
0.0477
G
NFkB activity
0.0012

Supplementary Figure S14 (Related to Figure 8). Hematopoietic abnormalities and NFκB activation in an alternative model of pre-leukemic myelopoiesis. (A-F) Analysis of hematopoiesis in NRAS-G12D^{+} (*Vav-Cre*$^{+}$ *NRAS*^{G12D}) and control (*Vav-Cre*$^{+}$) mice. (A) Number of leukocytes, CD11b^{+} Gr-1^{-} monocytes and Cd11b^{+}Gr-1^{+} granulocytes in peripheral blood (PB, n=5 per group). (B) Total number of bone marrow (TBM) nucleated cells (control, n=7; NRAS-G12D^{+}, n=8). (C) BM frequency of Lin^{-}c-Kit^{+}Sca-1^{-} (LK) progenitors (control, n=3; NRAS-G12D^{+}, n=4). (D) BM frequency of Lin^{-}Sca-1^{+}c-Kit^{+} (LSK) progenitors (control, n=3; NRAS-G12D^{+}, n=4). (E) BM frequency of hematopoietic stem and progenitor cell subsets corresponding to hematopoietic stem cells (HSC, LSK CD34^{-}Flt3^{-}CD48^{-}CD150^{+}), multipotent progenitors 1 (MPP1, LSK CD34^{+}Flt3^{-}CD48^{-}CD150^{+}), MPP2 (LSK CD34^{+}Flt3^{-}CD48^{+}CD150^{+}), MPP3 (LSK CD34^{+}Flt3^{-}CD48^{+}CD150^{-}) and MPP4 (LSK CD34^{+}Flt3^{+}CD48^{+}CD150^{-}) (control, n=3; NRAS-G12D^{+}, n=4). (F) BM frequency of common lymphoid progenitors (CLP, Lin^{-}c-Kit^{low}Sca-1^{low}CD127^{+}), common myeloid progenitors (CMP, LK CD34^{+}FcRγ^{-}), megakaryocyte erythroid progenitors (MEP, LK CD34^{-}FcRγ^{-}) and granulocyte-monocyte progenitors (GMP, LK CD34^{+}FcRγ^{+}) (control, n=3; NRAS-G12D^{+}, n=4). (G) NFκB transcription factor activity in BM LK progenitors (n=3 per group) calculated based on NFκB target gene expression levels, identified from Synapse ID syn4956655 (Supplementary Data 1), in publicly available RNA sequencing (PRJNA774277). Data are biologically independent animals, and means ± S.E.M. Statistical analyses were performed with two-tailed Student's *t*-test. p values ≤ 0.05 are reported. Source data are provided as a Source Data file.

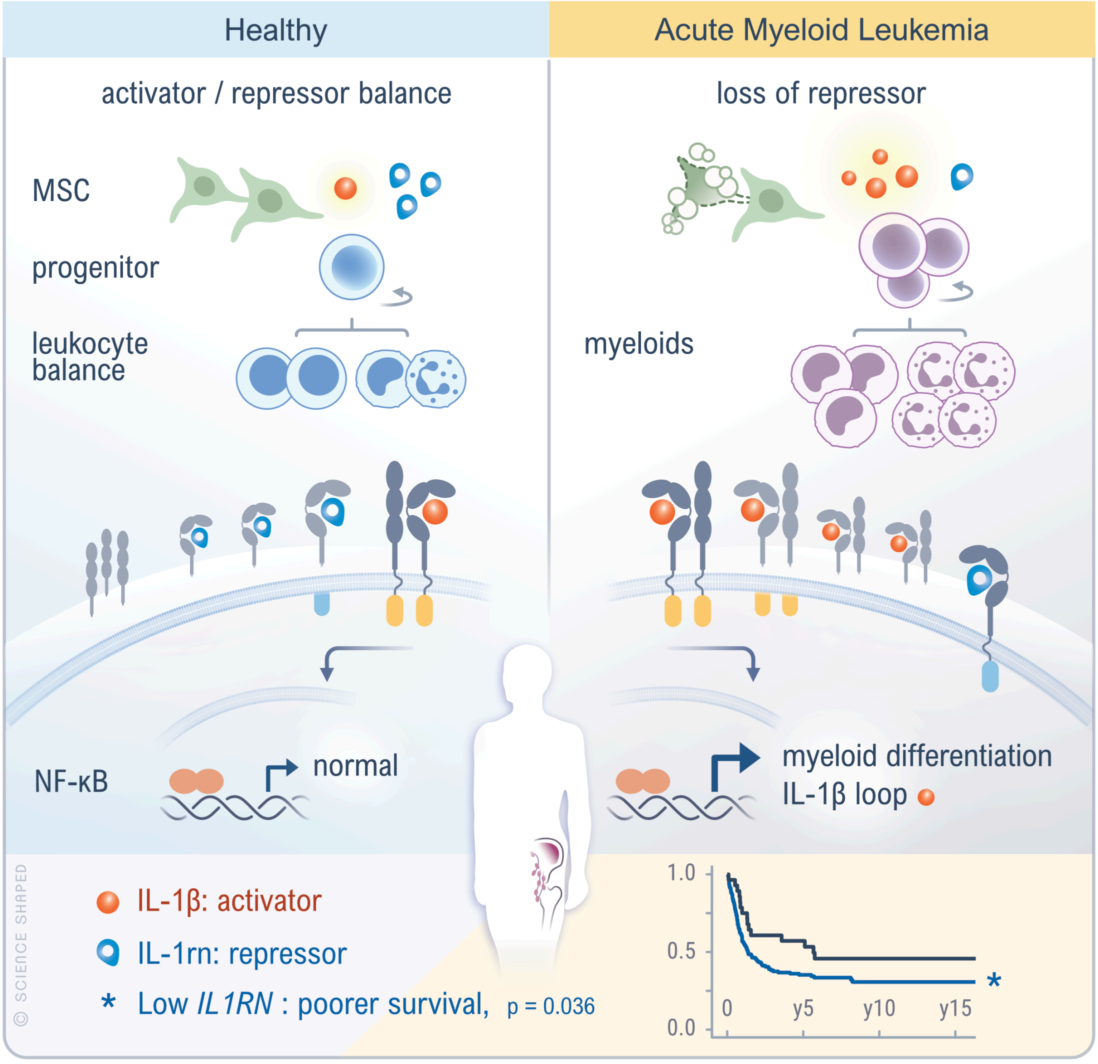
Healthy
Acute Myeloid Leukemia
activator / repressor balance
loss of repressor
MSC
progenitor
leukocyte balance
myeloids
NF-κB
normal
myeloid differentiation
IL-1β loop
IL-1β: activator
IL-1rn: repressor
* Low IL1RN : poorer survival, p = 0.036
1.0
0.5
0.0
0
y5
y10
y15
*
© SCIENCE SHAPED

Supplementary Figure S15. IL-1 receptor antagonist restricts healthy and malignant myeloproliferation. Illustration of the proposed model. Under steady-state, hematopoietic stem and progenitor cell (progenitor) differentiation is controlled by balanced IL-1β/IL-1rn, resulting in low NFκB activation (left). Low IL-1rn contributes to biased IL-1β-induced myelopoiesis dependent on NFκB activation in healthy conditions and pre-leukemic disease, is a prognostic marker for AML patients, and provides a new rationale for IL-1β blockade therapeutic potential (right). MSC, mesenchymal stromal cells.

Supplementary Table S1. Characteristics of human and mice used in this study.

Human cohorts. Figure 1 and associated Supplementary Figure S1.

**Panel**	**Number, sex and age (years)**		
**1a**	Global:<br>188 Low and 16 High, women, aged 43 (19-60),<br>158 Low and 19 High, men, aged 45 (19-61)	M4-M5:<br>80 Low and 14 High, women, aged 43 (22-59),<br>56 Low and 14 High, men, aged 46 (19-60)	
**1b**	M0-M3:<br>114, women, aged 43 (19-60)<br>108, men, aged 43 (19-60)	M4-M5:<br>94, women, aged 43 (19-60)<br>77, men, aged 43 (19-60)	
	Healthy donor	AML	MDS
**1c-d**	19, women, aged 45 (27-65)<br>13, men, aged 42 (23-79)	7, women, aged 68 (52-94)<br>14, men, aged 65 (52-74)	2, men, aged 52 and 68
**1e**	10, women, aged 42 (27-58)<br>9, men, aged 39 (23-79)	5, women, aged 72 (52-84)<br>7, men, aged 64 (52-72)	
**1f**	6, women, aged 40 (27-58)<br>4, men, aged 36 (27-43)	1, woman, aged 52<br>5, men, aged 63 (52-71)	
**1g**	3, women, aged 27 (22-30)<br>2, men, aged 52 and 79	2, women, aged 52 and 73<br>2, men, aged 54 and 79	

**S1A**	M0-M3:<br>114, women, aged 43 (19-60)<br>108, men, aged 43 (19-60)	M4-M5:<br>94, women, aged 43 (19-60)<br>77, men, aged 43 (19-60)
**S1B-C**	7, women, aged 47 (27-67)<br>12, men, aged 48 (18-67)	
	Healthy donor	AML
**S1D**	5, women, aged 42 (27-58)<br>4, men, aged 36 (27-43)	1, woman, aged 52<br>5, men, aged 64 (52-71)
**S1E**	2, women, aged 22 and 29<br>2, men, aged 52 and 79	3, women, aged 64 (52-72)<br>2, men, aged 54 and 79
**S1F**	3, women, aged 27 (22-30)<br>2, men, aged 52 and 79	2, women, aged 52 and 72<br>2, men, aged 54 and 79
**S1G**	5, women, aged 42 (30-48)<br>6, men, aged 40 (28-79)	4, women, aged 74 (52-94)<br>1, man, aged 71

Animal cohorts.

Figure 1 and associated Supplementary Figure S1.

**Panel**	**Sex**	**Strain**	**Donor**	**Age at transplant (weeks)**	**Initiation of treatment (weeks after transplant)**	**Duration of treatment (weeks)**
**1h**	Females	NSG-SGM3	AML9	10-11	4	4
			AML7		7	16
**1i**	Females		AML7	10-11	7	16
**1j**	Males		AML21	7-8	6	4
			AML22			
			AML23			
**1k**	Females		AML3	15-16	6	36

**Panel**	**Sex**	**Strain**	**Donor**	**Age at transplant (weeks)**	**Initiation of treatment (weeks after transplant)**	**Duration of treatment (weeks)**
**S1I**	Males	NSG-SGM3	AML21	7-8	6	4
			AML22			
			AML23			
**S1J**	Females		AML3	15-16	6	36

Figure 2 and associated Supplementary Figure S2 and S3.

			Age (weeks)	
**Panel**	**Sex**	**Strain**	WT	KO
**2a**	Females	Wild-type (WT, C57BL/6J) IL-1rn knockout (KO, C57BL/6J)	10-22	13-33
**2b**	Males		43-55	49-61
**2c**	Females		15-17	14-21
**2d**	Females		10-14	14-21
**2e**	Females		2, aged 11	4, aged 14-18
	Males		7, aged 31	8, aged 36-37
**2f**	Females		10-22	19-33
**2k:** ***Il1b***	Females		14-25	13-35
**2k:** ***Il1r1***			8-25	8-35
**2k:** ***Il1rap***			8-12	8-12
**2l:** ***Il1b, Il1r1***	Females		13-15	13-15
**2l:** ***Il1rap***			8-12	8-12
**2m**	Females		14	13-15

**Panel**	**Sex**	**Strain**	**Age at initiation of treatment (weeks)**	**Duration of treatment (weeks)**
**2g**	Females	IL-1rn knockout (KO, C57BL/6J)	9-16	4
**2h**	Females		9-16	10
**2i**	Males		9-21	16
**2j**	Males		9-21	18

			Age (weeks)	
**Panel**	**Sex**	**Strain**	WT	KO
**S2A**	Females	Wild-type (WT, C57BL/6J) IL-1rn knockout (KO, C57BL/6J)	15-17	14-21
**S2B**	Females		15	16-21
**S2C**	Females		11-14	15-21
**S2D**	Males		43-55	49-61

			Age (weeks)	
**Panel**	**Sex**	**Strain**	WT	KO
**S3A**	Males	Wild-type (WT, C57BL/6J) IL-1rn knockout (KO, C57BL/6J)	43-55	49-61
**S3B**	Females		20-34	19-28
**S3C**	Females		7-22	14-33
**S3D-E**	Females		10-14	14-21
**S3F**	Females		2, aged 11	4, aged 14-18
	Males		7, aged 31	8, aged 36-37
**S3G**	Males		38-48	38-48

Panel	Sex	Strain	Age at initiation of treatment (weeks)	Duration of treatment (weeks)
**S3H**	Females	Wild-type<br>(WT, C57BL/6J)<br>IL-1rn knockout<br>(KO, C57BL/6J)	15-22	0, 4 and 8

Figure 3 and associated Supplementary Figure S4

Panel	Sex	Strain	Age (weeks)	
			WT	KO
**3a-c**	Females	Wild-type (WT, C57BL/6J)<br>IL-1rn knockout (KO, C57BL/6J)	24	24-25

Panel	Sex	Strain	Age at initiation of treatment (weeks)	Duration of treatment (weeks)
**3d**	Females	IL-1rn knockout (KO, C57BL/6J)	18-34	12

Panel	Sex	Strain	Age (weeks)	
			WT	KO
**S4A-E, I-J**	Females	Wild-type (WT, C57BL/6J)<br>IL-1rn knockout (KO, C57BL/6J)	24	24-25
**S4F-H**	Males		44	44

Panel	Sex	Strain	Age (weeks)	
**S4I: GSE165810**	Females and males	Wild-type (C57BL/6)	6-12	20 days of treatment
**S4J: GSE166629**	Not disclosed	Boy/J mice (CD45.1) transplanted with sorted hematopoietic progenitor cells from *HSC-SCL-Cre-ER*^{T} *R26R-EYFP Cebpa*$^{+/+}$ (C57BL/6, CD45.2) and competitor bone marrow from wild-type (C57BL/6, CD45.2)	Not disclosed	20 days of treatment

Figure 4 and associated Supplementary Figure S5

			Age (weeks)	
**Panel**	**Sex**	**Strain**	WT	KO
**4a**	Females	Wild-type (WT, C57BL/6J) IL-1rn knockout (KO, C57BL/6J)	14-17	15-21
**4b**	Females		14-22	14-33
**4c**	Females		31-34	36-38
**4f**	Females		22	22
**4g: *Lepr*, *Vcam1*, *Angpt1***	Males		36	40
**4g: *Cxcl12*, *Adipoq*, *Il1rn***			16-20	14-16

**Panel**	**Sex**	**Strain**	**Age at initiation of treatment (weeks)**	**Duration of treatment (weeks)**
**4d**	Females	IL-1rn knockout (KO, C57BL/6J)	9-16	10
**4e**	Males		9-21	18

			Age (weeks)	
**Panel**	**Sex**	**Strain**	WT	KO
**S5A**	Females	Wild-type (WT, C57BL/6J) IL-1rn knockout (KO, C57BL/6J)	14-17	15-21
**S5B**	Females		14-22	14-33
**S5C**	Females		11-13	15-21
**S5D: GSE108892**	Females and males	*VEcad-Cre R26R-tdTomato* (C57BL/6)<br>*Lepr-Cre R26R-tdTomato* (C57BL/6)<br>*Col2.3-Cre R26R-tdTomato* (C57BL/6)	4-12	

Figure 5-7 and associated Supplementary Figure S6-S11

			Age (weeks)	
**Experiment**	**Sex**	**Strain**	WT	KO
**scRNA-Seq**	Females	Wild-type (WT, C57BL/6J)<br>IL-1rn knockout (KO, C57BL/6J)	13-15	14
**RNA-FISH**	Males		33	34

Figure 8 and associated Supplementary Figure S12

Panel	Sex	Strain	Age (weeks)	Induction time (weeks)	
**8a**	Females	*Mx1-Cre*$^{-}$ *NRAS*^{G12D} (Control, C57BL/6J) *Mx1-Cre NRAS*^{G12D} (*NRAS*^{G12D}, C57BL/6J)	24	20	
**8b**	Females		14-34	10-30	
**8c**	Females		38-45	28-32	
**8d, *Il1b* and *Il1rn***	Males		32-44	12	
**8d, *Il1rap***	Females		24-33	8	
**8e, *Il1b* and *Il1rn***	Males		32-41	11-12	
**8e, *Il1rap***	Females		24-30	8	
**8f**	Males		28-32	18	
**8g**	Males		34	6	
**8h**	Females		59-62	52	8 weeks of treatment

Panel	Sex	Strain	Donor cells	Age at transplant (weeks)	Analysis (weeks after transplant)
**S12A**	Females	Wild-type (B6.SJL, CD45.1)	*Mx1-Cre NRAS*^{G12D} (C57BL/6J, CD45.2) and wild-type (B6.SJL, CD45.1)	6-8	4, 8 and 12

Panel	Sex	Strain	Age (weeks)	Induction time (weeks)
**S12B**	Males	*Mx1-Cre*$^{-}$ *NRAS*^{G12D} (Control, C57BL/6J) *Mx1-Cre NRAS*^{G12D} (*NRAS*^{G12D}, C57BL/6J)	20	6
**S12C-E**	Females		36	20
**S12F-G**	Males		56-58	44
**S12H**	Males		28-32	18
**S12I-J**	Males		34	6

Figure 9 and associated Supplementary Figure S13 and S14

**Panel**	**Sex**	**Strain**	**Age (weeks)**	**Induction time (weeks)**
**9a**	Females	*Mx1-Cre*$^{-}$ *NRAS*^{G12D} (Control, C57BL/6J) *Mx1-Cre NRAS*^{G12D} (*NRAS*^{G12D}, C57BL/6J)	38-45	28-32
**9b**	Males		51-69	39-40
**9c, *Il1b* and *Il1rn***	Females		32-44	10-30
**9c, *Il1rap***			24-33	8

**Panel**	**Sex**	**Strain**	**Donor cells**	**Age at transplant (weeks)**	**Analysis (weeks after transplant)**
**S13A-B**	Males	*Nes-gfp* (C57BL/6J)	*Mx1-Cre*$^{-}$ *NRAS*^{G12D} (Control, C57BL/6J)<br>*Mx1-Cre NRAS*^{G12D} (*NRAS*^{G12D}, C57BL/6J)	28	4

			**Number and age (weeks)**	
**Panel**	**Sex**	**Strain**	Control	*NRAS*^{G12D}
**S14A**	Females	C57BL/6J *Vav-Cre* (Control, C57BL/6J) *Vav-Cre NRAS*^{G12D} (*NRAS*^{G12D}, C57BL/6J)	3, aged 6-7	2, aged 6-7
	Males		2, aged 6-7	4, aged 6-7
**S14B**	Females		4, aged 6-7	4, aged 6-7
	Males		3, aged 6-7	4, aged 6-7
**S14C-F**	Females		2, aged 6-7	2, aged 6-7
	Males		1, aged 6-7	2, aged 6-7
**S14G: PRJNA774277**	Females		1, aged 30-40	1, aged 30-40
	Males		2, aged 30-40	2, aged 30-40

Figure 10

**Panel**	**Sex**	**Strain**	**Donors**	**Age at transplant (weeks)**	**Analysis (weeks after transplant)**
**10a-c**	Females	Wild-type<br>(WT, C57BL/6J)	Wild-type<br>(WT, C57BL/6J) and<br>IL-1rn knockout<br>(KO, C57BL/6J)<br>Wild-type<br>(WT, C57BL/6J) and<br>*Mx1-Cre NRAS*^{G12D}<br>(*NRAS*^{G12D}, C57BL/6J)<br>IL-1rn knockout<br>(KO, C57BL/6J) and<br>*Mx1-Cre NRAS*^{G12D}<br>(*NRAS*^{G12D}, C57BL/6J)	16	32
**10d-f**	Females	Wild-type<br>(WT, C57BL/6J)<br>IL-1rn knockout<br>(KO, C57BL/6J)	*Mx1-Cre*$^{-}$ *NRAS*^{G12D}<br>(Control, C57BL/6J)<br>*Mx1-Cre NRAS*^{G12D}<br>(*NRAS*^{G12D}, C57BL/6J)	9-20	4-8

**Panel**	**Sex**	**Strain**	**Age at induction (weeks)**	**Initiation of treatment (weeks after induction)**	**Analysis (weeks after initiation of treatment)**
**10g-i**	Males	*Mx1-Cre NRAS*^{G12D}<br>(*NRAS*^{G12D}, C57BL/6J)	12-13	2	4

Supplementary Table 2: List of reagents used in this article

Antibodies			
**Product**	**SOURCE**	**IDENTIFIER**	
**Flow cytometry**			**Dilution**
Alexa Fluor 647 rat anti-mouse CD11b	BD Biosciences	Cat# 557686; RRID AB_396796; clone M1/70	1/100
PE rat anti-mouse Ly-6G/Ly-6C (Gr-1)	BD Biosciences	Cat# 553128; RRID AB_394644; clone RB6-8C5	1/100
FITC rat anti-mouse CD45R/B220	BD Biosciences	Cat# 553088; RRID AB_394618; clone RA3-6B2	1/100
PE-Cy5 hamster anti-mouse CD3e	BD Biosciences	Cat# 553065; RRID AB_394598; clone 145-2C11	1/100
PE rat anti-mouse Ly-6G	BD Biosciences	Cat# 551461; RRID AB_394208; clone 1A8	1/100
PE rat anti-mouse Ly6A/E (Sca-1)	BD Biosciences	Cat# 553336; RRID AB_394792; clone E13-161.7	1/100
FITC rat anti-mouse CD34	BD Biosciences	Cat# 553733; RRID AB_395017; clone RAM34	1/100 and 2/100
Alexa Fluor 647 rat anti-mouse CD34	BD Biosciences	Cat# 560230; RRID AB_1645199; clone RAM34	1/100
APC rat anti-mouse CD135 (Flt3)	BD Biosciences	Cat# 560718; RRID AB_1727425; clone A2F10.1	1/100
PE-Cy7 rat anti-mouse CD117 (c-Kit)	BD Biosciences	Cat# 558163; RRID AB_647250; clone 2B8	1/100
PE rat anti-mouse CD63	BD Biosciences	Cat# 564222; RRID AB_2738678; clone NVG-2	1/100
PE rat anti-mouse CD106 (VCAM)	BD Biosciences	Cat# 561613; RRID AB_10897990; clone 429(MVCAM.A)	1/100
Alexa Fluor 647 rat anti-mouse CD121a (IL-1r1)	BD Biosciences	Cat# 563629; RRID AB_2738332; clone 35F5	1/100
PE-Cy7 rat anti-mouse CD105 (Endoglin)	BioLegend	Cat# 120410; RRID AB_1027700; clone MJ7/18	1/100
Alexa Fluor 488 rat anti-mouse F4/80	eBioscience Thermo Fisher	Cat# 53-4801-82; RRID AB_469915; clone BM8	1/100
Biotin rat anti-mouse CD31	BD Biosciences	Cat# 553371; RRID AB_394817; clone MEC13.3	1/100
Biotin rat anti-mouse TER-119/Erythroid cells	BD Biosciences	Cat# 553672; RRID AB_394985; clone TER-119	1/100
Biotin mouse anti-mouse CD45.2	BD Biosciences	Cat# 553771; RRID AB_395040; clone 104	1/100
BB700 rat anti-mouse Ly6A/E (Sca-1)	BD Biosciences	Cat# 742089, RRID AB_2871369, clone D7	1/100
PE rat anti-mouse CD135 (Flt3)	BD Biosciences	Cat# 553842, RRID AB_395079, clone A2F10.1	1/100
PE rat anti-mouse CD16/32 (FcRγ)	BD Biosciences	Cat# 567020, RRID AB_2870010, clone Ab93	1/100

PE-Cy5 rat anti-mouse CD150 (SLAM)	BioLegend	Cat# 115912, RRID AB_493598, clone TC15-12F12.2	2/150
APC hamster anti-mouse CD48	BD Biosciences	Cat# 562746, RRID AB_2737765, clone HM48-1	1/100 and 2/100
APC rat anti-mouse CD127 (IL-7R)	BD Biosciences	Cat# 564175, RRID AB_2732843; clone SB/199	2/100
FITC rabbit anti-mouse IL-1rap	ProteoGenix	Clone R5P1-A1; Customized; Batch #15893-113021-A01	15/100
Biotin Mouse Lineage Depletion Cocktail	BD Biosciences	Cat# 51-9000794	5/100
Biotin rat anti-mouse CD45R (B220)	eBioscience Thermo Fisher	Cat# 13-0452-82; RRID AB_466449; clone RA3-6B2	0.25µg/150µl
Biotin rat anti-mouse CD19	eBioscience Thermo Fisher	Cat# 13-0193-82; RRID AB_657656; clone eBio1D3 (1D3)	0.25µg/150µl
Biotin rat anti-mouse CD4	eBioscience Thermo Fisher	Cat# 13-0041-82; RRID AB_466325; clone GK1.5	0.25µg/150µl
Biotin rat anti-mouse CD8a	eBioscience Thermo Fisher	Cat# 13-0081-82; RRID AB_466346; clone 53-6.7	0.25µg/150µl
Biotin armenian hamster anti-mouse CD3e	eBioscience Thermo Fisher	Cat# 13-0031-82; RRID AB_466319; clone 145-2C11	0.25µg/150µl
Biotin rat anti-mouse IgM	eBioscience Thermo Fisher	Cat# 13-5790-82; RRID AB_466675; clone II/41	0.25µg/150µl
Biotin rat anti-mouse Ly-6G/Ly-6C (Gr-1)	eBioscience Thermo Fisher	Cat# 13-5931-82; RRID AB_466800; clone RB6-8C5	0.25µg/150µl
Biotin rat anti-mouse TER-119	eBioscience Thermo Fisher	Cat# 13-5921-82, RRID AB_466797, clone TER-119	0.25µg/150µl
FITC rat anti-mouse CD11b	eBioscience Thermo Fisher	Cat# 11-0112-41; RRID AB_11042156; clone M1/70	0.25µg/150µl
PE rat anti-mouse Ly-6G/Ly-6C (Gr-1)	eBioscience Thermo Fisher	Cat# 12-5931-82; RRID AB_466045; clone RB6-8C5	0.25µg/150µl
APC rat anti-mouse CD127	eBioscience Thermo Fisher	Cat# 17-1271-82; RRID AB_469435; clone A7R34	0.25µg/150µl
PerCP-Cy5.5 rat anti-mouse Ly-6A/E (Sca-1)	eBioscience Thermo Fisher	Cat# 45-5981-82; RRID AB_914372; clone D7	0.25µg/150µl
FITC rat anti-mouse CD34	eBioscience Thermo Fisher	Cat# 11-0341-82; RRID AB_465021; clone RAM34	0.25µg/150µl
PE-Cy7 rat anti-mouse CD117 (c-Kit)	eBioscience Thermo Fisher	Cat# 25-1171-82; RRID AB_469644; clone 2B8	0.25µg/150µl
APC rat anti-mouse CD16/CD32 (FcγRII/III)	eBioscience Thermo Fisher	Cat# 17-0161-82; RRID AB_469356; clone 93	0.25µg/150µl
APC rat anti-mouse CD135 (Flt3)	eBioscience Thermo Fisher	Cat# 17-1351-82; RRID AB_10717261; clone A2F10	0.25µg/150µl
APC-eFluor 780 Armenian hamster anti-mouse CD48	eBioscience Thermo Fisher	Cat# 47-0481-82; RRID AB_2573962; clone HM48-1	0.25µg/150µl
PE Rat anti-mouse CD150 (SLAM)	BioLegend	Cat# 115904; RRID AB_313683; clone TC15-12F12.2	1/100

PE-Cy7 mouse anti-mouse CD45.1	BD Biosciences	Cat# 560578; RRID: AB_1727488; clone A20	1/100
FITC rat anti-mouse CD45	BD Biosciences	Cat# 553080; RRID: AB_394610; clone 30-F11	1/100
PE-Cy7 rat anti-mouse F4/80	BioLegend	Cat# 123113; RRID AB_893490; clone BM8	1/100
FITC rat anti-mouse Ly-6A/E (Sca-1)	eBioscience Thermo Fisher	Cat# 11-5981-82, RRID AB_465333; clone D7	1/100
V450 rat anti-mouse CD45	BD Biosciences	Cat# 560501, RRID AB_1645275; clone 30-F11	1/100
Pacific Blue rat anti-mouse TER-119	BioLegend	Cat# 116231, RRID AB_2149212; clone TER-119	1/100
FITC rat anti-mouse CD31 (PECAM-1)	Invitrogen Thermo Fisher	Cat# 11-0311-85, RRID AB_465013; clone 390	1/100
PE-Cy7 rat anti-mouse CD63	BioLegend	Cat# 143910, RRID AB_2565500; clone NVG-2	1/100
FITC mouse anti-human CD45	BD Biosciences	Cat# 345808; RRID AB_2732010; clone 2D1	15/150 and 20/150
PE mouse anti-human CD11b	BD Biosciences	Cat# 557321; RRID AB_396636; clone ICRF44	10/150
PE mouse anti-human CD33	BD Biosciences	Cat# 555450; RRID AB 395843; clone WM53	15/150
FITC mouse anti-human CD34	BD Biosciences	Cat# 560942; RRID AB 10562559; clone 581	15/150
Alexa Fluor 488 anti-human NFκB p50	Luminex Corporation	Cat# 4700-1674	5/100
BV421 mouse anti-human IL-1RAcP (IL-1R3)	BD Biosciences	Cat# 748107; RRID AB_2872568; clone 89412	10/100
BV421 mouse IgG1, κ Isotype Control	BD Biosciences	Cat# 562438; RRID AB_11207319; clone X40	10/100
Alexa Fluor 647 Mouse anti-human NFκB p65 (pS529)	BD Biosciences	Cat# 558422, RRID AB_647136, clone K10-895.12.50	20/100
Alexa Fluor 647 Mouse IgG2b, κ Isotype Control	BD Biosciences	Cat# 558713, RRID AB_1645618, clone 27-35	20/100
Pacific Blue conjugated Annexin V antibody	Invitrogen Thermo Fisher	Cat# A35122	5/100
APC mouse anti-BrdU	BD Biosciences	Cat# 552598; RRID AB_2861367; clone B44	2/100
**Immunofluorescence**			**Dilution**
Goat polyclonal anti-mouse IL-1r1	R&D system	Cat# AF771; RRID AB_355587	2/100
Donkey polyclonal anti-goat IgG H&L (Cy3)	Abcam	Cat# ab6949; RRID AB_955018	1/200

In vivo treatments		
**PRODUCT**	**SOURCE**	**IDENTIFIER**
Rat anti-mouse IL-1β monoclonal	Novus Biologicals	Cat# NB600-1379; RRID AB_2124612; clone 1400.24.17
Mouse anti-mouse IL-1β monoclonal antibody	Invitrogen Thermo Fisher	Cat# MM425B; RRID AB_223529; clone 1400.24.17
Mouse IgG1 kappa isotype control	Invitrogen Thermo Fisher	Cat# 14-4714-82; RRID AB_470111; clone P3.6.2.8.1
InVivoMab hamster anti-mouse IL-1α	BioXCell	Cat# BE0243; RRID AB_2687724; clone ALF-161
InVivoMAb armenian hamster IgG isotype control	BioXCell	Cat# BE0091; RRID AB_1107773
Anti-human IL-1β therapeutic antibody (FDA-approved ILARIS® - Canakinumab)	Novartis	European Agency Number EMEA/H/C/001109
Anakinra (Kineret®)	Swedish Orphan Biovitrum (Sobi)	European Agency Numbe EMEA/H/C/000363
Bortezomib	Thermo Scientific Thermo Fisher	Cat# J60378.MB
Human IL-1β	Peprotech	Cat# 200-01B

**Cytokine measurements**		
**PRODUCT**	**SOURCE**	**IDENTIFIER**
Bio-Plex Pro Human Cytokine IL-1β Set	Bio-Rad	Cat# 171B5001M
Bio-Plex Pro Human Cytokine IL-1RA Set	Bio-Rad	Cat# 171B5002M
Bio-Plex Pro Mouse Cytokine IL-1β Set	Bio-Rad	Cat# 171G5002M
Bio-Plex Pro Mouse Cytokine IL-6 Set	Bio-Rad	Cat #171G5007M
Bio-Plex Pro Mouse Cytokine IL-10 Set	Bio-Rad	Cat# 171G5009M
Bio-Plex Pro Mouse Cytokine IFN-γ Set	Bio-Rad	Cat# 171G5017M
Bio-Plex Pro Mouse Cytokine TNF-α Set	Bio-Rad	Cat# 171G5023M
Mouse IL-1RA AimPlex bead-based immunoassay	Aimplex Biosciences	Cat# PN: B211239

Fluorophores and Fluorophore-conjugated products		
**PRODUCT**	**SOURCE**	**IDENTIFIER**
**Flow cytometry**		
Amnis® NFκB Translocation Kit	Luminex Corporation	Cat# ACS10000
7-Amino-Actinomycin D (7-AAD)	Luminex Corporation	Cat# 4000-0290
APC-Cy7-conjugated streptavidin	BD Biosciences	Cat# 554063; RRID AB_10054651
PE-TR conjugated streptavidin	Invitrogen Thermo Fisher	Cat# SA1017
PE-conjugated streptavidin	eBioscience Thermo Fisher	Cat# 12-4317-87
Streptavidin Particles Plus - DM	BD Biosciences	Cat# 51-9000810
4’,6-diamidino-2-phenylindole (DAPI)	Sigma-Aldrich Merck	Cat# D8417
Deep Red Anthraquinone 7 (DRAQ7)	BD Biosciences	Cat# 564904; RRID AB_2869621
7-Amino-Actinomycin D (7-AAD)	Invitrogen Thermo Fisher	Cat# A1310
**Immunofluorescence**		
Cy3-Streptavidin conjugate (ZyMAX™ grade)	Invitrogen Thermo Fisher	Cat# 438315

RNA fluorescent *in situ* hybridization (FISH)		
**PRODUCT**	**SOURCE**	**IDENTIFIER**
Mouse *Gapdh* probe type 4	Invitrogen Thermo Fisher	Cat# VB4-10414
Mouse *Il1b* probe type 4	Invitrogen Thermo Fisher	Cat# VB4-18866
Mouse *Il1rn* probe type 1	Invitrogen Thermo Fisher	Cat# VB1-3028457
Mouse *Il1rn* probe type 6	Invitrogen Thermo Fisher	Cat# VB6-3198573
ViewRNA™ ISH Cell Assay Kit	Invitrogen Thermo Fisher	Cat# QVC0001
Preamplifier Mix	Invitrogen Thermo Fisher	Cat# QVC0001 (18818)
Amplifier Mix	Invitrogen Thermo Fisher	Cat# QVC0001 (18819)
Label probe Mix	Invitrogen Thermo Fisher	Cat# QVC0001 (18820)
4', 6-Diamidino-2-Phenylindole (DAPI)	Invitrogen Thermo Fisher	Cat# QVC0001 (18821)
Detergent Solution QC	Invitrogen Thermo Fisher	Cat# QVC0509
Wash buffer components 1 and 2	Invitrogen Thermo Fisher	Cat# QG0507 (19885)

**Primers**		
**GENE**	**FORWARD**	**REVERSE**
**Mouse qRT-PCR primers**		
Il1b	GAAATGCCACCTTTTGACAGTG	TGGATGCTCTCATCAGGACAG
Il1r1	TTACCCGAGGTCCAGTGGTA	CCCCCGGAACGTATAGGACA
Il1rap	TCCTCAATGACACGGGCAAT	GGGAAATGCAACTTTGCTGC
Il1rn	GAGAAACAACCAGCTCATTGC	GGATGCCCAAGAACACACTATG
Cxcl12	TGCATCAGTGACGGTAAACCA	TTCTTCAGCCGTGCAACAATC
Adipoq	TGTTCCTCTTAATCCTGCCCA	CCAACCTGCACAAGTTCCCTT
Axl	ATCACAGGTGCCAGAGGACT	CTGTCCATCTCGAAGCCACA
Stat3	CATTGACCTGCCGATGTCCC	TCAAACGTGAGCGACTCAAAC
Tlr1	CAGTTGGTGAAGAACTCAGGC	CCACATGGGTATAGGACGTTT
Spi1	CCCTCCATCGGATGACTTG	ACATGGTGTGCGGAGAAATC
Csf2rb	GGGCCAGTGTCTACACCCG	GTAAGCCATCTTCTGAGTTTCCCAATGC
Nes	GTCAGATCGCTCAGATCCT	GTGTCTGCAAGCGAGAGTTC
Vcam1	TGGAGGTCTACTCATTCCCTGA	GACAGGTCTCCCATGCACAA
Angpt1	CTCGTCAGACATTCATCATCCAG	CACCTTCTTTAGTGCAAAGGCT
Lepr	AGCTGCACTTAACCTGGCAT	AAAGCCGAGGCATTGTTTGG
B2m	ACCGTCTACTGGGATCGAGA	TGCTATTTCTTTCTGCGTGCAT
Gapdh	CTCCCACTCTTCCACCTTCG	GCCTCTCTTGCTCAGTGTCC
**Human qRT-PCR primers**		
IL1B	CTGTCCTGCGTGTTGAAAGA	TTGGGTAATTTTTGGGATCTACA
ILRN	TGCAAGCCTTCAGAATCTGGG	TCCTTGCAAGTATCCAGCAACT
CASP1	ACCAACTACAGAAGAGTTTGAGGA	ACATTATCTGGTGTGGAAGAGC
IRAK1	TGTGCCGCTTCTACAAAGTGA	ACGATCAGGGCGGCGAAC
IL1RAP	TAGAAGCCTCACTCTTGCCCC	TGGATTTGCCTCATGGTGTCTA
B2M	AGTATGCCTGCCGTGTGAAC	TTCAAACCTCCATGATGCTGCT

Histology		
**PRODUCT**	**SOURCE**	**IDENTIFIER**
Parafinne	VWR	Cat# 1116099025
Hematoxylin	Sigma-Aldrich Merck	Cat# H3136
Eosin	Sigma-Aldrich Merck	Cat# HT110316
Harris solution	Sigma-Aldrich Merck	Cat# HHS80
DPX MOUNTANT	Casa Alvarez	Cat# 10-8500
Bouin's solution	Sigma-Aldrich Merck	Cat# HT 10132-L
Ponceau BS (Biebrich Scarlet)	Sigma-Aldrich Merck	Cat# B6008
Phosphomolybdic acid hydrate 80%	Acros Organic	Cat# 206380250
Phosphotungstic acid hydrate	Sigma-Aldrich Merck	Cat# P4006
Ammonium solution	Sigma-Aldrich Merck	Cat# 105432
Fast Green FCF	Sigma-Aldrich Merck	Cat# F7252
Acetic acid	Riedel-de Haen	Cat# 27221
Xylene	VWR	Cat# 28973363
Potassium permanganate	Sigma-Aldrich Merck	Cat# 399124
Oxalic acid	Sigma-Aldrich Merck	Cat# 194131
Iron alum	Sigma-Aldrich Merck	Cat# 221260
Gold chloride	Sigma-Aldrich Merck	Cat# HT 1004
Sodium Thiosulfate	Sigma-Aldrich Merck	Cat# 72049
Iron (III) chloride	Sigma-Aldrich Merck	Cat# 157740
Silver nitrate	Sigma-Aldrich Merck	Cat# S6506
Sodium hydroxide pellets (NaOH)	Sigma-Aldrich Merck	Cat# 1064821000
Formaldehyde solution 4% buffered (Titripack)	VWR	Cat# 11699408
Ethanol absolute	VWR	Cat# 3001918

IL-1RAP antibody generation		
**PRODUCT**	**SOURCE**	**IDENTIFIER**
Tween 20	Sigma-Aldrich Merck	Cat# P2287
Tris hydrochloride	Sigma-Aldrich Merck	Cat# RES3098T-B7
Glycine	Sigma-Aldrich Merck	Cat# G7126
PEG	Sigma-Aldrich Merck	Cat# 181986
PBS	Gibco Thermo Fisher	Cat# 10010023
Pierce™ Protein-Free (PBS) Blocking Buffer	Thermo Scientific Thermo Fischer	Cat# 37572
Rabbit LiAb-SFRab™ phage library	ProteoGenix	Cat# LiAb-SFRabTM
E Coli TG1	Lucigen	Cat# 60502-1
M13KO7 Helper phage	NEB	Cat# N0315S
Anti-M13 HRP-conjugated mouse monoclonal antibody	ProteoGenix	Cat# ATX-PDAB-HRP
Coomassie blue staining	Sigma-Aldrich Merck	Cat# 6104-58-1
tetramethyl benzidine substrate	ProteoGenix	Cat# PTX-TMB
pXten1 Expression Vector	ProteoGenix	Cat# PX-XTE-002
XtenCHO™ Expression Medium	ProteoGenix	Cat# PX-XTE-003
Antibody Expression Positive Control Vector	ProteoGenix	Cat# PX-XTE-004
XtenFect Reagent, Stock solution	ProteoGenix	Cat# PX-XTE-005
XtenFect Reagent, Working solution	ProteoGenix	Cat# PX-XTE-006
XtenCHO™ Enhancer	ProteoGenix	Cat# PX-XTE-007
FITC conjugation kit	Sigma-Aldrich Merck	Cat# FITC1-1KT
Protein A purification kit	Thermo Scientific Thermo Fisher	Cat# 44667
Extracellular portion of mouse IL-1RAP protein	Sinobiological	Cat# 52657-M08H

Eukaryotic cell lines		
**PRODUCT**	**SOURCE**	**IDENTIFIER**
THP-1 cells	DSMZ	Cat# ACC 16
XtenCHO™ cells	ProteoGenix	Cat# PX-XTE-001

Other reagents		
**PRODUCT**	**SOURCE**	**IDENTIFIER**
Lympholyte®-H Cell Separation Media	Cedarlane	Cat# CL5015
EasySep™ Human CD34 Positive Selection Kit II	Stem Cell Technologies	Cat# 15086
StemSpan Serum-free Expansion Medium (SFEM)	Stem Cell Technologies	Cat# 9650
MethoCult™ GF M3434	Stem Cell Technologies	Cat# 03434
Ammonium Chloride Solution	Stem Cell Technologies	Cat# 07850
Ammonium chloride (NH_4Cl)	Sigma-Aldrich Merck	Cat# A9434
Collagenase Type I	Stem Cell Technologies	Cat#7902
RPMI 1640 Medium	Gibco Thermo Fisher	Cat# 11835030
Murine thrombopoietin	Peprotech	Cat# 315-14
Recombinant murine stem cell factor	Peprotech	Cat# 250-03
Murine Flt3-Ligand	Peprotech	Cat# 250-31L
Penicilin-Streptomycin-Glutamine	Gibco Thermo Fisher	Cat# 10378016
Pierce™ 16% Formaldehyde (w/v), Methanol-free	Thermo Scientific Thermo Fisher	Cat# 28908
Methanol	VWR International	Cat# 20847.318
Tri Reagent®	Sigma-Aldrich Merck	Cat# T9424
Dynabeads™ mRNA DIRECT™ Purification Kit	Invitrogen Thermo Fisher	Cat# 61012
PicoPure™ RNA Isolation Kit	Applied Biosystems Thermo Fischer	Cat# KIT0204
Sucrose	Sigma-Aldrich Merck	Cat# 84097
Tissue-Tek® OCT™	Sakura Finetek	Cat# 4583
High capacity reverse transcription kit	Applied Biosystems Thermo Fisher	Cat# 4368814
qPCR Human Reference Total RNA	Clontech TaKaRa Bio	Cat# 636690
Mouse Universal Reference Total RNA	Clontech TaKaRa Bio	Cat# 636657
SYBR green PCR master mix	Applied Biosystems ThermoFisher	Cat# 4309155
RNA-ase DNA-ase free water	Cytiva Life Sciences	Cat# SH30538.02
Heat Inactivated Fetal Bovine Serum (FBS)	Gibco Thermo Fisher	Cat# 10082147
PBS Tablets	Gibco Thermo Fisher	Cat# 18912-014
Bovine serum albumin (BSA)	Sigma-Aldrich Merck	Cat# A7906
poly-inosine:poly-cytosine (polyI:polyC)	Sigma-Aldrich Merck	Cat# P9582
Lypopolysaccharides from E.Coli	Sigma-Aldrich Merck	Cat# L4391
Busulfan (Busilvex®)	Pierre Fabre Pharmaceuticals	EMEA/H/C/000472; EU/1/03/254/002
Dimethyl sulfoxide (DMSO)	Sigma-Aldrich Merck	Cat# 472301
5-Bromo-2Deoxyuridina (BrdU)	BD Biosciences	Cat# 423361
Ethylenediaminetetraacetic acid (EDTA)	Sigma-Aldrich Merck	Cat# E6758

Lipopolysaccharide (LPS)	Sigma-Aldrich Merck	Cat# L4391
Triton X-100	Sigma-Aldrich Merck	Cat# T8787
TdT buffer	Invitrogen Thermo Fischer	Cat# 16314015
Biotin-16-dUTP	Sigma-Aldrich Merck	Cat# 11093070910
TUNEL Enzyme	Sigma-Aldrich Merck	Cat# 11767305001
DNase I	ZYMO research	Cat# E1011-A
Vectashield	Vector Laboratories	Cat# H-1000
Potassium chloride (KH_4Cl)	Sigma-Aldrich Merck	Cat# P9541
SMART-Seq® v4 Ultra® Low Input RNA Kit for Sequencing	Clontech TaKaRa Bio	Cat# 634892
Low Input Library Prep Kit v2	Clontech TaKaRa Bio	Cat# 634899
Chromium Next GEM Single Cell 3' Kit v3.1	10x Genomics	Cat# PN-1000123
Qubit™ dsDNA HS and BR Assay Kits	Invitrogen Thermo Fischer	Cat# Q32851
High Sensitivity DNA Kit	Agilent Technologies	Cat# 5067-4626
Saline Solution (NaCl) 9mg/mL	B. Braun	Cat# 363 2202
