# Supplementary material for: Endogenous IL-1 receptor antagonist restricts healthy and malignant myeloproliferation
Source: Nat Commun. 2023 Jan 3;14:12. doi: 10.1038/s41467-022-35700-9 (PMC9810723; doi:10.1038/s41467-022-35700-9)
Supplement: Supplementary file 3 — Description of Additional Supplementary Files [file 41467_2022_35700_MOESM3_ESM.pdf]

Description of Additional Supplementary Files

Supplementary Data 1: Complete NFκB gene list used to identify NFκB targets in RNA sequencing studies.

Supplementary Data 2: Complete lists of genes detected by single-cell RNA sequencing of FACS-sorted $CD11b^+$ cells from the bone marrow of C57BL/6J wild-type and IL-1rn knockout mice, as well as for each annotated cluster within this cell subset.

Supplementary Data 3: Complete lists of genes detected by single-cell RNA sequencing of FACS-sorted $Lin^-Sca\text{-}1^+c\text{-}Kit^+$ (LSK) cells from the bone marrow of C57BL/6J wild-type and IL-1rn knockout mice, as well as for each annotated cluster within this cell subset.

Supplementary Data 4: Complete lists of genes detected by single-cell RNA sequencing of FACS-sorted $CD63^+$ stromal cells from the bone marrow of C57BL/6J wild-type and IL-1rn knockout mice, as well as for each annotated cluster within this cell subset.
