# Supplementary material for: Endogenous IL-1 receptor antagonist restricts healthy and malignant myeloproliferation
Source: Nat Commun. 2023 Jan 3;14:12. doi: 10.1038/s41467-022-35700-9 (PMC9810723; doi:10.1038/s41467-022-35700-9)
Supplement: Supplementary file 8 — Reporting Summary [file 41467_2022_35700_MOESM8_ESM.pdf]

nature portfolio

Corresponding author(s): Lorena Arranz

Last updated by author(s): Nov 28, 2022

Reporting Summary

Nature Portfolio wishes to improve the reproducibility of the work that we publish. This form provides structure for consistency and transparency in reporting. For further information on Nature Portfolio policies, see our Editorial Policies and the Editorial Policy Checklist.

Statistics

For all statistical analyses, confirm that the following items are present in the figure legend, table legend, main text, or Methods section.

n/a	Confirmed	
☐	☒	The exact sample size (*n*) for each experimental group/condition, given as a discrete number and unit of measurement
☐	☒	A statement on whether measurements were taken from distinct samples or whether the same sample was measured repeatedly
☐	☒	The statistical test(s) used AND whether they are one- or two-sided<br>*Only common tests should be described solely by name; describe more complex techniques in the Methods section.*
☐	☒	A description of all covariates tested
☐	☒	A description of any assumptions or corrections, such as tests of normality and adjustment for multiple comparisons
☐	☒	A full description of the statistical parameters including central tendency (e.g. means) or other basic estimates (e.g. regression coefficient) AND variation (e.g. standard deviation) or associated estimates of uncertainty (e.g. confidence intervals)
☐	☒	For null hypothesis testing, the test statistic (e.g. *F*, *t*, *r*) with confidence intervals, effect sizes, degrees of freedom and *P* value noted<br>*Give P values as exact values whenever suitable.*
☒	☐	For Bayesian analysis, information on the choice of priors and Markov chain Monte Carlo settings
☒	☐	For hierarchical and complex designs, identification of the appropriate level for tests and full reporting of outcomes
☒	☐	Estimates of effect sizes (e.g. Cohen's *d*, Pearson's *r*), indicating how they were calculated

Our web collection on statistics for biologists contains articles on many of the points above.

Software and code

Policy information about availability of computer code

Data collection	Cell counter: OLS CASY v2.5<br>NucleoCounter NC-200, chemometec: Nucleoview v1.3<br>Cell Counter: Countess III Automated Cell Counter (ThermoFisher): 1.0.296.782<br>Plate reader assays: SoftMax Pro v5.4.1<br>qPCR: QuantStudio Real-Time PCR Software v1.3<br>Cytokine immuno-assays: Bio-Plex Manager v6.2<br>Immunofluorescence: ZEN 2011 SP3 black edition (Release 8.1.0, Build 484) and Leica Application Suite Advanced Fluorescence software v4.0<br>Immunohistochemistry: DP-BSW Ver.3.3.1 (Olympus)<br>RNA-FISH: ZEN 2011 SP3 black edition (Release 8.1.0, Build 484)<br>Flow cytometry: BD FACS DIVA v6.1.3, v8.0.1 and v9.0<br>Hematological analyzer: Pentra XL 80 v2.2.1, Procyte IPU v:00-34 Build 57 and Hemavet 950FS<br>RNA-sequencing: HCS 2.2.58, RTA 1.18.64, NextSeq 550 System Suite v4.0<br>Single cell RNA-sequencing: NextSeq 2000 Controller v1.4.1, RTA3<br>Amnis ImageStreamX: INSPIRE v.200.1.620.0
Data analysis	Plate reader assays: SoftMax Pro v5.4.1<br>qPCR: QuantStudio Real-Time PCR Software v1.7.1<br>Cytokine immuno-assays: Bio-Plex Manager v6.2<br>Immunofluorescence: ZEN 2011 SP3 black edition (Release 8.1.0, Build 484), Leica Application Suite Advanced Fluorescence software and ImageJ v1.8.0<br>Immunohistochemistry and RNA-FISH: ImageJ v1.8.0<br>Flow Cytometry: BD FACS DIVA v8.0.1 and v9.0, and Flow Jo v.9 and v10.7.1_CL<br>Aimplex: FCAPArray v3<br>Amnis ImageStreamX: IDEAS v6.2

Extraction and demultiplexing of fastq files: bcl2fastq Conversion Software v1.8.4
Alignment and Quantification:
STAR v2.7.8
Samtools v1.9
Gffread v2.2.1.3
Subread v2.0.1
Salmon v1.5.1 with the commands --numBootstraps 100 --seqBias --gcBias --validateMappings --minScoreFraction 0.2 --consensusSlack 0.1
Sleuth v0.30.0 to perform gene level estimation
Open access tools and packages used with RStudio v1.4.1106 and R v4.1.2:
Rsubread v1.22.2 using featureCounts tool
clusterProfiler v4.0.0 using bitr and gseGO functions, and or.MM.eg.db v3.13.0 annotation library
DESeq2 v1.32.0
edgeR v3.34.1
ggplot2 v3.3.3
ggvenn v0.1.9
ggrepel v0.9.1
patchwork v1.1.1
enrichplot v1.12.1 with gseaplot2 function to draw GSEA plot
biomaRt v2.48.1 and v2.48.3
data.table v1.14.0
ggsci v2.9
survival v3.2-11
survminer v0.4.9
GEOquery v2.62.2
gridExtra v2.3 Survival plots
Extraction and demultiplexing single-cell RNA-Seq: bcl2fastq v2.20.0.422, cellranger demux v6.1
Single-cell analysis:
- CellRanger v6.1.2 for alignment, filtering, barcode counting, and UMI counting.
- R v4.0.3 & v.4.1.1
- RStudio 2022.07.2
- Seurat v4.0.5 to perform cell filtering, normalization, scaling, integration, clustering, gene module scores and differential expression analyses and to perform Principal Component Analysis and apply tSNE algorithm (a function of Seurat v4.0.5). We also used Seurat to annotate - ggplot2 v3.3.5 to draw tSNE plots, bar-charts and pie-charts
- SingleR v1.4.0 for initial annotation CD11b+ experiment.
- AddModuleScore from Seurat package to score imported gene signatures.
Data calculation: Microsoft Excel 2016 and Microsoft Office 365 v2104
Statistical analysis: GraphPad Prism 9.1.2 and RStudio v1.4.1106

For manuscripts utilizing custom algorithms or software that are central to the research but not yet described in published literature, software must be made available to editors and reviewers. We strongly encourage code deposition in a community repository (e.g. GitHub). See the Nature Portfolio guidelines for submitting code & software for further information.

Data

Policy information about availability of data

All manuscripts must include a data availability statement. This statement should provide the following information, where applicable:

- Accession codes, unique identifiers, or web links for publicly available datasets
- A description of any restrictions on data availability
- For clinical datasets or third party data, please ensure that the statement adheres to our policy

The various RNA-Seq data generated in this study have been deposited in the GEO database under the following accession codes:
GSE126428 [https://www.ncbi.nlm.nih.gov/geo/query/acc.cgi?acc=GSE126428]
GSE126625 [https://www.ncbi.nlm.nih.gov/geo/query/acc.cgi?acc=GSE126625]
GSE157038 [https://www.ncbi.nlm.nih.gov/geo/query/acc.cgi?acc=GSE157038].

The scRNA-Seq data have accession code:
GSE197594 [https://www.ncbi.nlm.nih.gov/geo/query/acc.cgi?acc=GSE197594].

The rest of the raw data generated in this study are provided and can be found in the Supplementary Data and Source Data files provided with this paper.

Public datasets and databases used in this study in addition to the ones generated are the following:
GSE14468 [https://www.ncbi.nlm.nih.gov/geo/query/acc.cgi?acc=GSE14468]
GSE165810 [https://www.ncbi.nlm.nih.gov/geo/query/acc.cgi?acc=GSE165810]
GSE166629 [https://www.ncbi.nlm.nih.gov/geo/query/acc.cgi?acc=GSE166629]
GSE137539 [https://www.ncbi.nlm.nih.gov/geo/query/acc.cgi?acc=GSE137539]
GSE131834 [https://0-www-ncbi-nlm-nih-gov.brum.beds.ac.uk/geo/query/acc.cgi?acc=GSE131834]
GSE128423 [https://www.ncbi.nlm.nih.gov/geo/query/acc.cgi?acc=GSE128423]
GSE83533 [https://www.ncbi.nlm.nih.gov/geo/query/acc.cgi?acc=GSE83533]
GSE108892 [https://www.ncbi.nlm.nih.gov/geo/query/acc.cgi?acc=GSE108892]
E-MTAB-9208 [https://www.ebi.ac.uk/biostudies/arrayexpress/studies/E-MTAB-9208]
Synapse ID syn4956655 [https://www.synapse.org/#!Synapse:syn4956655/files]
[https://bioinfo.lifl.fr/NF-KB] (Dr. K. Gosselin, Institute of Biology of Lille, France)
[https://www.bu.edu/nf-kb/gene-resources/target-genes] (Dr. T. Gilmore, University of Boston, USA)
PRJNA774277 [https://www.ncbi.nlm.nih.gov/bioproject/?term=PRJNA774277]
Genome assembly GCF_000001635.25 [https://www.ncbi.nlm.nih.gov/assembly/GCF_000001635.25]

Ensembl Compara Database [https://www.ensembl.org/info/docs/api/compara/index.html]
Molecular Signatures Database [https://www.gsea-msigdb.org/gsea/msigdb]
Immunological Genome Project (ImmGen) database [https://www.immgen.org]
phs001027 from the database of Genotypes and Phenotypes [(dbGaP) https://www.ncbi.nlm.nih.gov/projects/gap/cgi-bin/study.cgi?study_id=phs001027.v4.p1]

Further information and requests should be directed to and will be fulfilled by the corresponding author, Dr. Lorena Arranz (lorena.arranz@uit.no).

Field-specific reporting

Please select the one below that is the best fit for your research. If you are not sure, read the appropriate sections before making your selection.

☒ Life sciences ☐ Behavioural & social sciences ☐ Ecological, evolutionary & environmental sciences

For a reference copy of the document with all sections, see nature.com/documents/nr-reporting-summary-flat.pdf

Life sciences study design

All studies must disclose on these points even when the disclosure is negative.

Sample size	For in vivo experiments, sample size was estimated based on the minimum number of animals required to obtain biologically meaningful results in studies of hematopoiesis as shown by cumulative scientific literature and own experience. Cohort size and numbers of cells transplanted in xenografts were informed by the total number of available cells. For human studies, all available samples were used.
Data exclusions	Animals that showed symptoms of disease or health issues unrelated to aberrant myelopoiesis were excluded from the study according to pre-established criteria: obesity, loss of weight unrelated to experimental conditions or excesive, tumor masses, skin inflammation, etc. Criteria applied for mouse termination before the established end point were in accordance with the Norwegian Food and Safety Authority. Not pre-established statistical outliers in mouse studies were excluded using Grubbs or Dixon test where appropriate. Outliers in human studies were not excluded.
Replication	In vivo treatments of AML xenografts with IL-1β or vehicle were performed twice. In vivo treatment of AML xenografts with IL-1RN, canakinumab or vehicle was repeated three times. Il1rn−/− and C57BL/6J WT mice were transplanted with NRASG12D+ (from previously induced Mx1-Cre NRASG12D mice) or control (from previously induced NRASG12D mice) BM nucleated cells, over four independent experiments. Where appropriate, experimental group size was increased by pooling results of samples from different mice of similar characteristics analyzed on different days based on availability of new experimental and wild-type or control mice. Most in vivo experiments were performed over two or more days, to ensure replication of results. Data of ex vivo cultures, cytokine analyses, molecular biology assays, histology and immunofluorescence from each mouse are the mean result of at least two or three technical replicates, and most of these assays were performed over two or more days with samples from different mice, to ensure replication of results. Human samples were used only once or twice due to low availability. Ex vivo experiments with thawed human samples, i.e. imaging flow cytometry, phospho-flow, IL-1RAP FACS, cytokine analyses and qRT-PCR, were performed over two or more days, to ensure replication of results.
Randomization	Mice were randomized to treatment groups. Mice of the same sex and approximate similar age were used to control for covariates. AML human samples were chosen based on availability and healthy controls were chosen as close as possible in age to the AML samples, based on availability.
Blinding	No blinding due to regulations at the Animal Facilities of the UiT – The Arctic University of Norway and the University of Oslo, Centro Nacional de Investigaciones Cardiovasculares and the Wisconsin Institutes for Medical Research Vivarium. Blinding was not feasible in case of human samples due to the different way that healthy volunteers and patients were recruited from the Stem Cells, Ageing and Cancer group members at UiT or from Dr. Anders Vik at the Department of Hematology, University Hospital of North Norway, respectively. At least two scientists performed the experiments and analyzed the data independently.

Reporting for specific materials, systems and methods

We require information from authors about some types of materials, experimental systems and methods used in many studies. Here, indicate whether each material, system or method listed is relevant to your study. If you are not sure if a list item applies to your research, read the appropriate section before selecting a response.

Materials & experimental systems

n/a	Involved in the study	
☐	☒	Antibodies
☐	☒	Eukaryotic cell lines
☒	☐	Palaeontology and archaeology
☐	☒	Animals and other organisms
☐	☒	Human research participants
☒	☐	Clinical data
☒	☐	Dual use research of concern

Methods

n/a	Involved in the study	
☒	☐	ChIP-seq
☐	☒	Flow cytometry
☒	☐	MRI-based neuroimaging

Antibodies

Antibodies used

-Flow cytometry
Anti-mouse
Alexa Fluor 647 rat anti-mouse CD11b BD Biosciences Cat# 557686; RRID AB_396796; cloneM1/70
PE rat anti-mouse Ly-6G/Ly-6C (Gr-1) BD Biosciences Cat# 553128; RRID AB_394644; clone RB6-8C5
FITC rat anti-mouse CD45R/B220 BD Biosciences Cat# 553088; RRID AB_394618; clone RA3-6B2
PE-Cy5 hamster anti-mouse CD3e BD Biosciences Cat# 553065; RRID AB_394598; clone 145-2C11
PE rat anti-mouse Ly-6G BD Biosciences Cat# 551461; RRID AB_394208; clone 1A8
PE rat anti-mouse Ly6A/E (Sca-1) BD Biosciences Cat# 553336; RRID AB_394792; clone E13-161.7
FITC rat anti-mouse CD34 BD Biosciences Cat# 553733; RRID AB_395017; clone RAM34
APC rat anti-mouse CD135 (Flt3) BD Biosciences Cat# 560718; RRID AB_1727425; clone A2F10.1
APC mouse anti-BrdU BD Biosciences Cat# 552598; RRID AB_2861367; clone B44
PE-Cy7 rat anti-mouse CD117 (c-Kit) BD Biosciences Cat# 558163; RRID AB_647250; clone 2B8
PE rat anti-mouse CD63 BD Biosciences Cat# 564222; RRID AB_2738678; clone NVG-2
PE rat anti-mouse CD106 (VCAM) BD Biosciences Cat# 561613; RRID AB_10897990; clone 429(MVCAM.A)
Alexa Fluor 647 rat anti-mouse CD121a (IL-1r1) BD Biosciences Cat# 563629; RRID AB_2738332; clone 35F5
PE-Cy7 anti-mouse CD105 (Endoglin) BioLegend Cat# 120410; RRID AB_1027700; clone MJ7/18
Alexa Fluor 488 Rat anti-mouse F4/80 eBioscience ThermoFisher Cat# 53-4801-82; RRID AB_469915; clone BM8
Biotin rat anti-mouse CD31 BD Biosciences Cat# 553371; RRID AB_394817; clone MEC13.3
Biotin rat anti-mouse TER-119/Erythroid cells BD Biosciences Cat# 553672; RRID AB_394985; clone TER-119
Biotin mouse anti-mouse CD45.2 BD Biosciences Cat# 553771; RRID AB_395040; clone 104
BB700 rat anti-mouse Ly6A/E (Sca-1) BD Biosciences Cat# 742089; RRID AB_2871369; clone D7
PE rat anti-mouse CD135 (Flt3) BD Biosciences Cat# 553842; RRID AB_395079; clone A2F10.1
PE rat anti-mouse CD16/32 (FcRγ) BD Biosciences Cat# 567020; RRID AB_2870010; clone Ab93
PE-Cy5 rat anti-mouse CD150 (SLAM) BioLegend Cat# 115912; RRID AB_493598; clone TC15-12F12.2
APC hamster anti-mouse CD48 BD Biosciences Cat# 562746; RRID AB_2737765; clone HM48-1
APC rat anti-mouse CD127 (IL-7R) BD Biosciences Cat# 564175; RRID AB_2732843; clone SB/199
Biotin Mouse Lineage Depletion Cocktail BD Biosciences Cat# 51-9000794
Pacific Blue conjugated Annexin V antibody Life technologies Cat# A35122
PE-Cy7 mouse anti-mouse CD45.1 BD Biosciences Cat# 560578; RRID: AB_1727488; clone A20
FITC rat anti-mouse CD45 BD Biosciences Cat# 553080; RRID: AB_394610; clone 30-F11
Alexa Fluor 647 rat anti-mouse CD34 BD Biosciences Cat# 560230; RRID AB_1645199; clone RAM34
FITC rabbit anti-mouse IL-1RAP ProteoGenix, customized batch #15893-113021-A01; clone R5P1-A1
PE-Cy7 rat anti-mouse F4/80 BioLegend Cat# 123113, RRID: AB_893490; clone BM8
Biotin rat anti-mouse CD45R (B220) eBioscience ThermoFisher Cat# 13-0452-82; RRID AB_466449; clone RA3-6B2
Biotin rat anti-mouse CD19 eBioscience ThermoFisher Cat# 13-0193-82; RRID AB_657656; clone eBio1D3 (1D3)
Biotin rat anti-mouse CD4 eBioscience ThermoFisher Cat# 13-0041-82; RRID AB_466325; clone GK1.5
Biotin rat anti-mouse CD8a eBioscience ThermoFisher Cat# 13-0081-82; RRID AB_466346; clone 53-6.7
Biotin armenian hamster anti-mouse CD3e eBioscience ThermoFisher Cat# 13-0031-82; RRID AB_466319; clone 145-2C11
Biotin rat anti-mouse IgM eBioscience ThermoFisher Cat# 13-5790-82; RRID AB_466675; clone II/41
Biotin rat anti-mouse Ly-6G/Ly-6C (Gr-1) eBioscience ThermoFisher Cat# 13-5931-82; RRID AB_466800; clone RB6-8C5
Biotin rat anti-mouse TER-119 eBioscience ThermoFisher Cat# 13-5921-82; RRID AB_466797; clone TER-119
FITC rat anti-mouse CD11b eBioscience ThermoFisher Cat# 11-0112-41; RRID AB_11042156; clone M1/70
PE rat anti-mouse Ly-6G/Ly-6C (Gr-1) eBioscience ThermoFisher Cat# 12-5931-82; RRID AB_466045; clone RB6-8C5
APC rat anti-mouse CD127 eBioscience ThermoFisher Cat# 17-1271-82; RRID AB_469435; clone A7R34
PerCP-Cy5.5 rat anti-mouse Ly-6A/E (Sca-1) eBioscience ThermoFisher Cat# 45-5981-82; RRID AB_914372; clone D7
FITC rat anti-mouse CD34 eBioscience ThermoFisher Cat# 11-0341-82; RRID AB_465021; clone RAM34
PE-Cy7 rat anti-mouse CD117 (c-Kit) eBioscience ThermoFisher Cat# 25-1171-82; RRID AB_469644; clone 2B8
APC rat anti-mouse CD16/CD32 (FcγRII/III) eBioscience ThermoFisher Cat# 17-0161-82; RRID AB_469356; clone 93
APC rat anti-mouse CD135 (Flt3) eBioscience ThermoFisher Cat# 17-1351-82; RRID AB_10717261; clone A2F10
APC-eFluor 780 Armenian hamster anti-mouse CD48 eBioscience ThermoFisher Cat# 47-0481-82; RRID AB_2573962; clone HM48-1
PE Rat anti-mouse CD150 (SLAM) BioLegend Cat# 115904; RRID AB_313683; clone TC15-12F12.2
FITC rat anti-mouse Ly-6A/E (Sca-1) eBioscience ThermoFisher Cat# 11-5981-82; RRID: AB_465333; clone D7
V450 rat anti-mouse CD45 BD Biosciences Cat# 560501; RRID: AB_1645275; clone 30-F11
Pacific Blue rat anti-mouse TER-119 BioLegend Cat# 116231; RRID: AB_2149212; clone TER-119
FITC rat anti-mouse CD31 (PECAM-1) eBiosciences Thermo Fisher Cat# 11-0311-85; RRID: AB_465013; clone 390
PE-Cy7 rat anti-mouse CD63 BioLegend Cat# 143910; RRID: AB_2565500; clone NVG-2

Anti-human:
FITC mouse anti-human CD45 BD Biosciences Cat# 345808; RRID AB_2732010; clone 2D1
PE mouse anti-human CD11b; BD Biosciences; Cat# 557321; RRID AB_396636; clone ICRF44
PE mouse anti-human CD33 BD Biosciences Cat# 555450; RRID AB 395843; clone WM53
FITC mouse anti-human CD34 BD Biosciences Cat# 560942; RRID AB 10562559; clone 581
Alexa Fluor 647 Mouse anti-NFκB p65 (pS529) BD Biosciences Cat# 558422; RRID AB_647136; clone K10-895.12.50
Alexa Fluor 647 Mouse IgG2b, κ Isotype Control BD Biosciences Cat# 558713; RRID AB_1645618; clone 27-35
Alexa Fluor 488 anti-human NFκB p50 Luminex Corporation Cat# 4700-1674
BV421 mouse anti-human IL-1RAcP (IL-1R3) BD Biosciences Cat# 748107; RRID AB_2872568; clone 89412
BV421 mouse IgG1, κ Isotype Control BD Biosciences Cat# 562438; RRID AB_11207319

-Immunofluorescence
Goat polyclonal anti-mouse IL-1r1 R&D system Cat# AF771; RRID AB_355587
Donkey polyclonal anti-goat IgG H&L (Cy3) Abcam Cat# ab6949; RRID AB_955018

-Treatments
Mouse IL-1β mAb Novus Biologicals Cat# NB600-1379 (discontinued), and Thermo Fisher Cat# MM425B; RRID AB_223529; clone 1400.24.17
IgG1 kappa isotype control Thermo Fisher Cat# 14-4714-82; RRID AB_470111; clone P3.6.2.8.1
InVivoMab hamster anti-mouse IL-1α BioXCell Cat# BE0243; RRID AB_2687724; clone ALF-161
InVivoMAb armenian hamster IgG isotype control BioXCell Cat# BE0091; RRID AB_1107773
Anti-human IL-1β therapeutic antibody (FDA-approved ILARIS (canakinumab) (Novartis) European Agency Number EMEA/H/C/001109)

-Cytokine assays
Bio-Plex Pro Human Cytokine IL-1β Set (Cat# 171B5001M – Bio-Rad)
Bio-Plex Pro Human Cytokine IL-1RA Set (Cat# 171B5002M – Bio-Rad)
Bio-Plex Pro Mouse Cytokine IL-1β Set (Cat# 171G5002M – Bio-Rad)
Bio-Plex Pro Mouse Cytokine IL-6 Set (Cat #171G5007M – Bio-Rad)
Bio-Plex Pro Mouse Cytokine IL-10 Set (Cat# 171G5009M – Bio-Rad)
Bio-Plex Pro Mouse Cytokine IFN-γ Set (Cat# 171G5017M – Bio-Rad)
Bio-Plex Pro Mouse Cytokine TNF-α Set (Cat# 171G5023M – Bio-Rad)
Mouse IL-1RA AimPlex bead-based immunoassay Aimplex Biosciences Cat# PN: B211239

Validation

Validation data of the antibodies purchased from commercial vendors are available on the manufacturer website and datasheets. Only FITC rabbit anti-mouse Il-1RAP in this study is a customized antibody produced by phage-display technology (ProteoGenix) as described in the Material and Methods section of this paper. Binding specificity was checked by ELISA. Purity, integrity and assembling were checked by SDS-PAGE.

Antibodies used for flow cytometry:

Anti-mouse:
Alexa Fluor 647 rat anti-mouse CD11b (Cat# 557686, BD Biosciences)
https://www.bdbiosciences.com/en-us/products/reagents/flow-cytometry-reagents/research-reagents/single-color-antibodies-ruo/alexa-fluor-647-rat-anti-cd11b.557686
PE rat anti-mouse Ly-6G/Ly-6C (Gr-1) (Cat# 553128, BD Biosciences)
https://www.bdbiosciences.com/en-us/products/reagents/flow-cytometry-reagents/research-reagents/single-color-antibodies-ruo/pe-rat-anti-mouse-ly-6g-and-ly-6c.553128
FITC rat anti-mouse CD45R/B220 (Cat# 553088, BD Biosciences)
https://www.bdbiosciences.com/en-us/products/reagents/flow-cytometry-reagents/research-reagents/single-color-antibodies-ruo/fitc-rat-anti-mouse-cd45r-b220.553088
PE-Cy5 hamster anti-mouse CD3e (Cat# 553065, BD Biosciences)
https://www.bdbiosciences.com/en-us/products/reagents/flow-cytometry-reagents/research-reagents/single-color-antibodies-ruo/pe-cy-5-hamster-anti-mouse-cd3e.553065
PE rat anti-mouse Ly-6G (Cat# 551461, BD Biosciences)
https://www.bdbiosciences.com/en-us/products/reagents/flow-cytometry-reagents/research-reagents/single-color-antibodies-ruo/pe-rat-anti-mouse-ly-6g.551461
PE rat anti-mouse Ly6A/E (Sca-1) (Cat# 553336, BD Biosciences)
https://www.bdbiosciences.com/en-us/products/reagents/flow-cytometry-reagents/research-reagents/single-color-antibodies-ruo/pe-rat-anti-mouse-ly-6a-e.553336
FITC rat anti-mouse CD34 (Cat# 553733, BD Biosciences)
https://www.bdbiosciences.com/en-us/products/reagents/flow-cytometry-reagents/research-reagents/single-color-antibodies-ruo/fitc-rat-anti-mouse-cd34.553733
APC rat anti-mouse CD135 (Flt3) (Cat# 560718, BD Biosciences)
https://www.bdbiosciences.com/en-us/products/reagents/flow-cytometry-reagents/research-reagents/single-color-antibodies-ruo/apc-rat-anti-mouse-cd135.560718
APC mouse anti-BrdU (APC BrdU Flow Kit) (Cat# 552598, BD Biosciences)
https://www.bdbiosciences.com/en-us/products/reagents/flow-cytometry-reagents/research-reagents/cell-function-analysis-stains-dyes/apc-brdu-kit.552598
PE-Cy7 rat anti-mouse CD117 (c-Kit) (Cat# 558163, BD Biosciences)
https://www.bdbiosciences.com/en-us/products/reagents/flow-cytometry-reagents/research-reagents/single-color-antibodies-ruo/pe-cy-7-rat-anti-mouse-cd117.558163
PE rat anti-mouse CD63 (Cat# 564222, BD Biosciences)
https://www.bdbiosciences.com/en-us/products/reagents/flow-cytometry-reagents/research-reagents/single-color-antibodies-ruo/pe-rat-anti-mouse-cd63.564222
PE rat anti-mouse CD106 (VCAM) (Cat# 561613, BD Biosciences)
https://www.bdbiosciences.com/en-us/products/reagents/flow-cytometry-reagents/research-reagents/single-color-antibodies-ruo/pe-rat-anti-mouse-cd106.561613
Alexa Fluor 647 rat anti-mouse CD121a (IL-1r1) (Cat# 563629, BD Biosciences)
https://www.bdbiosciences.com/en-us/products/reagents/flow-cytometry-reagents/research-reagents/single-color-antibodies-ruo/alexa-fluor-647-rat-anti-mouse-cd121a.563629
PE-Cy7 anti-mouse CD105 (Endoglin) (Cat# 120410, BioLegend)
https://www.biolegend.com/en-us/products/pe-cyanine7-anti-mouse-cd105-antibody-4573?GroupID=BLG10724
Alexa Fluor 488 Rat anti-mouse F4/80 (Cat# 53-4801-82, eBioscience)
https://www.thermofisher.com/antibody/product/F4-80-Antibody-clone-BM8-Monoclonal/53-4801-82
Biotin rat anti-mouse CD31 (Cat# 553371, BD Biosciences)
https://www.bdbiosciences.com/en-us/products/reagents/flow-cytometry-reagents/research-reagents/single-color-antibodies-ruo/biotin-rat-anti-mouse-cd31.553371
Biotin rat anti-mouse TER-119/Erythroid cells (Cat# 553672, BD Biosciences)
https://www.bdbiosciences.com/en-us/products/reagents/flow-cytometry-reagents/research-reagents/single-color-antibodies-ruo/biotin-rat-anti-mouse-ter-119-erythroid-cells.553672

Biotin mouse anti-mouse CD45.2 (Cat# 553771, BD Biosciences)
https://www.bdbiosciences.com/en-us/products/reagents/flow-cytometry-reagents/research-reagents/single-color-antibodies-ruo/biotin-mouse-anti-mouse-cd45-2.553771
BB700 rat anti-mouse Ly6A/E (Sca-1) (Cat# 742089, BD Biosciences)
https://www.bdbiosciences.com/en-us/products/reagents/flow-cytometry-reagents/research-reagents/single-color-antibodies-ruo/bb700-rat-anti-mouse-ly-6a-e.742089
PE rat anti-mouse CD135 (Flt3) (Cat# 553842, BD Biosciences)
https://www.bdbiosciences.com/en-us/products/reagents/flow-cytometry-reagents/research-reagents/single-color-antibodies-ruo/pe-rat-anti-mouse-cd135.553842
PE rat anti-mouse CD16/32 (FcRγ) (Cat# 567020, BD Biosciences)
https://www.bdbiosciences.com/en-us/products/reagents/flow-cytometry-reagents/research-reagents/single-color-antibodies-ruo/pe-rat-anti-mouse-cd16-cd32.567020
PE-Cy5 rat anti-mouse CD150 (SLAM) (Cat# 115912, Biolegend)
https://www.biolegend.com/en-us/products/pe-cyanine5-anti-mouse-cd150-slam-antibody-2895
APC hamster anti-mouse CD48 (Cat# 562746, BD Biosciences)
https://www.bdbiosciences.com/en-us/products/reagents/flow-cytometry-reagents/research-reagents/single-color-antibodies-ruo/apc-hamster-anti-mouse-cd48.562746
APC rat anti-mouse CD127 (IL-7R) (Cat# 564175, BD Biosciences)
https://www.bdbiosciences.com/en-us/products/reagents/flow-cytometry-reagents/research-reagents/single-color-antibodies-ruo/apc-rat-anti-mouse-cd127.564175
Biotin Mouse Lineage Depletion Cocktail (Cat# 51-9000794, BD Biosciences)
https://www.bdbiosciences.com/content/dam/bdb/products/global/reagents/cell-preparation-separation-reagents/magnetic-cell-separation/558451_base/pdf/558451.pdf
Pacific Blue conjugated Annexin V (Cat# A35122, Life Technologies)
https://www.thermofisher.com/order/catalog/product/A35122#/A35122
PE-Cy7 mouse anti-mouse CD45.1 (Cat# 560578, BD Biosciences)
https://www.bdbiosciences.com/en-us/products/reagents/flow-cytometry-reagents/research-reagents/single-color-antibodies-ruo/pe-cy-7-mouse-anti-mouse-cd45-1.560578
FITC rat anti-mouse CD45 (Cat# 553080, BD Biosciences)
https://www.bdbiosciences.com/en-us/products/reagents/flow-cytometry-reagents/research-reagents/single-color-antibodies-ruo/fitc-rat-anti-mouse-cd45.553080
Alexa Fluor® 647 Rat anti-Mouse CD34 (Cat# 560230, BD Biosciences)
https://www.bdbiosciences.com/en-us/products/reagents/flow-cytometry-reagents/research-reagents/single-color-antibodies-ruo/alexa-fluor-647-rat-anti-mouse-cd34.560230
FITC Rabbit anti-mouse IL-1RAP, clone R5P1-A1 (ProteoGenix) Batch #15893-113021-A01
Monoclonal antibody produced by phage-display technology (see Material and Methods section of this paper, ProteoGenix).
Specificity of binding validated by ELISA. Purity, integrity and correct assembling checked by SDS-PAGE.
PE-Cy7 rat anti-mouse F4/80 (Cat# 123113, BioLegend)
https://www.biolegend.com/en-us/products/pe-cyanine7-anti-mouse-f4-80-antibody-4070?GroupID=BLG5319
Biotin Rat anti-mouse CD45R (B220) (Cat# 13-0452-82, eBioscience)
https://www.thermofisher.com/antibody/product/CD45R-B220-Antibody-clone-RA3-6B2-Monoclonal/13-0452-82
Biotin Rat anti-mouse CD19 (Cat# 13-0193-82, ThermoFisher)
https://www.thermofisher.com/antibody/product/CD19-Antibody-clone-eBio1D3-1D3-Monoclonal/13-0193-82
Biotin Rat anti-mouse CD4 (Cat# 13-0041-82, ThermoFisher)
https://www.thermofisher.com/antibody/product/CD4-Antibody-clone-GK1-5-Monoclonal/13-0041-82
Biotin Rat anti-mouse CD8a (Cat# 13-0081-82, ThermoFisher)
https://www.thermofisher.com/antibody/product/CD8a-Antibody-clone-53-6-7-Monoclonal/13-0081-82
Biotin Armenian hamster anti-mouse CD3e (Cat# 13-0031-82, ThermoFisher)
https://www.thermofisher.com/antibody/product/CD3e-Antibody-clone-145-2C11-Monoclonal/13-0031-82
Biotin Rat anti-mouse IgM (Cat# 13-5790-82, ThermoFisher)
https://www.thermofisher.com/antibody/product/IgM-Antibody-clone-II-41-Monoclonal/13-5790-82
Biotin Rat anti-mouse Ly-6G/Ly-6C (Gr-1) (Cat# 13-5931-82, ThermoFisher)
https://www.thermofisher.com/antibody/product/Ly-6G-Ly-6C-Antibody-clone-RB6-8C5-Monoclonal/13-5931-82
Biotin Rat anti-mouse TER-119 (Cat# 13-5921-82, ThermoFisher)
https://www.thermofisher.com/antibody/product/TER-119-Antibody-clone-TER-119-Monoclonal/13-5921-82
FITC Rat anti-mouse CD11b (Cat# 11-0112-41, ThermoFisher)
https://www.thermofisher.com/antibody/product/CD11b-Antibody-clone-M1-70-Monoclonal/11-0112-41
PE Rat anti-mouse Ly-6G/Ly-6C (Gr-1) (Cat# 12-5931-82, ThermoFisher)
https://www.thermofisher.com/antibody/product/Ly-6G-Ly-6C-Antibody-clone-RB6-8C5-Monoclonal/12-5931-82
APC Rat anti-mouse CD127 (Cat# 17-1271-82, ThermoFisher)
https://www.thermofisher.com/antibody/product/CD127-Antibody-clone-A7R34-Monoclonal/17-1271-82
PerCP-Cy5.5 Rat anti-mouse Ly-6A/E (Sca-1) (Cat# 45-5981-82, ThermoFisher)
https://www.thermofisher.com/antibody/product/Ly-6A-E-Sca-1-Antibody-clone-D7-Monoclonal/45-5981-82
FITC Rat anti-mouse CD34 (Cat# 11-0341-82, ThermoFisher)
https://www.thermofisher.com/antibody/product/CD34-Antibody-clone-RAM34-Monoclonal/11-0341-82
PE-Cy7 Rat anti-mouse CD117 (c-Kit) (Cat# 25-1171-82, ThermoFisher)
https://www.thermofisher.com/antibody/product/CD117-c-Kit-Antibody-clone-2B8-Monoclonal/25-1171-82
APC Rat anti-mouse CD16/CD32 (FcγRII/III) (Cat# 17-0161-82, ThermoFisher)
https://www.thermofisher.com/antibody/product/CD16-CD32-Antibody-clone-93-Monoclonal/17-0161-82
APC Rat anti-mouse CD135 (Flt3) (Cat# 17-1351-82, ThermoFisher)
https://www.thermofisher.com/antibody/product/CD135-Flt3-Antibody-clone-A2F10-Monoclonal/17-1351-82
APC-eFluor 780 Armenian hamster anti-mouse CD48 (Cat# 47-0481-82)
https://www.thermofisher.com/antibody/product/CD48-Antibody-clone-HM48-1-Monoclonal/47-0481-82
PE Rat anti-mouse CD150 (SLAM) (Cat# 115904, BioLegend)
https://www.biolegend.com/en-us/search-results/pe-anti-mouse-cd150-slam-antibody-1369
FITC rat anti-mouse Ly-6A/E (Sca-1) (Cat# 11-5981-82)https://www.thermofisher.com/antibody/product/Ly-6A-E-Sca-1-Antibody-

clone-D7-Monoclonal/11-5981-82
V450 rat anti-mouse CD45 (Cat# 560501)
https://www.bdbiosciences.com/en-es/products/reagents/flow-cytometry-reagents/research-reagents/single-color-antibodies-ruo/v450-rat-anti-mouse-cd45.560501
Pacific Blue rat anti-mouse TER-119 (Cat# 116231)
https://www.biolegend.com/en-us/products/pacific-blue-anti-mouse-ter-119-erythroid-cells-antibody-6137
FITC rat anti-mouse CD31 (PECAM-1) (Cat# 11-0311-82)
https://www.thermofisher.com/antibody/product/CD31-PECAM-1-Antibody-clone-390-Monoclonal/11-0311-82
PE-Cy7 rat anti-mouse CD63 (Cat# 143910)
https://www.biolegend.com/en-us/products/pecyanine7-anti-mouse-cd63-antibody-11722

Anti-human:
FITC mouse anti-human CD45 (Cat# 345808, BD Biosciences)
Discontinued under stated Cat#, now sold with GMP grade under Cat# 347463
https://www.bdbiosciences.com/en-us/products/reagents/flow-cytometry-reagents/clinical-discovery-research/single-color-antibodies-ruo-gmp/fitc-mouse-anti-human-cd45.347463
PE Mouse Anti-Human CD11b (Cat# 557321, BD Biosciences)
https://www.bdbiosciences.com/en-us/products/reagents/flow-cytometry-reagents/research-reagents/single-color-antibodies-ruo/pe-mouse-anti-human-cd11b.557321
PE mouse anti-human CD33 (Cat# 555450, BD Biosciences)
https://www.bdbiosciences.com/en-us/products/reagents/flow-cytometry-reagents/research-reagents/single-color-antibodies-ruo/pe-mouse-anti-human-cd33.555450
FITC mouse anti-human CD34 (Cat# 560942, BD Biosciences)
https://www.bdbiosciences.com/en-us/products/reagents/flow-cytometry-reagents/research-reagents/single-color-antibodies-ruo/fitc-mouse-anti-human-cd34.560942
Alexa Fluor 647 Mouse anti-NF-κB p65 (pS529) (Cat# 558422, BD Biosciences)
https://www.bdbiosciences.com/en-us/products/reagents/flow-cytometry-reagents/research-reagents/single-color-antibodies-ruo/alexa-fluor-647-mouse-anti-nf-b-p65-ps529.558422
Alexa Fluor 647 Mouse IgG2b, κ Isotype Control (Cat# 558713, BD Biosciences)
https://www.bdbiosciences.com/en-us/products/reagents/flow-cytometry-reagents/research-reagents/single-color-antibodies-ruo/alexa-fluor-647-mouse-igg2b-isotype-control.558713
Anti-Hu NFκB (p50) Alexa Fluor® 488 (Cat# 4700-1674, Luminex Corporation)
https://www.luminexcorp.com/amnis-nfkb-translocation-kit/#overview
BV421 Mouse Anti-Human IL-1 RAcP (IL-1R3) (Cat# 748107, BD Biosciences)
https://www.bdbiosciences.com/en-us/products/reagents/flow-cytometry-reagents/research-reagents/single-color-antibodies-ruo/bv421-mouse-anti-human-il-1-racp-il-1r3.748107
BV421 Mouse IgG1, k Isotype Control (Cat# 562438, BD Biosciences)
https://www.bdbiosciences.com/en-us/products/reagents/flow-cytometry-reagents/research-reagents/flow-cytometry-controls-and-lysates/bv421-mouse-igg1-k-isotype-control.562438

Antibodies used for immunofluorescence:
Goat polyclonal anti-mouse IL-1r1 (Cat# AF771, R&D system)
https://www.rndsystems.com/products/mouse-il-1-ri-antibody_af771
Donkey polyclonal anti-goat IgG H&L (Cy3) (Cat# ab6949, Abcam)
https://www.abcam.com/donkey-goat-igg-hl-cy3-preadsorbed-ab6949.html

Antibodies used for in vivo treatments:
mouse IL-1β mAb clone 1400.24.17 (Cat# NB600-1379, Novus Biologicals (discontinued) and Cat# MM425B, ThermoFisher)
https://www.novusbio.com/products/il-1-beta-il-1f2-antibody-14002417_nb600-1379
https://www.thermofisher.com/antibody/product/IL-1-beta-Antibody-clone-1400-24-17-Monoclonal/MM425B
IgG1 kappa isotype control (Cat# 14-4714-82, ThermoFisher)
https://www.thermofisher.com/antibody/product/Mouse-IgG1-kappa-clone-P3-6-2-8-1-Isotype-Control/14-4714-82
InVivoMab hamster anti-mouse IL-1α clone ALF-161 (Cat# BE0243, BioXCell)
https://bxcell.com/product/anti-m-il-1-alpha/
InVivoMAb Armenian hamster IgG isotype control (Cat# BE0091, BioXCell)
https://bxcell.com/product/polyclonal-3
human IL-1β mAb (FDA-approved ILARIS (canakinumab), Novartis)
https://www.novartis.us/sites/www.novartis.us/files/ilaris.pdf

Antibodies used for cytokine analysis
Bio-Plex Pro Human Cytokine IL-1β Set (Cat# 171B5001M – Bio-Rad)
https://www.bio-rad.com/en-no/sku/171b5001m-bio-plex-pro-human-cytokine-il-1%CE%B2-set?ID=171B5001M
Bio-Plex Pro Human Cytokine IL-1RA Set (Cat# 171B5002M – Bio-Rad)
https://www.bio-rad.com/en-no/sku/171b5002m-bio-plex-pro-human-cytokine-il-1ra-set?ID=171B5002M
Bio-Plex Pro Mouse Cytokine IL-1β Set (Cat# 171G5002M – Bio-Rad)
https://www.bio-rad.com/en-no/sku/171g5002m-bio-plex-pro-mouse-cytokine-il-1beta-set?ID=171G5002M
Bio-Plex Pro Mouse Cytokine IL-6 Set (Cat #171G5007M – Bio-Rad)
https://www.bio-rad.com/en-no/sku/171g5007m-bio-plex-pro-mouse-cytokine-il-6-set?ID=171G5007M
Bio-Plex Pro Mouse Cytokine IL-10 Set (Cat# 171G5009M – Bio-Rad)
https://www.bio-rad.com/en-no/sku/171g5009m-bio-plex-pro-mouse-cytokine-il-10-set?ID=171G5009M
Bio-Plex Pro Mouse Cytokine IFN-γ Set (Cat# 171G5017M – Bio-Rad)
https://www.bio-rad.com/en-no/sku/171g5017m-bio-plex-pro-mouse-cytokine-ifn-%CE%B3-set?ID=171G5017M
Bio-Plex Pro Mouse Cytokine TNF-α Set (Cat# 171G5023M – Bio-Rad)
https://www.bio-rad.com/en-no/sku/171g5023m-bio-plex-pro-mouse-cytokine-tnf-alpha-set?ID=171G5023M
Mouse IL-1RA AimPlex bead-based immunoassay (Cat# PN: B211239, Aimplex Biosciences)

https://www.aimplexbio.com/mil1ra

Eukaryotic cell lines

Policy information about cell lines

Cell line source(s)	THP-1: DSMZ ACC16;<br>XtenCHO ™ Cells: Proteogenix PX-XTE-004, property of Proteogenix, derived from CHO-K1 cell line
Authentication	From supplier: https://www.dsmz.de/collection/catalogue/details/culture/ACC-16;<br>https://www.proteogenix.science/wp-content/uploads/documents/userguide/UserGuide.pdf
Mycoplasma contamination	Cell lines were not tested for mycoplasma contamination
Commonly misidentified lines (See ICLAC register)	None

Animals and other organisms

Policy information about studies involving animals; ARRIVE guidelines recommended for reporting animal research

Laboratory animals

Mice
Age and gender matched Il1rn−/− (Hirsch et al., 1996), B6.SJL (CD45.1+), C57BL/6J, immunodeficient NOD Scid Gamma (NSG) mice expressing human IL3, GM-CSF and SCF (NSG-SGM3) (Billerbeck et al., 2011; Wunderlich et al., 2010), Nes-gfp (Mendez-Ferrer et al., 2010), Mx1-Cre NRASG12D (Li et al., 2011; Haigis et al., 2008) and Vav-Cre NRASG12D (You et al., 2021) were used in experiments. Phenotyping of Il1rn−/− mice versus C57BL/6J wild-type (WT) mice was performed in either females or males aged 7-61 weeks. Cre expression in Mx1-Cre NRASG12D mice was induced by injection of poly-inosine:poly-cytosine (polyI:polyC, Sigma-Aldrich Merck). Phenotyping of Mx1-Cre NRASG12D mice versus NRASG12D control littermates was performed in either females or males aged 14-69 weeks, 6-44 weeks after polyI:polyC induction. Animals were used as donors at least 4 weeks after polyI:polyC induction and they displayed splenomegaly. Group and individual characteristics of animals used in each experiment are provided in Supplementary Table S1.

Transplantation assays
BM transplantation was performed through the tail vein after myeloablation. For most myeloablation experiments, 6-20 week old female mice were used. Nes-gfp male mice were 28 weeks old at the time of transplantation. Male or female 7-16 week old NSG-SGM3 mice, were used in xenografts. Group and individual characteristics of animals used in each experiment are provided in Supplementary Table S1.

In vivo pharmacological treatments
In the NSG-SGM3 AML xenograft mouse model, treatments started 4-7 weeks post-transplant, when animals evidenced signs of engraftment. Human CD34+ transplanted NSG-SGM3 mice were injected with human IL-1β or saline solution for 16 weeks (AML7) or for 4 weeks (AML9). Human BM CD34+ transplanted NSG-SGM3 mice were injected with human IL-1RN or saline solution for 4 weeks (AML21, 22, 23). To compare IL-1RN with IL-1β blockade, mice were injected with anti-human IL-1β monoclonal antibody (mAb) canakinumab and they were analyzed at the same time point. Human BM nucleated cell transplanted NSG-SGM3 mice were injected with human IL-1RN or saline solution alone, for a total of 36 weeks (AML3).
For IL-1RN treatment in vivo, Il1rn−/− mice (9-16 weeks) were injected with human IL-1RN or saline solution alone for 10 weeks. For IL-1β mAb treatment in vivo, Il1rn−/− mice (9-21 weeks) were injected with anti-mouse IL-1β mAb or IgG1 kappa isotype control, for a total of 18 weeks. For IL-1α mAb treatment in vivo, Il1rn−/− mice (15-22 weeks) were injected with anti-mouse IL-1α mAb or IgG kappa isotype control for 8 weeks. For bortezomib treatment, Il1rn−/− mice (18-34 weeks) were injected with bortezomib or DMSO in PBS solution alone and they were analyzed 12 weeks after the start of the treatment.
Treatments were initiated after animals evidenced signs of disease in the Mx1-Cre NRASG12D mouse model of expanded myelopoiesis after induction. Primary mutant mice (12-13 weeks; 2 weeks after polyI:polyC induction) were injected with human IL-1RN or saline solution for 4 weeks. Primary mutant mice (59-62 weeks; 52 weeks after polyI:polyC induction) were injected with bortezomib or DMSO in PBS solution, and were analyzed 8 weeks after the start of the treatment. Group and individual characteristics of animals used in each experiment are provided in Supplementary Table S1.

RNA sequencing and bioinformatic data analysis
FACS-sorted LT-HSC, ST-HSC, MPP and CD63+ stromal cells obtained from the BM of Il1rn−/− and C57BL/6J WT female mice aged 22-25 weeks were used for RNA-Seq. FACS-sorted LT-HSC, ST-HSC and MPP obtained from the BM of male NRASG12D+ (induced Mx1-Cre NRASG12D) mice and control (induced NRASG12D) littermates 6 weeks after polyI:polyC induction, aged 34 weeks, were used for RNA-Seq. 28 week-old Nes-gfp male mice were transplanted with BM cells from previously polyI:polyC induced control (induced NRASG12D) or NRASG12D+ (induced Mx1-Cre NRASG12D) mice, and Nes-GFP+ cells were FACS-sorted from the BM for RNA-Seq, 4 weeks after the transplant.
FACS-sorted LSK, CD11b+ myeloid cells and CD63+ stromal cell cells were obtained from the BM of Il1rn−/− and C57BL/6J WT female mice aged 13-15 weeks, and used for single-cell RNA-Seq.
Group and individual characteristics of animals used in each experiment are provided in Supplementary Table S1.

Wild animals

The study did not involve wild animals.

Field-collected samples

The study did not involve field-collected samples.

Ethics oversight

Mouse experiments were conducted with the ethical approval of the Norwegian Food and Safety Authority (7141, 7960, 8043, 8252, 8408, 8660, 8667, 9005, 21740, 24009, 24065, 24736, 24737), the local Animal Care and Ethics Committees at UiT – The Arctic University of Norway (09/16 and 09/21), University of Oslo (Norway; A015) and CNIC (Spain; PROEX 101/18), and in accordance with

the Guide for the Care and Use of Laboratory Animals and approved by an Animal Care and Use Committee at UW-Madison (USA; M005328). The latter program is accredited by the Association for Assessment and Accreditation of Laboratory Animal Care.

Note that full information on the approval of the study protocol must also be provided in the manuscript.

Human research participants

Policy information about studies involving human research participants

Population characteristics

Patient group was formed by 24 AML patients and 2 MDS patients; 8 women (30.8%) and 18 men (69.2%) aged 66 years (range 52-94). All samples were collected at diagnosis time. Control group was formed by 35 healthy volunteers; 22 women (62.9%) and 13 men (37.1%) aged 42 years (range 22-79). Group characteristics of patient and healthy donor samples (age and gender) used in each experiment are provided in Supplementary Table S1. Subjects did not provide the full set of data.

Recruitment

AML and MDS patients were recruited from the University Hospital of Northern Norway (UNN) through the Department of Hematology (Anders Vik). Healthy volunteers were recruited from UiT and UNN staff, friends and relatives of Stem Cells, Ageing and Cancer group members. AML human samples were chosen based on availability and healthy controls were chosen as close as possible in age to the AML samples, based on availability. However, due to limited availability, the AML patient group is slightly older than the healthy donor group. Because of the same reason, it was not possible to match the sex of both groups, resulting in a slightly men biased AML group and female biased healthy control group. Given that the biases are only partial and we always used some samples of the same age for all experiments, the biases are expected to have only minor impact on the results. In addition, the inflammatory phenotype associated to aggressive AML is likely to be stronger than a potential inflammatory phenotype associated to minor biases in age or gender.

Ethics oversight

Written informed consent was obtained in accordance with the Norwegian legislation and the Declaration of Helsinki. Human studies were approved by the Regional Committee for Medical Research Ethics North Norway (REC North 2015/1082). Participants received no compensation.

Note that full information on the approval of the study protocol must also be provided in the manuscript.

Flow Cytometry

Plots

Confirm that:

☒ The axis labels state the marker and fluorochrome used (e.g. CD4-FITC).

☒ The axis scales are clearly visible. Include numbers along axes only for bottom left plot of group (a 'group' is an analysis of identical markers).

☒ All plots are contour plots with outliers or pseudocolor plots.

☒ A numerical value for number of cells or percentage (with statistics) is provided.

Methodology

Sample preparation

Mouse samples were processed as previously described (Arranz et al., Nature, 2014), with minor modifications as detailed below.
For bone marrow nucleated cells analysis, bones were cleaned from surrounding tissue and crushed in a mortar, filtered through a 40-µm mesh to obtain single cell suspensions, and depleted of red blood cells by lysis in 0.15M NH_4Cl for 10min at 4°C. The resulting nucleated cell suspensions were centrifuged, washed, and suspended in PBS buffer containing 2% fetal bovine serum (FBS) for further analyses. Labeling of single-cell suspensions with the corresponding antibodies was performed in PBS - 2% FBS for 30 to 40 minutes on ice in the dark. Lineage-negative fraction was enriched from bone marrow by magnetic-bead separation. For sorting of CD11b+ and LSK for scRNA-seq, bone marrow cells were flushed by centrifugation from 1 femur and 1 tibia before red blood cell lysis in buffer containing 0.15M KH_4Cl, and resuspension in PBS 0.05% BSA. Labeling of single-cell suspensions with the corresponding antibodies was performed on ice in PBS - 2% FBS. FACS-sorting was performed in PBS 0.05% BSA. The rest of the bones were pooled with all bones of another mouse for CD63+ stromal cell sorting as described below. For data obtained from Vav-Cre NRAS-G12D, samples were processed as previously described (Zhang et al., Blood, 2009). Briefly, cells were isolated from bone marrow by flushing with needle, lysed in NH_4Cl solution (StemCell Technologies), resuspended in PBS with 2% FBS and passed through 25-µm cell strainers to obtain single-cell suspensions prior to antibody staining.
For bone marrow stromal cells analysis, bones were cleaned from surrounding tissue, crushed in a mortar with a pestle, and collagenase-digested (catalogue number 07902, StemCell Technologies) in an orbital shaking water bath at 37°C for 1h. Cells were filtered through a 40-µm mesh and erythrocytes were lysed as previously described, in either 0.15M NH_4Cl or 0.15M KH_4Cl. The resulting bone marrow cell suspensions were centrifuged, washed, and suspended in PBS buffer containing 2% FBS for further analyses. Labeling of single-cell suspensions with the corresponding antibodies was performed on ice in PBS - 2% FBS for 30 minutes on ice in the dark. Sorting of CD63+ stromal cells for scRNA-seq was perfomed in PBS 0.05% BSA.
For blood samples, 200 µL of peripheral blood were obtained through the saphenous vein, with no anesthesia. Blood samples were collected in ethylenediaminetetraacetic acid (EDTA)-coated tubes (Microvette, Sarstedt), and used for blood cell counts (ABX Pentra XL 80, HORIBA or ProCyte Dx Hematology Analyzer, IDEXX) and flow cytometry. 20-100 µL of peripheral blood were lysed for erythrocytes as previously described, washed with PBS buffer containing 2% FBS and labeled with the corresponding antibodies. For data obtained from Vav-Cre NRAS-G12D mice, blood count was obtained using a Hemavet 950FS (Drew Scientific). Red blood cells were lysed in NH_4Cl solution (StemCell Technologies) and resuspended in PBS with 2% FBS prior to antibody staining for flow cytometry analyses.
For cell sorting, HSC and progenitors were enriched or not in lineage-negative cells by immune magnetic depletion of differentiated cells, depending on the experiment. Cells were stained with the specific antibodies to identify LT-HSC, ST-HSC

and MPP. LSK cells were sorted from total bone marrow with no enrichment step. For mesenchymal stromal cell (MSC) sorting, cells were identified as CD45.2–Ter119–CD31–CD63+. Myeloid cells were identified as CD11b+, granulocytes as CD11b+Gr-1hif4/80– and monocytes as CD11b+Gr-1+f4/80+, for cell sortings from total bone marrow.
Proliferation was analyzed after 5-Bromo-2-Deoxyuridine (BrdU) (BD Biosciences) injection in vivo. Mice were euthanized 24h post-injection and bone marrow nucleated cells were collected. Lineage-negative cells were enriched, and after surface antibody staining to identify CD34– LSK (LT-HSC) and CD34+ LSK (ST-HSC/MPP) cells, these were co-stained with anti-BrdU APC and DAPI following manufacturer guidelines.
For apoptosis, stromal cells were enriched in CD45.2–Ter-119–CD31– cells by immune magnetic depletion of positive cells. Bone marrow nucleated cells and enriched CD45.2–Ter-119–CD31– stromal cells were labeled with surface antibodies, to identify LSK or CD45.2–Ter-119–CD31–CD63+ cells respectively, washed with cold PBS and subsequently stained at room temperature with Pacific Blue-conjugated Annexin V antibody and 7-Aminoactinomycin D (7-AAD) according to the manufacturer guidelines.
Bone marrow and tibia were flushed with 150µL of cold PBS, centrifuged at 15000g for 10 minutes and bone marrow extracellular fluid harvested in the supernatant. Cytokines were measured by BioPlex, except IL-1rn that was measured by flow cytometry.

Human samples:
Peripheral blood mononucleated cells (PBMC) and bone marrow nucleated cells from AML patients, and PBMC from healthy controls were obtained by density gradient centrifugation (Lympholyte, Cedarlane), and were used for CD34+ cell enrichment using immune magnetic technology (Stem Cell Technologies). Labeling of single-cell suspensions with fluorochrome-conjugated antibody was performed in PBS - 2% FBS for 30 minutes on ice in the dark.
For quantification of NFκB phosphorylation by phospho-flow, cells were fixed with 4 % formaldehyde for 15 min at room temperature, washed with PBS and permeabilized with pre-chilled 90 % methanol for 10 min at 4 °C.
For quantification of NFκB nuclear translocation by imaging cytometry, single-cell suspension of CD34+ cells were processed according to the NFκB translocation kit protocol (Luminex Corporation).

Instrument

BD FACSCanto II
BD LSRFortessa
BD LSR II
BD FACS Aria II
BD FACS Aria III

Software

BD FACSCanto II; software BD FACSDiva version 9.0
BD LSRFortessa; software BD FACSDiva version 8.0.1
BD LSR II, software BD FACSDiva version 9.0
BD FACS Aria II; software BD FACSDiva version 6.1.3
BD FACS Aria III; software BD FACSDiva version 8.0.1

Cell population abundance

The purity of lineage-negative immune magnetically enriched cells was higher than 65%, and it was determined by flow cytometry.
The purity of human CD34+ immune magnetically enriched cells was checked only after extraction of abundant numbers of cells, and it was higher than 98% as determined by flow cytometry.
FACS-sorted cells were sorted in "purity" precision mode using BD FACSAria III. Quality controls of the purity of test FACS-sorted cells were carried out systematically, using flow cytometry. Based on these quality controls, the purity of FACS-sorted cells is expected to be higher than 90%.

Gating strategy

Based on cell size (forward scatter, FSC) and cell granularity (side scatter, SSC), cell debris was excluded. Subsequently, FSC and SSC doublets were excluded. Live cells were gated based on DAPI exclusion. Cell debris, cell doublets and dead cell exclusions were applied in all flow cytometry/FACS-sorting experiment analyses. Positive populations were determined based on unstained and/or isotype controls and/or single stainings in the set-up of new staining protocols. In most cases, compensation was performed manually. Compensation beads were used in the set-up of complex multicolor staining protocols.
We are showing a representative dot plot example at first iteration for each cell population analyzed in the manuscript, in the main figure or in the associated supplementary figures.

☒ Tick this box to confirm that a figure exemplifying the gating strategy is provided in the Supplementary Information.
